# An Ultrasensitive Biosensor for Probing Subcellular Distribution and Mitochondrial Transport of l‐2‐Hydroxyglutarate

**DOI:** 10.1002/advs.202404119

**Published:** 2024-07-15

**Authors:** Zhaoqi Kang, Shuang Hou, Kaiyu Gao, Yidong Liu, Ning Zhang, Zhiqing Fang, Wen Zhang, Xianzhi Xu, Rong Xu, Chuanjuan Lü, Cuiqing Ma, Ping Xu, Chao Gao

**Affiliations:** ^1^ State Key Laboratory of Microbial Technology Shandong University Qingdao 266237 P. R. China; ^2^ Department of Breast Surgery General Surgery Qilu Hospital Cheeloo College of Medicine Shandong University Jinan 250100 P. R. China; ^3^ Department of Urology Qilu Hospital Cheeloo College of Medicine Shandong University Jinan 250100 P. R. China; ^4^ Institute of Medical Sciences The Second Hospital Cheeloo College of Medicine Shandong University Jinan 250100 P. R. China; ^5^ State Key Laboratory of Microbial Metabolism Joint International Research Laboratory of Metabolic & Developmental Sciences, and School of Life Sciences & Biotechnology Shanghai Jiao Tong University Shanghai 200240 P. R. China

**Keywords:** biosensor, l‐2‐hydroxyglutarate, metabolism, mitochondrial transport, point‐of‐care testing

## Abstract

l‐2‐Hydroxyglutarate (l‐2‐HG) is a functionally compartmentalized metabolite involved in various physiological processes. However, its subcellular distribution and mitochondrial transport remain unclear owing to technical limitations. In the present study, an ultrasensitive l‐2‐HG biosensor, sfLHGFR_H_, composed of circularly permuted yellow fluorescent protein and l‐2‐HG‐specific transcriptional regulator, is developed. The ability of sfLHGFR_H_ to be used for analyzing l‐2‐HG metabolism is first determined in human embryonic kidney cells (HEK293FT) and macrophages. Then, the subcellular distribution of l‐2‐HG in HEK293FT cells and the lower abundance of mitochondrial l‐2‐HG are identified by the sfLHGFR_H_‐supported spatiotemporal l‐2‐HG monitoring. Finally, the role of the l‐glutamate transporter SLC1A1 in mitochondrial l‐2‐HG uptake is elucidated using sfLHGFR_H_. Based on the design of sfLHGFR_H_, another highly sensitive biosensor with a low limit of detection, sfLHGFR_L_, is developed for the point‐of‐care diagnosis of l‐2‐HG‐related diseases. The accumulation of l‐2‐HG in the urine of patients with kidney cancer is determined using the sfLHGFR_L_ biosensor.

## Graphical abstract

In the present study, an ultrasensitive biosensor, composed of circularly permuted superfolder yellow fluorescent protein cpSFYFP and l‐2‐hydroxyglutarate (l‐2‐HG)‐specific transcriptional regulator LhgR, is developed for real‐time imaging of l‐2‐HG in living cells. The lower abundance of the mitochondrial l‐2‐HG pool and the first mitochondrial l‐2‐HG transporter are discovered based on the high spatiotemporal resolution l‐2‐HG assay supported by this biosensor.

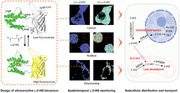

## 1 Introduction

l‐2‐Hydroxyglutarate (l‐2‐HG) is an essential endogenous metabolite involved in various physiological processes.^[^ ^1^ ^]^ For example, l‐2‐HG can maintain redox homeostasis under hypoxic conditions by inhibiting glycolysis that occurs in the cytosol,^[^ ^2^, ^3^, ^4^ ^]^ and coordinate immune responses of CD8^+^ T‐lymphocytes through altering DNA and histone methylation proceeded in the nucleus.^[^ ^5^ ^]^ Interestingly, l‐2‐HG also inhibits the ATP synthase located in the mitochondria and potentially extends the lifespan of *Caenorhabditis elegans*.^[^ ^6^ ^]^ Since l‐2‐HG is functionally compartmentalized, it is essential to identify the physical distribution of this metabolite in different organelles. However, l‐2‐HG is easily redistributed or catabolized during organelle separation and sample preparation. Real‐time and in situ assays of l‐2‐HG are required to provide deeper insights into the subcellular distribution of l‐2‐HG and its physiological functions.

l‐2‐HG is produced in the cytosol (mediated by l‐lactate dehydrogenase A [LDHA] and l‐malate dehydrogenase 1 [MDH1]) and mitochondria (mediated by l‐malate dehydrogenase 2 [MDH2]) in most cell types.^[^ ^2^, ^3^, ^4^ ^]^ l‐2‐HG dehydrogenase (L2HGDH), a key enzyme involved in l‐2‐HG degradation, is located in the inner mitochondrial membrane.^[^ ^7^ ^]^ As a competitive inhibitor of 2‐ketoglutarate (2‐KG)‐dependent dioxygenases,^[^ ^8^, ^9^ ^]^ supraphysiologically accumulated l‐2‐HG affects epigenetic modifications, DNA repair, and cell fate and participates in the pathogenesis of various diseases, including l‐2‐hydroxyglutaric aciduria and kidney cancer.^[^ ^10^, ^11^, ^12^, ^13^ ^]^ Thus, cytosolic l‐2‐HG should be transported into the mitochondria and decomposed by L2HGDH to prevent its excessive accumulation. However, the specific mechanisms underlying the mitochondrial l‐2‐HG uptake remain unclear.^[^ ^1^ ^]^

Genetically encoded fluorescent biosensors can form intrinsic fluorophores in vivo, enabling the spatiotemporal monitoring of metabolite dynamics by targeting specific subcellular organelles.^[^ ^14^, ^15^, ^16^ ^]^ Thus, these biosensors have been widely utilized in revealing the subcellular distribution and transport mechanism of different metabolites.^[^ ^17^, ^18^, ^19^, ^20^, ^21^ ^]^ For example, the enrichment of l‐lactate in mammalian mitochondria was recently revealed by using the circularly permuted fluorescent protein (cpFP)‐based l‐lactate biosensor FiLa.^[^ ^18^ ^]^ The role of the mitochondrial 2‐KG carrier (OGC, SLC25A11) in mitochondrial itaconate efflux was confirmed using the itaconate biosensor BioITA.^[^ ^20^ ^]^ A Förster resonance energy transfer (FRET) technology‐based l‐2‐HG biosensor, LHGFR, has been previously developed.^[^ ^22^ ^]^ However, LHGFR exhibited inappropriate affinity and a low response magnitude to l‐2‐HG and thus was unsuitable for distinguishing the distribution of l‐2‐HG in different subcellular compartments.^[^ ^22^ ^]^

In this study, an l‐2‐HG biosensor based on the cpFP and the l‐2‐HG‐specific transcriptional regulator LhgR, denoted as sfLHGFR_H_ (single fluorescent protein‐based l‐2‐HG‐sensing fluorescent reporter with high detection range), was developed. sfLHGFR_H_ exhibited high responsiveness and appropriate affinity for l‐2‐HG detection in vivo. Then, the suitability of sfLHGFR_H_ for analysis of l‐2‐HG metabolism in HEK293FT cells and macrophages was ascertained. The lower abundance of mitochondrial l‐2‐HG pool and the role of SLC1A1 (mitochondrial l‐glutamate transporter) in mitochondrial l‐2‐HG uptake were elucidated based on the sfLHGFR_H_‐supported high spatiotemporal resolution l‐2‐HG assay. In addition, a highly sensitive l‐2‐HG biosensor with a low limit of detection (LOD), sfLHGFR_L_, was developed for point‐of‐care detection of l‐2‐HG in clinical body fluids. The accumulation of l‐2‐HG in the urine of patients with kidney cancer was confirmed using sfLHGFR_L_.

## 2 Result

### 2.1 Design and Optimization of l‐2‐HG Biosensor sfLHGFR_H_

**Figure 1:**
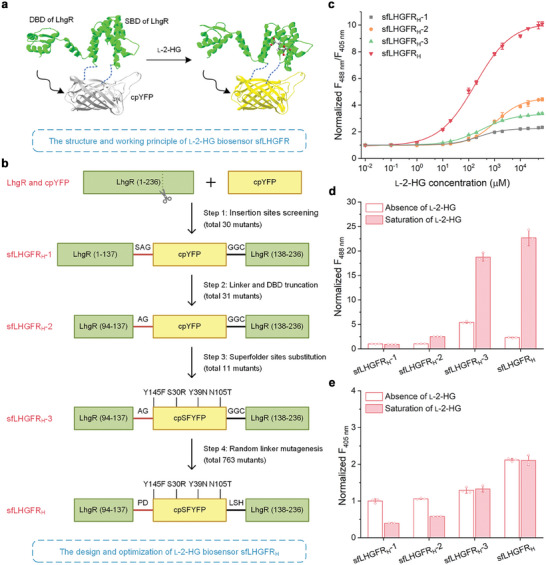
Design of l‐2‐HG biosensor sfLHGFR_H_. a) Schematic representation of the working principle of sfLHGFR. DBD, DNA‐binding domain. SBD, substrate‐binding domain. b) Schematic representation of the development of sfLHGFR_H_. cpSFYFP, cpYFP with four superfolder sites. The numbering of the substituted amino acids in cpYFP corresponds to the sequence of the original YFP. c) Comparison of the dose‐response curves of sfLHGFR_H_‐1, sfLHGFR_H_‐2, sfLHGFR_H_‐3, and sfLHGFR_H_ for l‐2‐HG. Data were normalized to the initial ratio. d,e) Comparison of the fluorescence intensities of sfLHGFR_H_‐1, sfLHGFR_H_‐2, sfLHGFR_H_‐3, and sfLHGFR_H_ with excitation at 488 nm d) and 405 nm e). Data were normalized to the fluorescence intensity of sfLHGFR_H_‐1 in the absence of l‐2‐HG. All data shown are means ± standard deviations (s.d.) (*n* ≥ 3 independent experiments).

The cpFP‐based biosensors consist of an analyte‐sensing moiety and cpFP. The binding of the analyte to the sensing moiety may elicit dramatic changes in the conformation and fluorescence spectrum of cpFP, resulting in the ultrasensitive detection of various small molecules.^[^ ^18^, ^19^, ^20^, ^23^, ^24^ ^]^ The transcriptional regulator LhgR in *Pseudomonas putida* W619 can specifically bind to l‐2‐HG,^[^ ^22^ ^]^ making it a promising sensing moiety for the development of a cpFP‐based l‐2‐HG biosensor (sfLHGFR). LhgR consists of 10 α‐helices. The α−1 to α−4 and α−5 to α−10 constitute the DNA‐binding domain and ligand‐binding domain, respectively (Figure S1, Supporting Information). In the present study, sfLHGFR was constructed and optimized using a four‐step workflow (**Figure** **1**a,b). In Step 1, a total of 30 biosensor variants were constructed by inserting a circularly permuted yellow fluorescent protein (cpYFP) between two subunits of LhgR or LhgR(D2) with truncated DNA‐binding domain or into the loop region between adjacent α‐helices in the ligand‐binding domain of LhgR (Figure S2a, Supporting Information). Then, the fluorescence ratio (F_488 nm_/F_405 nm_, the ratio of fluorescence emission at 528 nm with 488 and 405 nm excitation) changes of these 30 biosensor variants in response to l‐2‐HG addition were determined. As shown in Figure 1c–e and Figure S2 (Supporting Information), the variant sfLHGFR_137D/138F_ obtained by inserting cpYFP into the 137D/138F site of LhgR exhibited the highest Δ*R*_*max*_ (maximum ratio change) of 133.26 ± 5.62% to l‐2‐HG. Mechanistically, the binding of l‐2‐HG to LhgR led to conformational changes in the backbone and side chains near the insertion site of cpYFP. These changes could be further transmitted to the exposed chromophore of cpYFP, and finally alter its optical properties (Figure 1a).

The l‐2‐HG concentrations ranged from 25 µm to millimolar in mammalian cells.^[^ ^4^, ^5^ ^]^ sfLHGFR_137D/138F_ exhibited an apparent dissociation constant (*K_d_*) of 234.11 ± 13.74 µm, which is appropriate for in vivo detection of high‐level intracellular l‐2‐HG (Figure S2c,d, Supporting Information). Thus, sfLHGFR_137D/138F_ (denoted as sfLHGFR_H_‐1) was systematically optimized. In Step 2, sfLHGFR_H_‐2 with a Δ*R_max_* of 349.16 ± 11.94% was screened from 31 variants constructed by truncating the linker between cpYFP and LhgR in sfLHGFR_H_‐1 and removing the DNA‐binding domain of LhgR (Figure S3a–c, Supporting Information). Although sfLHGFR_H_‐2 expressed in HEK293FT cells responded to exogenous l‐2‐HG, its weak fluorescence limited its subsequent applications (Figure S3d,e, Supporting Information). In Step 3, cpYFP in sfLHGFR_H_‐2 was replaced with different fluorescent proteins; however, the response magnitudes of the obtained biosensors were substantially reduced (Figure S4a–c, Supporting Information). Thus, four superfolder sites (S30R, Y39N, N105T, and Y145F) from superfolder GFP were introduced into cpYFP to enhance fluorescence brightness,^[^ ^25^ ^]^ and the obtained biosensor sfLHGFR_H_‐3 retained a high Δ*R_max_* of 237.53 ± 6.61% and exhibited a significantly improved fluorescence brightness. Specifically, the brightness increased by 5.28‐fold (Ex: 488 nm) and 1.22‐fold (Ex: 405 nm) for purified sfLHGFR_H_‐3 and increased by 20.93‐fold (Ex: 488 nm) and 4.53‐fold (Ex: 405 nm) for sfLHGFR_H_‐3 expressed in HEK293FT cells (Figure 1d,e; Figure S4d–i, Supporting Information). In Step 4, the performance of sfLHGFR_H_‐3 was further optimized using random linker mutagenesis in combination with high‐throughput screening (Figure S5a, Supporting Information). Twelve mutants with increased responses to l‐2‐HG were screened from the 763 mutants (Figure S5b, Supporting Information). Then, the Δ*R_max_*, brightness in HEK293FT cells, and response magnitude to l‐2‐HG in HEK293FT cells were compared among these 12 mutants (Figure S5c–e, Supporting Information). The best variant #15 with high Δ*R_max_* (909.23 ± 16.19%) in vitro, brightness in HEK293FT cells (16.75‐fold higher than that of sfLHGFR_H_‐2 under 488 nm excitation), and sensitivity to l‐2‐HG in HEK293FT cells was selected and denoted as sfLHGFR_H_ for intracellular applications (Figure 1c–e; Figure S5c–g, Supporting Information). The amino acid sequence of sfLHGFR_H_ is shown in Figure S6 (Supporting Information).

### 2.2 Characterization of sfLHGFR_H_

**Figure 2:**
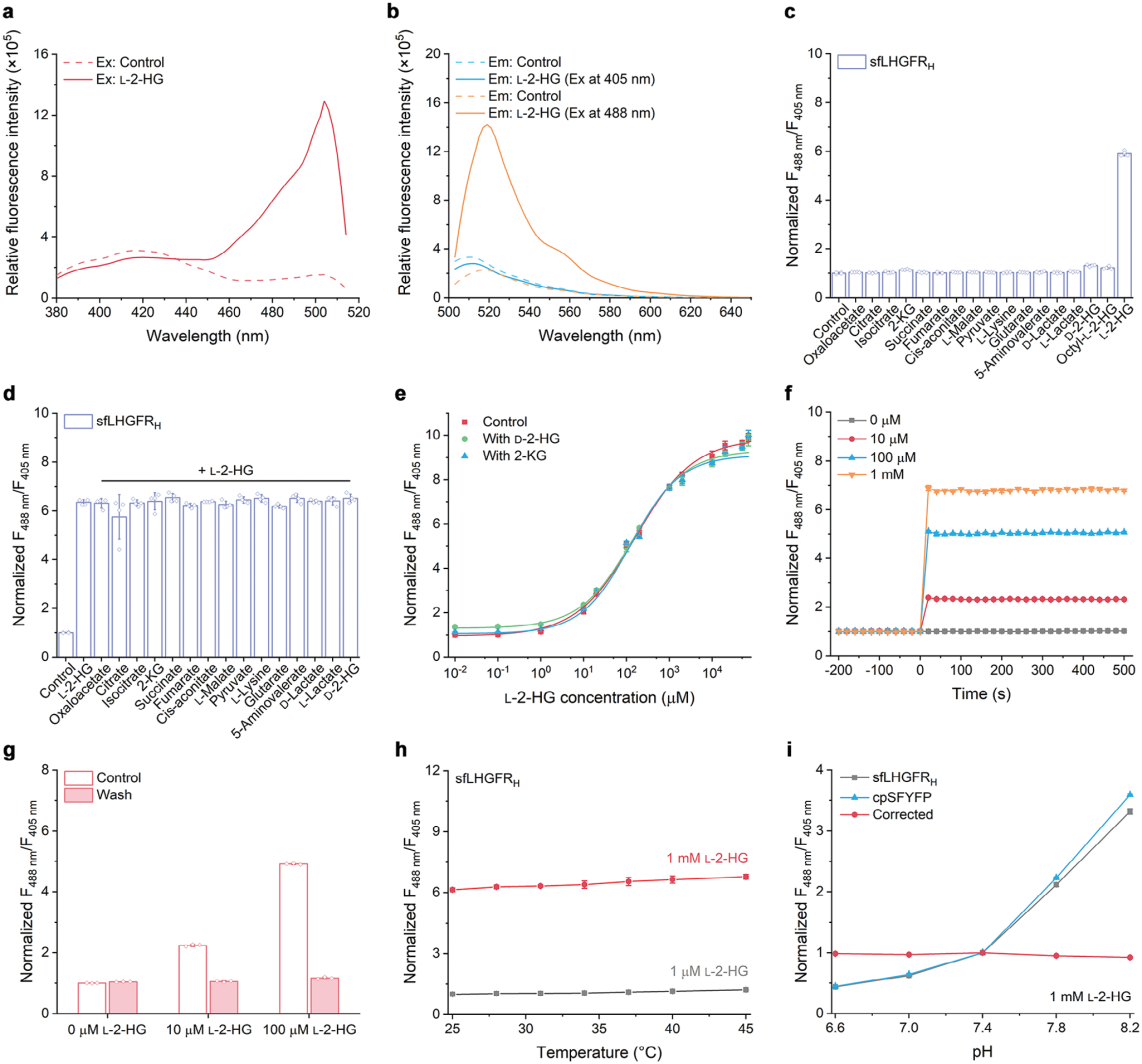
Characterization of sfLHGFR_H_. a) Fluorescence excitation spectra of sfLHGFR_H_ with or without the addition of 1 mm l‐2‐HG. b) Fluorescence emission spectra of sfLHGFR_H_ with or without the addition of 1 mm l‐2‐HG at 405 nm or 488 nm excitation. c) Specificity analysis of sfLHGFR_H_. The fluorescence ratios of sfLHGFR_H_ were determined in the presence of 250 µm indicated metabolites. Data were normalized to the control. d) Influence of various metabolites on detection of l‐2‐HG by sfLHGFR_H_. The fluorescence ratios of sfLHGFR_H_ were determined in the absence of any metabolite (control), in the presence of only l‐2‐HG (l‐2‐HG), and in the presence of l‐2‐HG and 250 µm indicated metabolites. Data were normalized to the control. e) Dose‐response curves of sfLHGFR_H_ for increasing concentrations of l‐2‐HG in the presence of 250 µm d‐2‐HG or 2‐KG. Data were normalized to the initial ratio without d‐2‐HG and 2‐KG. f) Kinetics of the response of sfLHGFR_H_ to l‐2‐HG. l‐2‐HG were added at time point zero. Data were normalized to the initial ratio without l‐2‐HG. g) Reversibility analysis of sfLHGFR_H_. The fluorescence ratios of sfLHGFR_H_ after l‐2‐HG addition and subsequent removal were recorded. Data were normalized to the control with 0 µm l‐2‐HG. h) Temperature‐stability analysis of sfLHGFR_H_. Data were normalized to the initial ratio with 1 µm l‐2‐HG. i) pH‐correction of the fluorescence ratio of sfLHGFR_H_ by cpSFYFP in the presence of 1 mm l‐2‐HG. Data were normalized to the ratio at pH 7.4. All data shown are means ± s.d. (*n* ≥ 3 independent experiments).

sfLHGFR_H_ exhibited two excitation peaks ≈405 and 488 nm and a single emission peak ≈528 nm. The binding of 1 mm l‐2‐HG to sfLHGFR_H_ resulted in a 6.18‐fold increase and a 1.14‐fold decrease in fluorescence intensity upon excitation at 488 and 405 nm, respectively (**Figure** **2**a,b). Then, 250 µm l‐2‐HG or its structural analogs, including octyl‐l‐2‐HG, d‐lactate, l‐lactate, d‐2‐HG, and a series of intermediates of the TCA cycle and the l‐lysine catabolism, were mixed with purified sfLHGFR_H_, respectively, to analyze the specificity of sfLHGFR_H_. As shown in Figure 2c, only l‐2‐HG significantly increased the fluorescence ratio of sfLHGFR_H_, while other analogs could not. In addition, the detection of l‐2‐HG by sfLHGFR_H_ was not affected by the presence of these analogs (Figure 2d), and d‐2‐HG and 2‐KG did not influence the dose‐response curve of sfLHGFR_H_ for l‐2‐HG (Figure 2e).

The response of an ideal biosensor for visualizing intracellular l‐2‐HG dynamics should be instantaneous and reversible. To examine the response speed of sfLHGFR_H_ to l‐2‐HG, changes in the fluorescence ratio of sfLHGFR_H_ were continuously measured before and after l‐2‐HG addition. As shown in Figure 2f, l‐2‐HG addition elicited an immediate (within 20 s) increase in the fluorescence ratio of sfLHGFR_H_ to its maximum value, which remained constant throughout subsequent assays. The reversibility of sfLHGFR_H_ to l‐2‐HG was assayed by measuring the fluorescence ratio changes after adding l‐2‐HG and removing l‐2‐HG in the detection system using ultrafiltration centrifuge tubes. As shown in Figure 2g, the elevated fluorescence ratio of sfLHGFR_H_ induced by the addition of l‐2‐HG recovered to basal levels after l‐2‐HG removal.

The temperature and pH stability of sfLHGFR_H_ for l‐2‐HG detection were also studied. As shown in Figure 2h, the detection of l‐2‐HG by sfLHGFR_H_ remained unaffected between 25 and 40 °C. Consistent with other cpFP‐based biosensors,^[^ ^18^, ^19^ ^]^ the response of sfLHGFR_H_ to l‐2‐HG was susceptible to pH variations (Figure S7a,b, Supporting Information). Thus, cpSFYFP (cpYFP with four superfolder sites), a control biosensor that did not respond to l‐2‐HG and exhibited pH‐sensitive properties similar to those of sfLHGFR_H_, was used in parallel with sfLHGFR_H_ (Figure S7c,d, Supporting Information). As shown in Figure 2i and Figure S7e, (Supporting Information), the fluorescence ratio of sfLHGFR_H_ corrected by cpSFYFP remained stable between pH 6.6 and 8.2.

### 2.3 Visualization of l‐2‐HG Metabolism in HEK293FT Cells Using sfLHGFR_H_

**Figure 3:**
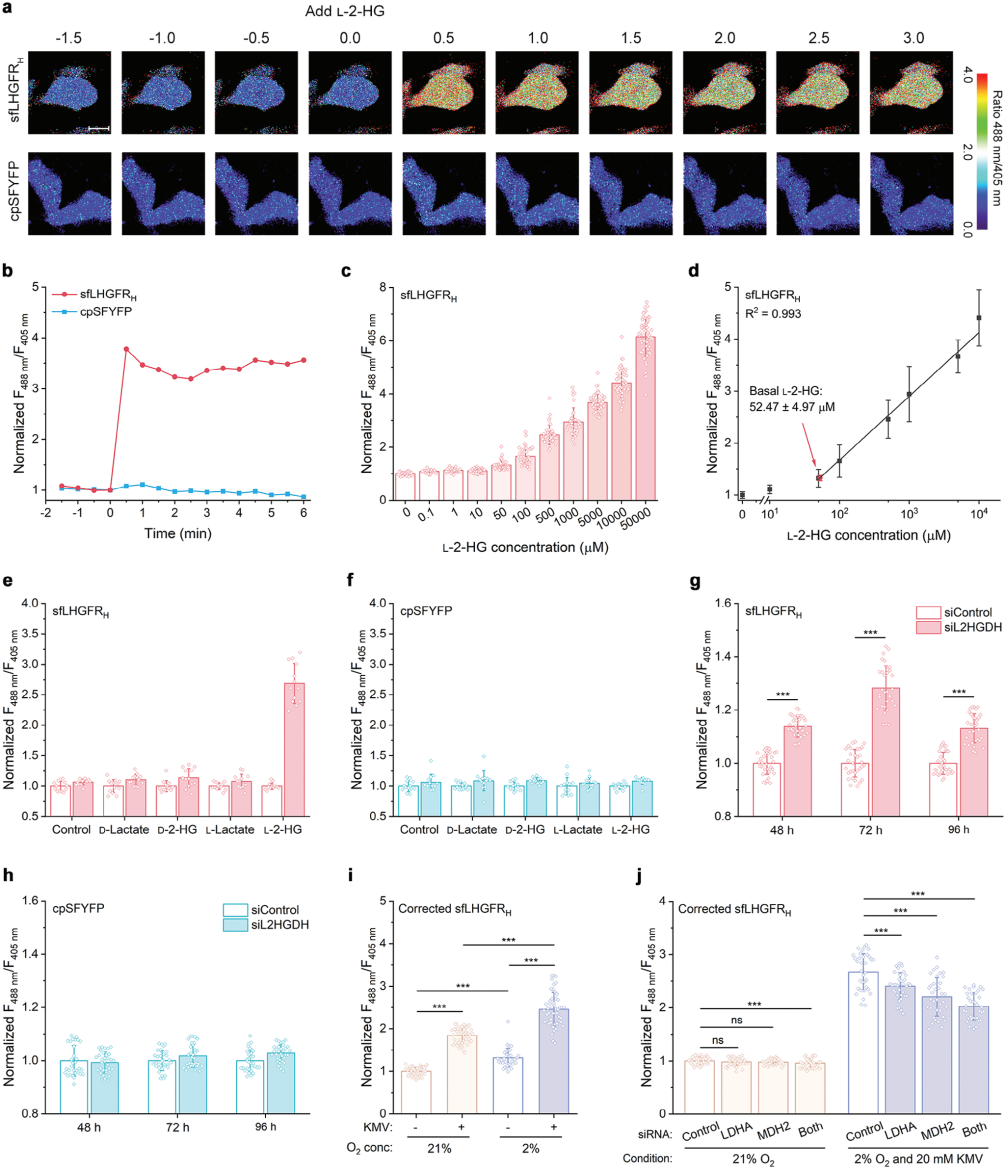
Visualization of l‐2‐HG metabolism in living cells using sfLHGFR_H_. a,b) Sequential images a) and quantitative data b) of sfLHGFR_H_ and cpSFYFP expressed in HEK293FT cells in response to 5 mm l‐2‐HG addition. Scale bar, 10 µm. Data in panel b were normalized to the ratio at time point zero. c,d) Dose‐dependent response c) and calibration curve d) of sfLHGFR_H_ for increasing concentrations of l‐2‐HG. Data were normalized to the initial ratio (*n* ≥ 13 cells). e,f) Specificity analysis of sfLHGFR_H_ e) or cpSFYFP f) expressed in HEK293FT cells. The fluorescence ratios of sfLHGFR_H_ and cpSFYFP before (blank column) and after (colored column) addition of the indicated metabolites were recorded. Data were normalized to the ratio before the addition of any metabolites (*n* = 13 cells). g,h) Identification of L2HGDH function using sfLHGFR_H_. The fluorescence ratios of sfLHGFR_H_ g) and cpSFYFP h) were determined 48, 72, and 96 h after treatment with negative siRNA or siRNA targeting L2HGDH. Data were normalized to the control (*n* = 32 cells). i) Analysis of hypoxia‐induced l‐2‐HG production using sfLHGFR_H_. The fluorescence ratios of sfLHGFR_H_ were determined 24 h after incubation of cells with 21% oxygen or 2% oxygen as well as in the absence or presence of 20 mm α‐keto‐β‐methylvaleric acid (KMV). Data were corrected by cpSFYFP and normalized to the normoxic condition without KMV (*n* ≥ 35 cells). j) Analysis of the anabolic mechanism of l‐2‐HG using sfLHGFR_H_. sfLHGFR_H_‐expressing HEK293FT cells were treated with different siRNAs for 24 h, and then cultured under the indicated conditions for another 24 h. Data were corrected by cpSFYFP and normalized to the normoxic condition treated with negative siRNA (*n* = 35 cells). All data shown are means ± s.d. *** *p* < 0.001; ns, no significant difference (*p* ≥ 0.05) in the two‐tailed *t*‐test.

Subsequently, sfLHGFR_H_ was used for in vivo l‐2‐HG detection. sfLHGFR_H_ and cpSFYFP were expressed in HEK293FT cells, respectively (Figure S8a, Supporting Information). The fluorescence of sfLHGFR_H_ without any additional localization sequences was uniform throughout the cell (**Figure** **3**a). Digitonin at a concentration of 80 µm was added to the imaging medium to permeabilize the cell membrane of HEK293FT cells and remove endogenous l‐2‐HG. A decrease in the fluorescence ratio of sfLHGFR_H_ was observed after digitonin treatment (Figure S8b, Supporting Information). Next, 5 mm l‐2‐HG was added to the imaging medium containing digitonin‐permeabilized cells. As shown in Figure 3a,b, sfLHGFR_H_ rapidly responded to l‐2‐HG addition, with an increased ratio of fluorescence with excitation at 488 nm and 405 nm (F_488 nm_/F_405 nm_), whereas cpSFYFP responded negligibly to l‐2‐HG addition.

The response of sfLHGFR_H_ expressed in HEK293FT cells to gradient concentrations of l‐2‐HG was dose‐dependent (Figure S9, Supporting Information), and a 4.41‐fold change in F_488 nm_/F_405 nm_ was observed in the presence of 10 mm l‐2‐HG (Figure 3c). Based on the established calibration curve of intracellular sfLHGFR_H_ for l‐2‐HG, basal l‐2‐HG concentration in HEK293FT cells under normal conditions was calculated to be ≈52.47 ± 4.97 µm (Figure 3d), which is close to previous results obtained using LC‐MS/MS after sophisticated sample handling.^[^ ^4^ ^]^ In addition, a series of l‐2‐HG analogs, including d‐lactate, l‐lactate, and d‐2‐HG, were added to the imaging medium of sfLHGFR_H_‐expressing HEK293FT cells to analyze the specificity of sfLHGFR_H_ in vivo. As shown in Figure 3e,f, no significant changes in the fluorescence ratios were observed in the presence of these analogs.

L2HGDH is a key enzyme involved in the catabolism of l‐2‐HG in mammalian cells.^[^ ^7^ ^]^ As shown in Figure 3g,h and Figure S10a, (Supporting Information), the fluorescence ratio of sfLHGFR_H_ increased by 28.23% after treatment with siRNA targeting L2HGDH for 72 h, whereas the ratio of cpSFYFP remained constant under the same conditions. l‐2‐HG was previously demonstrated to be produced under hypoxic conditions by the LDHA‐ and MDH2‐mediated “promiscuous” reduction activity toward 2‐KG.^[^ ^2^, ^3^, ^4^ ^]^ Endogenous fluctuations in l‐2‐HG levels under hypoxic conditions were also investigated using sfLHGFR_H_. As shown in Figure 3i and Figure S11a (Supporting Information), treatment with 2% O_2_ increased the fluorescence ratio of sfLHGFR_H_ by 31.88%. Co‐treatment with α‐keto‐β‐methylvaleric acid (KMV), a small molecule inhibitor of 2‐KG dehydrogenase complex, or with dimethyl‐α‐KG (DMαKG), resulted in a 145.91% or 102.02% increase in the fluorescence ratio of sfLHGFR_H_, respectively (Figure 3i; Figure S11a, Supporting Information). The knockdown of either LDHA or MDH2 decreased the upward trend of the fluorescence ratio of sfLHGFR_H_ under the abovementioned conditions, indicating the critical role of LDHA and MDH2 in l‐2‐HG anabolism (Figure 3j; Figures S10b, and S11b, Supporting Information).

### 2.4 Monitoring of l‐2‐HG in Macrophages with Different Polarization States Using sfLHGFR_H_

**Figure 4:**
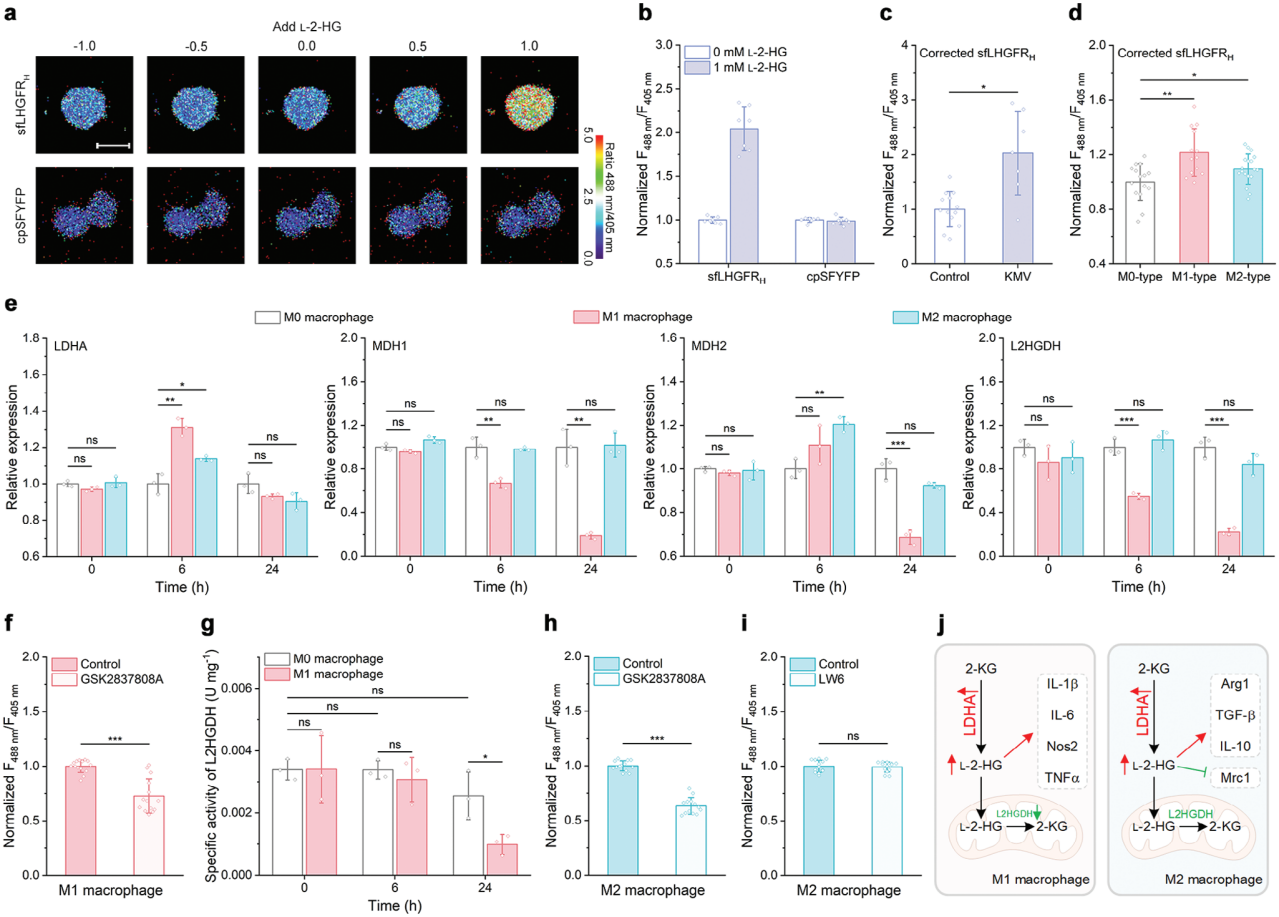
Metabolism and function analysis of l‐2‐HG in macrophages using sfLHGFR_H_. a,b) Sequential images a) and quantitative data b) of sfLHGFR_H_ or cpSFYFP in macrophages in response to 1 mm l‐2‐HG addition. Scale bar, 10 µm. Data in panel b were normalized to the ratio without l‐2‐HG (*n* = 7 cells). c) Response of sfLHGFR_H_ to KMV‐mediated endogenous l‐2‐HG accumulation. Data were corrected by cpSFYFP and normalized to the control (*n* = 13 and 7 cells from left to right). d) Differences in l‐2‐HG concentrations between differently polarized macrophages measured by sfLHGFR_H_. Data were corrected by cpSFYFP and normalized to M0 macrophages (*n* ≥ 13 cells). e) qPCR analysis of the expression of key enzymes for l‐2‐HG metabolism in differently polarized macrophages. Data were analyzed 0, 6, and 24 h after inducer stimulation (*n* = 3 independent experiments). f) Identification of the function of LDHA in l‐2‐HG anabolism in M1 macrophages using sfLHGFR_H_. Data were corrected by cpSFYFP and normalized to control (*n* = 14 cells). g) Activities of L2HGDH in M0 and M1 macrophages. Data were detected 0, 6, and 24 h after inducer stimulation (*n* = 3 independent experiments). h,i) Identification of the functions of LDHA h) and MDH2 i) in l‐2‐HG anabolism in M2 macrophages using sfLHGFR_H_. Data were corrected by cpSFYFP and normalized to control (*n* = 13 cells). j) Schematic representation of the metabolic pathway and biological function of l‐2‐HG in M1 and M2 macrophages. All data shown are means ± s.d. * *p* < 0.05; ** *p* < 0.01; *** *p* < 0.001; ns, no significant difference (*p* ≥ 0.05) in the two‐tailed *t*‐test.

Macrophages participate in the immune responses against pathogenic infections and tissue damage. Resting macrophages (M0) can be polarized into classically activated (M1) and alternatively activated (M2) cells.^[^ ^26^, ^27^, ^28^ ^]^ l‐2‐HG has recently been found to accumulate in M1 macrophages, resulting in altered expression patterns of multiple proinflammatory cytokines.^[^ ^29^, ^30^ ^]^ However, the metabolic mechanisms and functions of l‐2‐HG in macrophages remain unclear. Therefore, sfLHGFR_H_ was expressed in murine macrophages to investigate the metabolic features of l‐2‐HG in macrophages. As shown in **Figure** **4**a–c, sfLHGFR_H_ expressed in macrophages responded to exogenous l‐2‐HG addition and KMV‐mediated endogenous l‐2‐HG accumulation. Then, lipopolysaccharide (LPS) + interferon‐γ (IFN‐γ) or interleukin‐4 (IL‐4) treatment was administered to induce M0 cell polarization into M1 and M2 cells, with the expression of their respective marker genes (Figures S12 and S13, Supporting Information). The distribution of l‐2‐HG in differently polarized macrophages was analyzed using sfLHGFR_H_ and LC‐MS/MS. As shown in Figure 4d and Figure S14a, (Supporting Information), greater l‐2‐HG accumulation was observed in both M1 and M2 macrophages using sfLHGFR_H_ and LC‐MS/MS.

Octyl‐l‐2‐HG at a concentration of 500 µm was exogenously added into the cultures of M1 and M2 macrophages, and changes in the expression levels of relevant marker genes were analyzed. As shown in Figures S12 and S13 (Supporting Information), l‐2‐HG addition promoted the expression of IL‐1β, IL‐6, Nos2, and TNFα in M1 macrophages; and promoted the expression of Arg1, TGF‐β, and IL‐10 but inhibited the expression of Mrc1 in M2 macrophages. The expression levels of enzymes possibly involved in l‐2‐HG anabolism and catabolism, including LDHA, MDH1, MDH2, mitochondrial malic enzyme 2 (ME2),^[^ ^31^ ^]^ and L2HGDH, were analyzed to identify the metabolic mechanism of l‐2‐HG in macrophages with different polarization states. As shown in Figure 4e and Figure S15 (Supporting Information), LDHA expression was upregulated in the M1 macrophages. The pharmacological inhibition of LDHA by GSK2837808A reduced the fluorescence ratio of sfLHGFR_H_ in M1 macrophages (Figure 4f).^[^ ^32^ ^]^ In addition, downregulation of L2HGDH expression and a decrease in L2HGDH activity were detected after M1 polarization (Figure 4e,g). Thus, elevated LDHA levels and decreased L2HGDH expression may be the major causes of the accumulation of l‐2‐HG in M1 macrophages (Figure 4j). Upregulation of LDHA and MDH2 expression was observed in M2 macrophages (Figure 4e). Exogenous addition of GSK2837808A reduced the fluorescence ratio of sfLHGFR_H_ in M2 macrophages (Figure 4h), whereas the ratio of sfLHGFR_H_ remained unaffected by treatment with LW6, an inhibitor of MDH2 (Figure 4i).^[^ ^33^ ^]^ These results suggested that elevated LDHA levels play a critical role in l‐2‐HG accumulation in M2 macrophages, whereas MDH2 may not contribute to l‐2‐HG synthesis (Figure 4j). All sfLHGFR_H_‐based l‐2‐HG measurements in macrophages were validated using LC‐MS/MS and similar results were obtained (Figure S14b,c, Supporting Information).

### 2.5 Analysis of the Subcellular Distribution of l‐2‐HG Using sfLHGFR_H_

**Figure 5:**
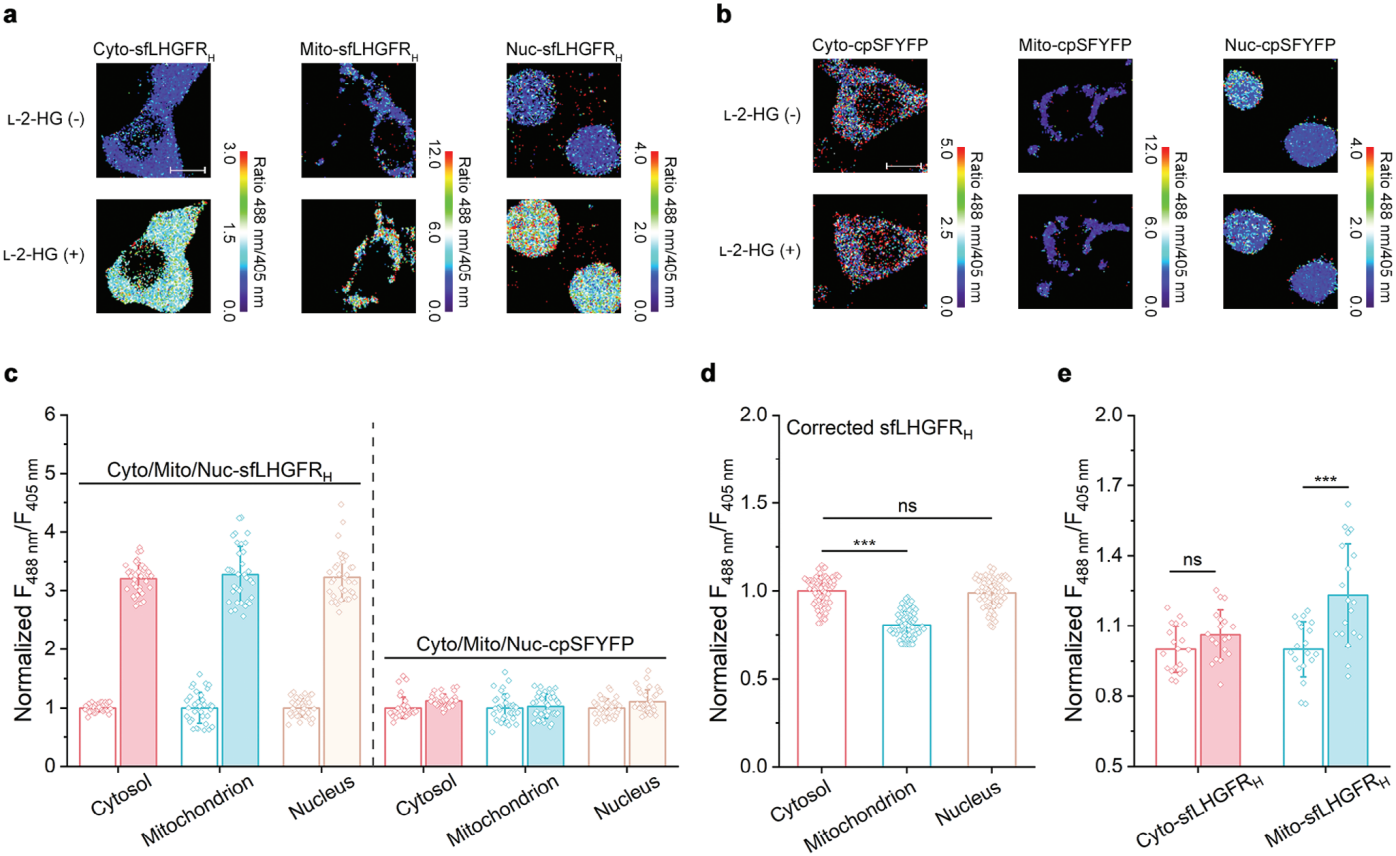
Analysis of subcellular distribution of l‐2‐HG using sfLHGFR_H_. a,b) Confocal microscopy imaging of a single HEK293FT cell expressing Cyo‐sfLHGFR_H_, Mito‐sfLHGFR_H_, Nuc‐sfLHGFR_H_ a), or Cyo‐cpSFYFP, Mito‐cpSFYFP, Nuc‐cpSFYFP b) in response to exogenous 5 mm l‐2‐HG addition. Scale bar, 10 µm. c) Response of different subcellular localized sfLHGFR_H_ or cpSFYFP to exogenous l‐2‐HG addition. The fluorescence ratios of sfLHGFR_H_ and cpSFYFP before (blank column) and after (colored column) addition of 5 mm l‐2‐HG were recorded. Data were normalized to the ratio before l‐2‐HG addition (*n* = 35 cells). d) Subcellular distribution of l‐2‐HG measured by sfLHGFR_H_. Data were corrected by cpSFYFP and normalized to the ratio of Cyo‐sfLHGFR_H_ (*n* = 74 cells). e) Identification of ME2 function using sfLHGFR_H_. The fluorescence ratios of sfLHGFR_H_ with (colored column) or without (blank column) ME2 overexpression were recorded. Data were normalized to the control (*n* = 19 cells). All data shown are means ± s.d. *** *p* < 0.001; ns, no significant difference (*p* ≥ 0.05) in the two‐tailed *t*‐test.

The sequences for nuclear exclusion, mitochondrial localization, and nuclear localization were fused to the N‐, N‐, and C‐terminus of sfLHGFR_H_ to realize its subcellular positioning in HEK293FT cells. As shown in Figure S16 (Supporting Information), the fluorescence of sfLHGFR_H_ was successfully restricted to the cytosol, mitochondria, and nucleus by introducing corresponding localization signals. Next, 5 mm l‐2‐HG was added to the imaging medium of digitonin‐pre‐permeabilized HEK293FT cells. As shown in **Figure** **5**a–c, sfLHGFR_H_ expressed in different subcellular compartments exhibited identical responses to exogenous l‐2‐HG addition, whereas the fluorescence ratio of cpSFYFP remained unaffected.

Then, the subcellular distribution of l‐2‐HG was investigated by comparing the fluorescence ratios of the differentially localized sfLHGFR_H_ in resting HEK293FT cells. The presence of l‐2‐HG in the nucleus was directly detected (Figure 5d). This result is consistent with those of previous reports showing that l‐2‐HG inhibits various 2‐KG‐dependent dioxygenases in the nucleus.^[^ ^1^, ^5^, ^8^, ^9^, ^11^ ^]^ In addition, the fluorescence ratio of sfLHGFR_H_ localized in the nucleus was similar to that localized in the cytosol (Figure 5d). The uniform distribution of l‐2‐HG in the cytosol and the nucleus may be attributed to its rapid exchange through the nuclear pore complex.^[^ ^34^ ^]^ The fluorescence ratio of sfLHGFR_H_ in the mitochondria was also analyzed. L2HGDH, a key enzyme in l‐2‐HG catabolism, is located in the inner mitochondrial membrane and catalyzes the conversion of l‐2‐HG to 2‐KG.^[^ ^7^ ^]^ As expected, a relatively low level of l‐2‐HG was detected in the mitochondria (Figure 5d).

ME2 has previously been reported to promote l‐2‐HG production in tumor cells.^[^ ^31^ ^]^ Since ME2 is localized in the mitochondria, ME2‐mediated l‐2‐HG production was speculated to be mitochondria‐specific.^[^ ^31^ ^]^ To verify this hypothesis, ME2 was coexpressed with sfLHGFR_H_ in HEK293FT cells (Figure S10c, Supporting Information). As shown in Figure 5e, the fluorescence ratio of sfLHGFR_H_ in mitochondria increased in response to ME2 overexpression, whereas the cytosolic ratio remained unchanged, demonstrating the function of ME2 in mitochondrial l‐2‐HG synthesis.

### 2.6 Analysis of l‐2‐HG Transport Between Cytosolic and Mitochondrial Pools Using sfLHGFR_H_

**Figure 6:**
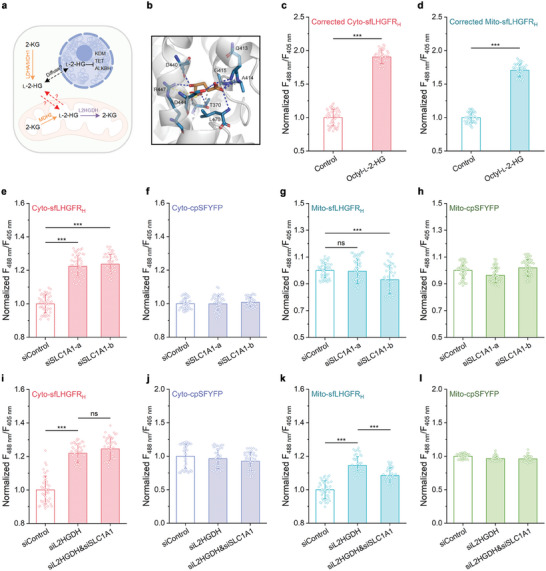
Analysis of mitochondrial l‐2‐HG transport using sfLHGFR_H_. a) Schematic representation of the mechanisms of l‐2‐HG metabolism, transport, and functions in mammalian cells. KDM, histone lysine demethylase. TET, 5‐methylcytosine hydroxylase. ALKBH, DNA repair enzyme. This figure was generated using BioRender. b) Molecular docking result of SLC1A1 with l‐2‐HG. The interacting amino acid residues were shown. c,d) Response of Cyo‐sfLHGFR_H_ c) and Mito‐sfLHGFR_H_ d) to 700 µm octyl‐l‐2‐HG. Data were corrected by cpSFYFP and normalized to the control (*n* = 49 cells). e–h) Analysis of SLC1A1 function in mitochondrial l‐2‐HG uptake. The fluorescence ratios of Cyto‐sfLHGFR_H_ e), Cyto‐cpSFYFP f), Mito‐sfLHGFR_H_ g), and Mito‐cpSFYFP h) were determined 48 h after SLC1A1 knockdown. 700 µm octyl‐l‐2‐HG was added 12 h before imaging. Data were normalized to the control condition treated with negative siRNA (*n* = 49 cells). i–l) Analysis of SLC1A1 function in mitochondrial l‐2‐HG efflux. The fluorescence ratios of Cyto‐sfLHGFR_H_ i), Cyto‐cpSFYFP j), Mito‐sfLHGFR_H_ k), and Mito‐cpSFYFP l) were determined 48 h after L2HGDH and SLC1A1 knockdown. Data were normalized to the control condition treated with negative siRNA (*n* = 42 cells). All data shown are means ± s.d. *** *p* < 0.001; ns, no significant difference (*p* ≥ 0.05) in the two‐tailed *t*‐test.

The abnormal accumulation of l‐2‐HG is closely associated with the occurrence and progression of l‐2‐hydroxyglutaric aciduria and various cancers.^[^ ^10^, ^12^, ^13^ ^]^ l‐2‐HG produced in the cytosol should be transported into the mitochondria and then degraded by L2HGDH (**Figure** **6**a). However, the mechanism underlying the transport of l‐2‐HG across the mitochondrial membrane has not yet been elucidated.^[^ ^1^ ^]^ SLC1A1 binds to l‐glutamate and mediates its influx into the mitochondria along with Na^+^.^[^ ^35^ ^]^ Considering the high structural similarity between l‐2‐HG and l‐glutamate, SLC1A1 may also participate in the transport of l‐2‐HG across the mitochondrial membrane. As shown in Figure 6b, homology modeling and molecular docking analysis revealed an interaction between SLC1A1 and l‐2‐HG. The key sites for binding of l‐2‐HG in SLC1A1 included T370, Q413, A414, G415, D440, D444, R447, and L470. SLC1A1 knockdown in HEK293FT cells increased the cytosolic l‐2‐HG levels (Figures S10d and S17, Supporting Information). No change in the fluorescence ratio of mitochondria‐localized sfLHGFR_H_ was detected, which may be due to the low basal distribution of l‐2‐HG in the mitochondria (Figure S17, Supporting Information). Then, 700 µm cell membrane‐permeable octyl‐l‐2‐HG was used to increase l‐2‐HG levels in both cytosol and mitochondria of HEK293FT cells (Figure 6c,d). As shown in Figure 6e–h, the knockdown of SLC1A1 in HEK293FT cells treated with octyl‐l‐2‐HG resulted in an increase and decrease in the fluorescence ratio of sfLHGFR_H_ localized in the cytosol and mitochondria, respectively, indicating that SLC1A1 mediated the uptake of l‐2‐HG into the mitochondrial matrix. Next, we investigated whether SLC1A1 participates in mitochondrial l‐2‐HG efflux in HEK293FT cells. As shown in Figure 6i–l and Figure S10e (Supporting Information), the fluorescence ratio of sfLHGFR_H_ in both the cytosol and mitochondria increased significantly after L2HGDH knockdown in HEK293FT cells. Co‐knockdown of SLC1A1 with L2HGDH resulted in negligible changes in cytosolic l‐2‐HG levels. The slight decrease in mitochondrial l‐2‐HG levels may be due to the disruption of mitochondrial l‐2‐HG uptake. In addition, treatment of L2HGDH knockdown cells with the SLC1A1 inhibitor dl‐*threo*‐β‐benzyloxyaspartate (dl‐TBOA) had no effect on the distribution of l‐2‐HG between different subcellular compartments (Figure S18, Supporting Information). These results suggested that SLC1A1 may not account for mitochondrial l‐2‐HG efflux in HEK293FT cells.

### 2.7 Convenient and Accurate Assay of l‐2‐HG in Human Body Fluids Using sfLHGFR_L_

**Figure 7:**
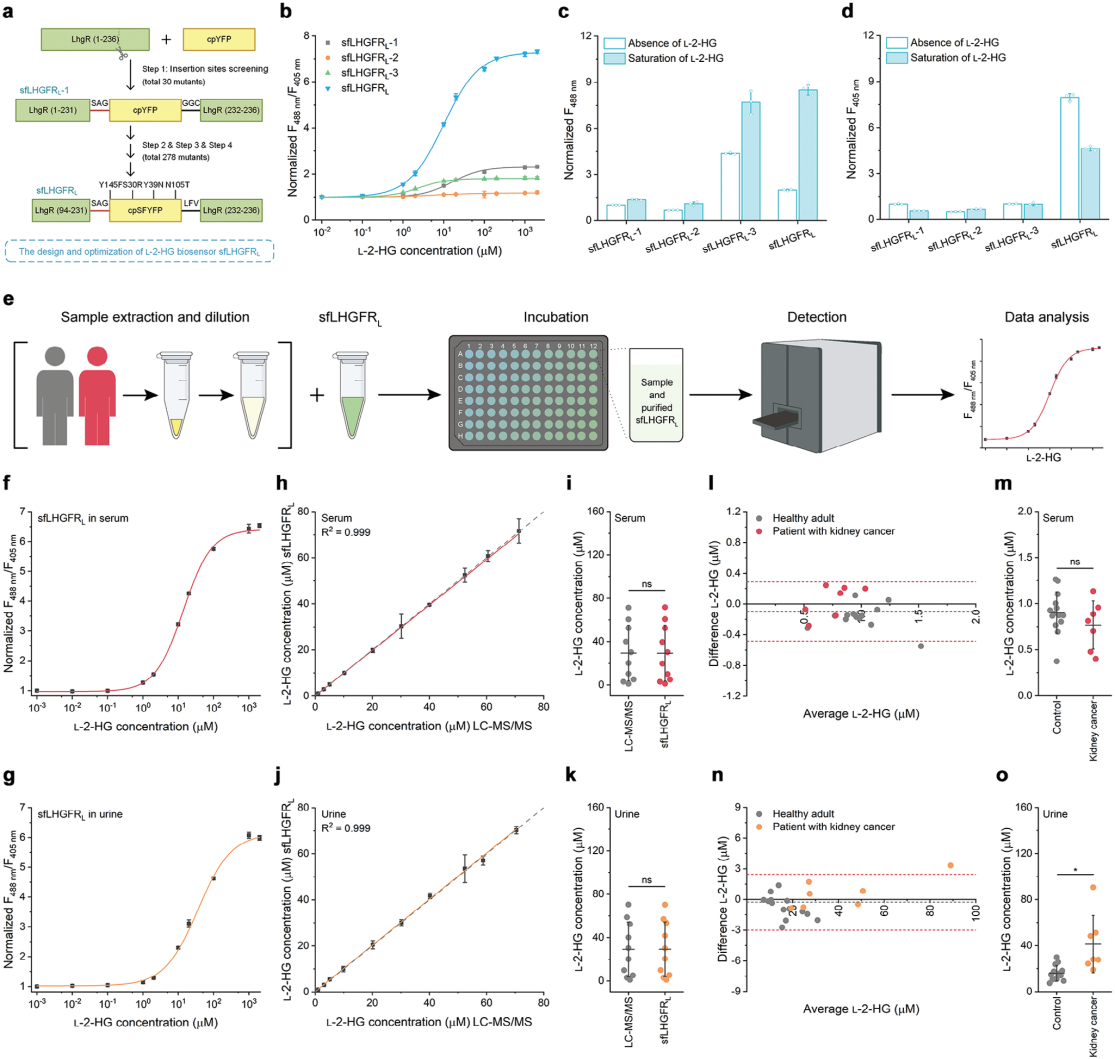
Quantification of l‐2‐HG in human body fluids using sfLHGFR_L_. a) Schematic representation of the development of sfLHGFR_L_. b) Comparison of the dose‐response curves of sfLHGFR_L_‐1, sfLHGFR_L_‐2, sfLHGFR_L_‐3, and sfLHGFR_L_ for l‐2‐HG. Data were normalized to the initial ratio. c,d) Comparison of the fluorescence intensities of sfLHGFR_L_‐1, sfLHGFR_L_‐2, sfLHGFR_L_‐3, and sfLHGFR_L_ with excitation at 488 nm c) and 405 nm d). Data were normalized to the fluorescence intensity of sfLHGFR_L_‐1 in the absence of l‐2‐HG. e) Schematic representation of the workflow for sfLHGFR_L_‐based l‐2‐HG detection in human body fluids. This figure was generated using BioRender. f,g) Dose‐response curves of sfLHGFR_L_ for increasing concentrations of l‐2‐HG in serum f) and urine g). h) Comparison between the quantitative results of l‐2‐HG in serum by sfLHGFR_L_ and LC‐MS/MS. Gray dotted line indicated a reference line with a slope of 1. i) The difference in serum l‐2‐HG concentrations measured by sfLHGFR_L_ and LC‐MS/MS. j) Comparison between the quantitative results of l‐2‐HG in urine by sfLHGFR_L_ and LC‐MS/MS. k) The difference in urine l‐2‐HG concentrations measured by sfLHGFR_L_ and LC‐MS/MS. l) Bland–Altman analysis for l‐2‐HG in serum samples from healthy adults and patients with kidney cancer measured by sfLHGFR_L_ and LC‐MS/MS. Bias (gray dotted line) and 95% limits of agreement (red dotted lines) were shown. m) Comparison of individual l‐2‐HG concentrations in serum samples measured by sfLHGFR_L_. n) Bland–Altman analysis for l‐2‐HG in urine samples from healthy adults and patients with kidney cancer measured by sfLHGFR_L_ and LC‐MS/MS. o) Comparison of individual l‐2‐HG concentrations in urine samples measured by sfLHGFR_L_. All data shown are means ± s.d. (*n* ≥ 3 independent experiments for b‐k; *n* = 14 and seven samples from healthy adults and patients with kidney cancer for l–o). * *p* < 0.05; ns, no significant difference (*p* ≥ 0.05) in the two‐tailed *t*‐test.

Quantification of l‐2‐HG in human body fluids is critical for the diagnosis and prognostic assessment of l‐2‐HG‐related diseases such as l‐2‐hydroxyglutaric aciduria and kidney cancer.^[^ ^10^, ^36^, ^37^ ^]^ The l‐2‐HG concentrations ranged from 0.5 to 84 µm in human plasma.^[^ ^10^ ^]^ Fortunately, a highly sensitive l‐2‐HG biosensor with a low detection range, sfLHGFR_231E/232L_, was obtained in the first step of biosensor construction (cpYFP insertion site screening) (Figure S2, Supporting Information). Thus, sfLHGFR_231E/232L_ was denoted as sfLHGFR_L_‐1, and then systematically optimized. The best variant of sfLHGFR_L_‐1 with high Δ*R_max_* (623.01 ± 8.52%) and high fluorescence intensity was obtained from 278 mutants and denoted as sfLHGFR_L_ (**Figure** **7**a–d; Figure S19, Supporting Information). The amino acid sequence of sfLHGFR_L_ is shown in Figure S20 (Supporting Information).

Then, the properties of sfLHGFR_L_ were characterized and similar results to sfLHGFR_H_ were obtained (Figure S21, Supporting Information). Importantly, sfLHGFR_L_ exhibited an LOD of 0.14 µm, which is much lower than the minimum reported concentration of l‐2‐HG in human serum and urine (0.5 µm) (Figure 7b; Table S1, Supporting Information). Therefore, a workflow for l‐2‐HG quantification in human body fluids was developed based on the sensitive biosensor sfLHGFR_L_. The workflow involved four steps: sample extraction and dilution; incubation with sfLHGFR_L_ in 96‐ or 384‐well plates; detection of changes in the fluorescence ratio using a fluorescence microplate reader; and data analysis (Figure 7e). l‐2‐HG at gradient concentrations were spiked into healthy human serum and urine samples and then quantified using sfLHGFR_L_ and LC‐MS/MS. As shown in Figure 7f–g and Figure S22 (Supporting Information), sfLHGFR_L_ responded to the gradients of l‐2‐HG in human body fluids in a dose‐dependent manner. The quantitative results of sfLHGFR_L_ for l‐2‐HG in these samples were in close agreement with those of LC‐MS/MS, the current standard technology for clinical l‐2‐HG assessment (Figure 7h–k). The applicability of sfLHGFR_L_ was further proven by analyzing authentic serum and urine samples from 14 healthy adults and seven patients with kidney cancer (Table S2, Supporting Information). As shown in Figure 7l,m, l‐2‐HG quantitation in serum samples using sfLHGFR_L_ yielded results that were consistent with those obtained using LC‐MS/MS. The concentration of l‐2‐HG in the serum samples ranged from 0.5 to 1.5 µm. No significant differences in serum l‐2‐HG concentrations were observed between the two population groups. The quantitation of l‐2‐HG in urine samples using sfLHGFR_L_ also agreed closely with the LC‐MS/MS results (Figure 7n). Importantly, urine l‐2‐HG concentrations in patients with kidney cancer were ≈2.58‐fold higher than those in healthy adults, suggesting that urine l‐2‐HG has the potential to be a clinically valuable biomarker for the diagnosis and treatment of kidney cancer (Figure 7o).

## 3 Discussion

Biosensors for practical applications should have high responsiveness, appropriate affinity, high specificity, rapid response, and intense fluorescence.^[^ ^38^ ^]^ Two FRET‐based l‐2‐HG biosensors, LHGFR_0N3C_ and LHGFR_0N7C_, were recently developed for l‐2‐HG assays.^[^ ^22^ ^]^ However, the relatively low response magnitude (< 61%) and high affinity of LHGFR_0N3C_ and LHGFR_0N7C_ limited their applicability in detecting intracellular l‐2‐HG (Table S1, Supporting Information). In this study, cpFP‐based l‐2‐HG biosensors were constructed using the l‐2‐HG‐specific transcriptional regulator LhgR and cpYFP. An ultrasensitive l‐2‐HG biosensor, sfLHGFR_H_, was obtained using a four‐step systematic optimization workflow. sfLHGFR_H_ exhibited a high response magnitude (909.23 ± 16.19%), supported ratiometric readout, and possessed good specificity and high stability. The disadvantages of poor folding and dim fluorescence in traditional cpFP‐based biosensors have been addressed by substituting four superfolder sites in cpYFP of sfLHGFR_H_.^[^ ^39^ ^]^ Importantly, the sfLHGFR_H_ had an appropriate affinity (*K_d_* = 181.77 ± 17.41 µm) toward l‐2‐HG, making it suitable for real‐time imaging of l‐2‐HG in vivo. The hypoxia‐induced intracellular l‐2‐HG accumulation and the roles of L2HGDH as well as LDHA and MDH2 in l‐2‐HG catabolism and anabolism were monitored using sfLHGFR_H_ (Figure 3g–j). Thus, sfLHGFR_H_ allowed for the previously unavailable spatiotemporal characterization of l‐2‐HG dynamics at the single‐cell level (Figure 3), which will help to provide novel insights into the heterogeneity of l‐2‐HG metabolism in single cells.

l‐2‐HG is an important metabolite involved in various physiological processes in living organisms.^[^ ^2^, ^3^, ^4^, ^5^, ^6^, ^40^, ^41^, ^42^ ^]^ Macrophages are crucial effector cells of the innate immunity.^[^ ^26^, ^27^, ^28^ ^]^ l‐2‐HG has recently been found to accumulate in LPS‐induced M1 macrophages.^[^ ^29^, ^30^ ^]^ However, the distribution, accumulation mechanism, and functions of l‐2‐HG in differently polarized macrophages remain unknown. The sfLHGFR_H_ was introduced in RAW 264.7 macrophages (Figure 4a–c), and the accumulation of l‐2‐HG in both M1 and M2 macrophages was confirmed (Figure 4d). In M1 macrophages, the upregulation of LDHA and downregulation of L2HGDH contributed to the accumulation of l‐2‐HG (Figure 4j). Both l‐2‐HG and succinate can stabilize HIF‐1α, a key regulator involved in M1 macrophage polarization,^[^ ^27^ ^]^ by inhibiting prolyl hydroxylase.^[^ ^1^, ^5^, ^8^ ^]^ However, while the succinate‐HIF‐1α axis only regulates IL‐1β expression,^[^ ^27^ ^]^ l‐2‐HG also promotes IL‐6 and TNFα expression in M1 macrophages (Figure S12, Supporting Information), suggesting that the proinflammatory effects of l‐2‐HG are not identical to that of succinate. In M2 macrophages, the upregulation of LDHA mediated the l‐2‐HG accumulation, which inhibited the expression of Mrc1 and promoted the expression of Arg1 and TGF‐β (Figure 4j; Figure S13, Supporting Information). Multiple isoenzymes of LDH and MDH exist in mammalian cells. Differences in their spatiotemporal expression and catalytic properties may account for the variable l‐2‐HG metabolism between different cell types. The convenient and efficient identification of l‐2‐HG metabolic mechanism in specific cells can be achieved by integrating sfLHGFR_H_ into different cells. In addition, some TCA cycle intermediates like succinate and fumarate functioning in macrophage activation have been characterized as “immunometabolites” and key therapeutic targets.^[^ ^43^, ^44^ ^]^ Considering the immunomodulatory effects of l‐2‐HG on macrophages, targeting l‐2‐HG metabolism may also be a promising therapeutic strategy that can be achieved by modulating the activities of LDHA and L2HGDH.

The metabolism and function of l‐2‐HG are highly compartmentalized in mammalian cells.^[^ ^1^ ^]^ l‐2‐HG affects epigenetic modifications by inhibiting various histone lysine demethylases and 5‐methylcytosine hydroxylases in the nucleus.^[^ ^1^, ^5^, ^8^, ^9^, ^11^ ^]^ However, the presence of l‐2‐HG in the nucleus has not been confirmed. In this study, we fused different localization signals with sfLHGFR_H_ and demonstrated its application in the in situ detection of l‐2‐HG fluctuations in the cytosol, mitochondria, and nucleus (Figure 5). Then, the presence of l‐2‐HG in the nucleus was confirmed using sfLHGFR_H_. The concentration of l‐2‐HG was almost identical to that in the cytosol (Figure 5d). Metabolites required for epigenetic regulation (such as 2‐KG, fumarate, succinate, and 2‐HG) are generally accepted to diffuse from the cytosol to the nucleus through the nuclear pore complex.^[^ ^34^ ^]^ A recent study of metabolic subnetworks in the mammalian nucleus revealed the distribution of certain key enzymes of central metabolism in the nucleus.^[^ ^34^, ^45^, ^46^ ^]^ In addition to diffusion from the cytosol, nuclear l‐2‐HG may be generated in situ within the nucleus. The nucleus‐localized sfLHGFR_H_ could be applied in the future identification of the metabolic origins of l‐2‐HG in the nucleus.

l‐2‐HG is produced in the cytosol (mediated by LDHA and MDH1) and mitochondria (mediated by MDH2) in most cell lines.^[^ ^3^, ^4^ ^]^ L2HGDH is a key enzyme involved in l‐2‐HG degradation and is located in the inner mitochondrial membrane.^[^ ^7^ ^]^ In this study, SLC1A1, a mitochondrial l‐glutamate transporter,^[^ ^35^ ^]^ was found to be involved in the mitochondrial l‐2‐HG uptake of HEK293FT cells using sfLHGFR_H_ (Figure 6e–h). Molecular docking analysis revealed that l‐2‐HG was anchored to the binding pocket of SLC1A1, which is composed of residues T370, Q413, A414, G415, D440, D444, R447, and L470 (Figure 6b). l‐2‐HG is produced via cytosolic LDHA‐ and mitochondrial MDH2‐mediated 2‐KG reduction in HEK293FT cells.^[^ ^3^ ^]^ L2HGDH knockdown caused a greater increase in l‐2‐HG levels in the cytosol than in the mitochondria, implying the possible release of mitochondrial l‐2‐HG into the cytosol of HEK293FT cells. Interestingly, additional knockdown of SLC1A1 decreased the mitochondrial l‐2‐HG levels. This phenomenon may be due to the disruption of SLC1A1‐mediated mitochondrial l‐2‐HG uptake, indicating that SLC1A1 may not account for mitochondrial l‐2‐HG efflux (Figure 6i–l; Figure S18, Supporting Information). Further investigation is required to elucidate the mechanisms of mitochondrial l‐2‐HG release, especially in some cancer cells where l‐2‐HG is primarily produced in the mitochondria (e.g., renal cell carcinoma cells, in which MDH2 is primarily responsible for l‐2‐HG synthesis, whereas the inhibitory effect of l‐2‐HG on 2‐KG‐dependent dioxygenase occurs in the cytosol and nucleus).^[^ ^47^ ^]^ Notably, SLC1A1 is principally expressed in the neurons, kidneys, and small intestine,^[^ ^48^ ^]^ and other transporters involved in the uptake and efflux of l‐2‐HG might be identified via sfLHGFR_H_‐supported genome‐wide CRISPR screening in various cell types.^[^ ^49^ ^]^

l‐2‐HG is also a critical biomarker for the diagnosis and prognosis of l‐2‐HG‐related diseases.^[^ ^10^, ^36^, ^37^ ^]^ Based on the design concept of sfLHGFR_H_, a highly sensitive l‐2‐HG biosensor, sfLHGFR_L_, with a high response magnitude of 623.01 ± 8.52% and a low *K_d_* of 9.94 ± 0.46 µm was also developed. The insertion site of cpSFYFP in sfLHGFR_L_ is located in the loop region after the last α‐helix of LhgR (94L‐231E/232L‐236D), while the insertion site of cpSFYFP in sfLHGFR_H_ is located within the ligand binding domain of the LhgR (94L‐137D/138F‐236D). This may interfere with l‐2‐HG binding, and thus make sfLHGFR_H_ exhibit a higher *K_d_* value. Compared to previous mass spectrometry or enzyme‐based assays, the as‐designed biosensor is convenient to handle and consists of only one assay component (Table S1, Supporting Information).^[^ ^50^, ^51^ ^]^ In addition, the lower LOD of sfLHGFR_L_ (0.14 µm) made it more applicable for l‐2‐HG detection in clinical samples than LHGFR_0N3C_ (LOD of 4.34 µm) and LHGFR_0N7C_ (LOD of 0.70 µm) (Table S1, Supporting Information).^[^ ^22^ ^]^ Quantitative results obtained using sfLHGFR_L_ were consistent with those obtained using LC‐MS/MS. Analysis of serum and urine l‐2‐HG levels in 14 healthy adults and seven patients with kidney cancer using sfLHGFR_L_ revealed that l‐2‐HG significantly accumulated in the urine of patients with kidney cancer (Figure 7l–o). Further quantitative analysis of l‐2‐HG in large‐scale clinical samples is required to determine the correlation between kidney cancer progression and urine l‐2‐HG accumulation, which may be achieved by the sfLHGFR_L_‐supported high‐throughput in vitro l‐2‐HG assays.

In summary, two ultrasensitive l‐2‐HG biosensors, sfLHGFR_H_ and sfLHGFR_L_, were achieved with superior performance in tracking l‐2‐HG fluctuations in vivo and in vitro. Both sfLHGFR_H_ and sfLHGFR_L_ showed high response magnitude, good specificity, and high stability. Convenient, accurate, and highly spatiotemporally resolved assays for l‐2‐HG levels in different cell types, subcellular compartments, and biological samples were performed using sfLHGFR_H_ and sfLHGFR_L_. The lower abundance of the mitochondrial l‐2‐HG pool and the role of SLC1A1 in mitochondrial l‐2‐HG uptake were elucidated using sfLHGFR_H_. The abnormal accumulation of l‐2‐HG in the urine of patients with kidney cancer was discovered using sfLHGFR_L_. These two biosensors may serve as valuable tools for illuminating l‐2‐HG metabolism and achieving point‐of‐care diagnosis and monitoring of l‐2‐HG‐related diseases.

## 4 Experimental Section

### Bacterial Strains and Culture Conditions

The bacterial strains used in this study are listed in Table S3 (Supporing Information). *Escherichia coli* and its derivatives were cultured in Luria–Bertani (LB) broth at 37 °C and 180 rpm. Antibiotics were used at the following concentrations: tetracycline at 30 µg mL^−1^; spectinomycin, at 50 µg mL^−1^; ampicillin at 100 µg mL^−1^; kanamycin at 50 µg mL^−1^.

### Cell Lines and Culture Conditions

HEK293FT cells were maintained in high‐glucose Dulbecco's modified eagle medium (DMEM) (ThermoFisher, USA) supplemented with 10% (vol/vol) fetal bovine serum (FBS) (Biological Industries, Israel), 100 units mL^−1^ penicillin, and 100 µg mL^−1^ streptomycin (ThermoFisher, USA) at 37 °C in humidified air containing 5% CO_2_. For hypoxic conditions, HEK293FT cells were incubated in a compact O_2_ and CO_2_ subchamber controller (ProOx C21, BioSpherix, USA) at 2% O_2_ equilibrated with N_2_.

RAW 264.7 murine macrophages (obtained from China Center for Type Culture Collection, China) were maintained in high‐glucose DMEM supplemented with 10% (vol/vol) FBS, 1 mm sodium pyruvate (ThermoFisher, USA), 100 units mL^−1^ penicillin, and 100 µg mL^−1^ streptomycin at 37 °C in humidified air containing 5% CO_2_. The medium was changed daily to maintain cell viability. To induce M1 macrophage polarization, the cells were treated with 1 µg mL^−1^ LPS (Sigma‐Aldrich, USA) and 20 ng mL^−1^ IFN‐γ (Absin Bioscience, China). To induce M2 macrophage polarization, the cells were treated with 20 ng mL^−1^ IL‐4 (Absin Bioscience, China).

### Construction and Optimization of sfLHGFR_H_ and sfLHGFR_L_—Insertion Sites Screening

All the plasmids and primers used in this study are listed in Tables S3 and S4 (Supporting Information), respectively. The l‐2‐HG catabolic gene cluster F2‐*lhgR*‐F1‐*lhgO* of *P. putida* W619 was synthesized by General Biology Co., Ltd (China).^[^ ^22^ ^]^ The upstream fragment of LhgR, the fragment of cpYFP, and the downstream fragment of LhgR were amplified separately and combined through overlap PCR. The product was then cloned into pETDuet‐1 plasmid using the BamHI and HindIII restriction sites and T5 exonuclease DNA assembly (TEDA) method.^[^ ^52^ ^]^ The recombinant plasmid was transferred into *E. coli* BL21(DE3) for further protein expression and purification.

### Construction and Optimization of sfLHGFR_H_ and sfLHGFR_L_—Linker and DNA‐Binding Domain Truncation

To remove the DNA‐binding domain of LhgR, the DNA fragments of biosensor variants were first amplified by primer pairs D2‐LhgR‐F/D2‐LhgR‐R (for the construction of sfLHGFR_H_‐2 and its variants) or D2‐LhgR‐F’/D2‐LhgR‐R’ (for the construction of sfLHGFR_L_‐2 and its variants), and then cloned into pETDuet‐1 plasmid using the BamHI and HindIII restriction sites. To truncate the linker between cpYFP and LhgR, the DNA fragments of cpYFP with truncated N‐ and C‐terminal linker were amplified and ligated to the plasmid backbone acquired by inverse PCR amplification.

### Construction and Optimization of sfLHGFR_H_ and sfLHGFR_L_—Fluorescent Protein Replacement and Superfolder Sites Substitution

To replace the fluorescent protein in sfLHGFR_H_‐2, the DNA fragments of different fluorescent proteins were synthesized by General Biology Co., Ltd (China), amplified by respective primer pairs, and ligated to the plasmid backbone. To introduce four superfolder sites (S30R, Y39N, N105T, and Y145F) in cpYFP (the obtained fluorescent protein was denoted as cpSFYFP), cpYFP was amplified into two parts by primer pairs cpSFYFP‐F1/cpSFYFP‐R1 and cpSFYFP‐F2/cpSFYFP‐R2, respectively. The DNA fragments of cpSFYFP were then acquired by linking the products through overlap PCR and ligated to the plasmid backbone.

### Construction and Optimization of sfLHGFR_H_ and sfLHGFR_L_—Random Linker Mutagenesis

The DNA fragments of cpSFYFP with random linker were amplified by degenerate primers (for example, primer pairs 0N‐mutation and 0C‐mutation), ligated to the plasmid backbone, and then transferred into *E. coli* BL21(DE3). Bright colonies expressing the mutant biosensors were screened from LB plates containing 100 µg mL^−1^ ampicillin, cultured in 5 mL LB medium in 50 mL centrifuge tubes, and then the bacterial cells were induced with 1 mm isopropyl‐β‐d‐1‐thiogalactopyranoside (IPTG) for 12 h at 23 °C. Bacterial cells were collected by centrifugation at 6000 × g for 10 min at 4 °C, resuspended in 48‐well deep well plates with 100 mm HEPES buffer containing 100 mm KCl (pH 7.4), and then lysed by multi‐channel ultrasonic breaker (SCIENTZ‐48TD, Scientz Biotechnology, China) in the presence of 1 mm phenylmethylsulfonyl fluoride (PMSF). The supernatants were acquired by centrifugation at 13 000 × g for 5 min at 4 °C, followed by the high‐throughput analysis of the fluorescence response to l‐2‐HG by a microplate reader (EnSight, PerkinElmer, USA). Mutants with high response magnitude and bright fluorescence were screened and sequenced.

### Protein Expression and Purification

*E. coli* BL21(DE3) strains harboring pETDuet‐1‐based plasmids expressing various recombinant proteins, including sfLHGFR_H_, sfLHGFR_L_, as well as different biosensor variants, were cultivated in 500 mL LB medium at 37 °C and 180 rpm to reach an optical density at 600 nm (OD_600_) of 0.6, and treated with 1 mm IPTG for 12 h at 23 °C and 160 rpm. Bacterial cells were collected by centrifugation at 6000 × g for 10 min at 4 °C, washed by phosphate‐buffered saline (PBS), suspended in buffer A (20 mm sodium phosphate, 500 mm NaCl, and 20 mm imidazole, pH 7.4), and lysed via sonication on ice in the presence of 1 mm PMSF. The supernatants were obtained by centrifugation at 13 000 × g for 40 min at 4 °C, loaded onto a 5 mL HisTrap HP column (GE Healthcare, USA) equilibrated with buffer A, and then eluted with a gradient of buffer B (20 mm sodium phosphate, 500 mm NaCl, and 500 mm imidazole, pH 7.4) at a flow rate of 5 mL min^−1^. The purified protein was then exchanged into 100 mm HEPES buffer containing 100 mm KCl (pH 7.4), analyzed by 13% sodium dodecyl sulfate‐polyacrylamide gel electrophoresis (SDS‐PAGE), and quantified by the Bradford protein assay kit (Sangon, China). cpSFYFP protein encoded in the pET28a^(+)^ plasmid was expressed and purified by the same procedure.

### Characterization of sfLHGFR In Vitro—Dose‐Response Curve Determination

Purified l‐2‐HG biosensors sfLHGFR_H_ and sfLHGFR_L_ were diluted to a final concentration of 0.2 µm with 100 mm HEPES buffer containing 100 mm KCl (pH 7.4), and titrated with increasing concentrations of l‐2‐HG (prepared in the same buffer) in a black 96‐well plate (PerkinElmer, USA) at a volume ratio of 3:1 (total 100 µL per well). The fluorescence intensities were measured immediately by an EnSight microplate reader using 405 nm or 488 nm excitation and 528 nm emission. The fluorescence emission ratio of 528 nm with 488 nm and 405 nm excitation (F_488 nm_/F_405 nm_) was plotted against the l‐2‐HG concentration and fitted by OriginPro 2019 software (OriginLab, USA) according to the following formula:

(1) $$R=R_{\max}+\frac{R_{\min}-R_{\max}}{1+{\left(\left[L-2-\mathrm{HG}\right]/K_{d}\right)}^{p}}$$

where *R*, *R_min_*, and *R_max_* represented the experimental F_488 nm_/F_405 nm_, the minimum F_488 nm_/F_405 nm_, and the maximum F_488 nm_/F_405 nm_ of the l‐2‐HG biosensors, respectively. The [l‐2‐HG], *K_d_*, and *p* indicated the concentration of l‐2‐HG, apparent dissociation constant, and Hill slope, respectively. The response magnitudes (maximum ratio changes, Δ*R_max_*) were calculated as Δ*R_max_* = (*R_max_* − *R_min_*)/*R_min_*.

### Characterization of sfLHGFR In Vitro—Spectra Properties Analysis

Fluorescence spectroscopy of sfLHGFR_H_ and sfLHGFR_L_ was analyzed on an EnSight microplate reader using 0.2 µm purified biosensors and 1 mm l‐2‐HG. For excitation spectra, emission was measured at 530 nm with excitation from 380 to 514 nm in steps of 2 nm. For emission spectra, emission was measured from 503 to 649 nm in steps of 2 nm with excitation at 405 nm or 488 nm.

### Characterization of sfLHGFR In Vitro—Specificity Analysis

Purified l‐2‐HG biosensors sfLHGFR_H_ and sfLHGFR_L_ were mixed with different l‐2‐HG analogs, including octyl‐l‐2‐HG, d‐lactate, l‐lactate, d‐2‐HG, and a series of intermediates of the TCA cycle and the l‐lysine catabolism, respectively, and the fluorescence ratios were measured immediately.

### Characterization of sfLHGFR In Vitro—Anti‐Interference Analysis

Purified l‐2‐HG biosensors sfLHGFR_H_ and sfLHGFR_L_ were premixed with different l‐2‐HG analogs, including octyl‐l‐2‐HG, d‐lactate, l‐lactate, d‐2‐HG, and a series of intermediates of the TCA cycle and the l‐lysine catabolism, respectively. l‐2‐HG was then added to the mixed system, and the fluorescence ratios were measured immediately.

### Characterization of sfLHGFR In Vitro—Reversibility Analysis

Purified l‐2‐HG biosensors sfLHGFR_H_ and sfLHGFR_L_ were titrated with different concentrations of l‐2‐HG (0, 10, 100 µm for sfLHGFR_H_; 0, 1, 10 µm for sfLHGFR_L_), and then the fluorescence ratios were recorded. The removal of l‐2‐HG was realized by centrifugation at 3800 × g for 20 min at 4 °C using 10‐kDa ultrafiltration centrifuge tubes, and then the fluorescence ratios of the l‐2‐HG biosensors were recorded again.

### Characterization of sfLHGFR In Vitro—Temperature‐Stability Analysis

Temperature‐stability analysis of sfLHGFR_H_ and sfLHGFR_L_ was performed by determining the fluorescence ratios to l‐2‐HG (1 and 1000 µm for sfLHGFR_H_; 1 and 100 µm for sfLHGFR_L_) at the gradient temperature (25–45 °C).

### Characterization of sfLHGFR In Vitro—pH‐Stability Analysis

pH‐stability analysis of sfLHGFR_H_ and sfLHGFR_L_ was performed by determining the dose‐response curves to increasing concentrations of l‐2‐HG at the gradient pH value (pH 6.6–8.2). The correction of sfLHGFR_H_ and sfLHGFR_L_ by cpSFYFP was realized by dividing the fluorescence ratio of sfLHGFR_H_ or sfLHGFR_L_ over the fluorescence ratio of cpSFYFP [(F_488 nm_/F_405 nm_)_sfLHGFRH_/(F_488 nm_/F_405 nm_)_cpSFYFP_ or (F_488 nm_/F_405 nm_)_sfLHGFRL_/(F_488 nm_/F_405 nm_)_cpSFYFP_].^[^ ^18^, ^19^ ^]^

### Expression of sfLHGFR_H_ in HEK293FT Cells

The codon‐optimized sfLHGFR_H_‐2 sequence was synthesized and cloned into the mammalian expression plasmid pcDNA3.1^(+)^ behind a Kozak sequence by General Biology Co., Ltd (China). The mammalian expression plasmid for sfLHGFR_H_‐3 was constructed consistent with the construction of the *E. coli* BL21(DE3) expression plasmid as described above. sfLHGFR_H_ and other mutants were constructed by amplifying the DNA fragments of cpSFYFP with different linkers by respective primers and ligating to the plasmid backbone acquired by inverse PCR amplification.

Transient expression of l‐2‐HG biosensors was performed with Lipofectamine 3000 (ThermoFisher, USA) or FuGENE HD (Promega, USA) according to the manufacturer's instructions. Briefly, cells were cultured in 35‐mm diameter glass‐bottom dishes precoated with poly‐l‐lysine (Sigma‐Aldrich, USA), grown to a confluency of 70−90%, and replaced with fresh complete medium (for Lipofectamine 3000) or antibiotic‐free medium (for FuGENE HD) 2 h before transfection. 4.6 µL Lipofectamine 3000 reagent, 4.6 µL p3000 reagent, and 3 µg pcDNA3.1^(+)^ plasmid encoding sfLHGFR_H_‐2, sfLHGFR_H_‐3, sfLHGFR_H_, or other mutants were mixed in Opti‐MEM Reduced Serum Medium (ThermoFisher, USA), incubated for 15 min at room temperature, and then added into the cultures. Alternatively, 6 µL FuGENE HD reagent and 2 µg plasmid were mixed, incubated, and then added to the cultures. Imaging was typically carried out 24−48 h after transfection.

### Imaging of sfLHGFR_H_ in HEK293FT Cells

Imaging of sfLHGFR_H_ in HEK293FT cells was carried out by using a Zeiss 900 confocal microscope equipped with 20×/0.8 and 40×/0.95 objective lens, 405 and 488 nm lasers, and a full‐spectrum fluorescence detector. Images were acquired with 405 and 488 nm excitation, 503–617 nm emission range, 800 gain, 1024 × 1024 frame size, and 8‐bit depth. All data from the same experiment were captured with the same imaging parameters and laser power. In particular, the 488 and 405 nm lasers were used at 0.1% power and 0.4% power, respectively, for comparing the expression and brightness of sfLHGFR_H_‐2, sfLHGFR_H_‐3, and other mutants in HEK293FT cells. Images were captured randomly by ZEN3.1 software, but cells with particularly strong or low fluorescence were excluded for analysis. Fluorescence ratio images (F_488 nm_/F_405 nm_) were generated through pixel‐by‐pixel calculations using ImageJ software.^[^ ^53^ ^]^ In order to analyze the real‐time response of the l‐2‐HG biosensor to l‐2‐HG or its analogs including d‐lactate, l‐lactate, and d‐2‐HG, HEK293FT cells expressing sfLHGFR were first washed twice with HHBSS buffer (1× Hank's balanced salt solution supplemented with 20 mm HEPES, all purchased from ThermoFisher, USA) to minimize background, and the fluorescence ratio changes before and after the addition of 80 µm digitonin and different compounds were imaged at an interval of 30 s. The background fluorescence without the expression of sfLHGFR was subtracted.

### Knockdown of Genes by siRNA

Silencer Select siRNAs targeting L2HGDH, LDHA, MDH2, or SLC1A1 (ThermoFisher, USA) were used for the knockdown of genes in HEK293FT cells. Negative control siRNA with no effect on cell viability (ThermoFisher, USA) was used as a control. Transfection of siRNA and sfLHGFR_H_ was performed with Lipofectamine 3000 according to the manufacturer's instructions. For the biological function analysis of L2HGDH, siRNA targeting L2HGDH or negative control siRNA and pcDNA3.1^(+)^ plasmid encoding sfLHGFR_H_ or cpSFYFP were co‐transfected into HEK293FT cells, and the fluorescence ratio was determined 48, 72, and 96 h after transfection, respectively. For the biological function analysis of LDHA and MDH2, siRNA targeting LDHA, siRNA targeting MDH2, or negative control siRNA and pcDNA3.1^(+)^ plasmid encoding sfLHGFR_H_ or cpSFYFP were co‐transfected into HEK293FT cells. The transfected cells were first cultured under normoxic conditions for 24 h, followed by 24 h under hypoxic conditions in the presence of 20 mm KMV or 5 mm DMαKG. Fluorescence ratios of sfLHGFR_H_ were subsequently measured and corrected by cpSFYFP. For the biological function analysis of SLC1A1, HEK293FT cells co‐transfected with different subcellular localizations of sfLHGFR_H_ or cpSFYFP and siRNA targeting SLC1A1 were cultured for 48 h, after which the fluorescence ratios were measured. In addition, HEK293FT cells co‐transfected with different subcellular localizations of sfLHGFR_H_ or cpSFYFP, siRNA targeting L2HGDH, and siRNA targeting SLC1A1 were cultured for 48 h, after which the fluorescence ratios were measured.

### Imaging of sfLHGFR_H_ in Macrophages

The expression of sfLHGFR_H_ in macrophages was consistent with the expression in HEK293FT cells as described above. In order to analyze the real‐time response of sfLHGFR_H_ in macrophages to l‐2‐HG, macrophages were plated in 35‐mm diameter glass‐bottom dishes without poly‐l‐lysine precoated in appropriate numbers to reach a confluency of 70−90% after 12 h, followed by transient expression of sfLHGFR_H_. The fluorescence ratio changes of 80 µm digitonin‐pretreated macrophages expressing sfLHGFR_H_ were analyzed in real‐time before and after the addition of 1 mm l‐2‐HG. In order to analyze the difference in l‐2‐HG concentrations between M0, M1, and M2 macrophages, macrophages were treated with LPS (1 µg mL^−1^) + IFN‐γ (20 ng mL^−1^) or IL‐4 (20 ng mL^−1^) 2 h after transfection with sfLHGFR_H_, and the fluorescence ratios of sfLHGFR_H_ were determined 24 h later. In order to analyze the biological functions of LDHA and MDH2 in l‐2‐HG metabolism in macrophages, macrophages were treated with LPS (1 µg mL^−1^) + IFN‐γ (20 ng mL^−1^) or IL‐4 (20 ng mL^−1^) 2 h after transfection with sfLHGFR_H_, at the same time, 85 µm LDHA inhibitor GSK2837808A (MedChemExpress, USA), 65 µm MDH2 inhibitor LW6 (MedChemExpress, USA), or an equal volume of DMSO were added into the cultures, respectively, and the fluorescence ratios of sfLHGFR_H_ were determined 24 h later. The background fluorescence without the expression of sfLHGFR_H_ was subtracted.

### RNA Isolation and Quantitative PCR

Total RNA from HEK293FT cells or from M0, M1, and M2 macrophages cultured in the presence of DMSO or 500 µm octyl‐l‐2‐HG (J&W Pharmlab, China) was extracted using TRIzol reagent (Thermo Scientific, USA) according to the manufacturer's instructions, quantified using a NanoDrop ND‐1000 (Thermo Scientific, USA), and checked using agarose gel electrophoresis. Total cDNA was synthesized with 1.5 µg RNA in a 24 µL reaction system using HiScript II Q RT SuperMix (Vazyme, China). Quantitative PCR (qPCR) was performed using appropriately diluted cDNA and AceQ qPCR SYBR Green Master Mix (Vazyme, China). The cycle threshold value (Ct) was determined using a quantitative real‐time PCR system (Lightcycler 480, Roche, Switzerland). The expression levels of the genes are normalized to the expression of GAPDH gene by using a 2^−ΔΔCt^ method. All primers for qPCR analysis used in this study are listed in Table S4 (Supporting Information).

### Macrophage Metabolite Extraction and l‐2‐HG Determination Using LC‐MS/MS

M0, M1, and M2 macrophages cultured in the absence or presence of 85 µm GSK2837808A, 65 µm LW6, or an equal volume of DMSO were washed three times with ice‐cold PBS. Then, the cells were treated with an ice‐cold extraction solution containing 50% methanol, 30% acetonitrile, and 20% distilled water (500 µL per 1 × 10^6^ cells), incubated on ice for 15 min, and collected using a cell scraper. Cell suspensions were shaken vigorously for 15 min at 4 °C, and then lysed through two freeze‐thaw cycles at −20 °C/37 °C. Proteins and cell debris were removed by centrifugation at 13 000 × g for 10 min at 4 °C. The supernatants were completely dried in a vacuum centrifuge (45 °C) (Concentrator plus, Eppendorf, Germany), resuspended in an appropriate amount of distilled water, and stored at −80 °C until further analysis.

LC‐MS/MS determination of l‐2‐HG in cell extracts was performed by a high‐performance liquid chromatography‐triple quadrupole mass spectrometry (SCIEX Triple Quad^TM^ 5500+ System – QTRAP, SCIEX, USA) equipped with a Chirobiotic R column (250 mm length × 4.6 mm I.D., 5 mm silica gel particles bonded to the macrocyclic glycopeptide ristocetin A) (Supelco Analytical, USA) in negative ion mode. A stable isotope labeled d,l‐2‐hydroxyglutarate disodium salt (2,3,3‐D_3_‐2‐HG) at the concentration of 10 µm was added to the samples as an internal standard. The mobile phase consisted of a mixture of 95% A (0.1% triethylamine adjusted to pH 4.5 with acetic acid) and 5% B (methanol). The flow rate was 0.5 mL min^−1^ and the total analysis time was 15 min. The precursor ions for 2‐HG and D_3_‐2‐HG were 147.0 and 150.0, respectively, and the product ions for 2‐HG and D_3_‐2‐HG were 129.0 and 132.0, respectively. Absolute quantification of l‐2‐HG was performed using a standard curve, which was determined for each experiment. Cells were counted to determine viable cell numbers, and intracellular concentrations of l‐2‐HG were corrected for cell number.

### Subcellular Localization of sfLHGFR_H_

For the cytosolic expression of sfLHGFR_H_, two repeats of the MAPKK nuclear export signal (MALQKKLEELELDEQQRKRLEDL)_2_ were fused to the N‐terminus of sfLHGFR_H_. For the mitochondrial expression of sfLHGFR_H_, one repeat of the mitochondrial localization signal (MLSLRQSIRFFKPATRTLCSSRYLL) was fused to the N‐terminus of sfLHGFR_H_. For the nuclear expression of sfLHGFR_H_, three repeats of the SV40 large T antigen nuclear localization signal (DPKKKRKV)_3_ were fused to the C‐terminus of sfLHGFR_H_. The expression of sfLHGFR_H_ with different subcellular compartment localization sequences in HEK293FT cells was consistent with the expression of sfLHGFR_H_ as described above. The subcellular localization analysis of sfLHGFR_H_ was performed 24 h after transfection. HEK293FT cells expressing the differentially localized sfLHGFR_H_ were incubated with 5 µg mL^−1^ Hoechst (Thermo Scientific, USA) or 200 nm Mitotracker Red (Beyotime Technology, China) in PBS at 37 °C for 15 min, washed three times, and then imaged by using a Nikon Ti‐E fluorescence microscope (Nikon Corporation, Japan).

### Homology Modeling and Molecular Docking of SLC1A1

Homology modeling and molecular docking of SLC1A1 were performed by Alphafold2 and Schrodinger Glide software, respectively. The docking conformation and the hydrogen bonding and hydrophobic interactions between SLC1A1 and l‐2‐HG as well as the key amino acid residues involved were visualized and analyzed by PyMOL software (DeLano Scientific LLC, USA) and PLIP (https://plip‐tool.biotec.tu‐dresden.de/plip‐web). The homology modeling of SLC1A1 was constructed based on the reported structure of its homologous protein SLC1A5 (alanine serine cysteine transporter 2, PDB: 6GCT).^[^ ^35^, ^54^ ^]^ For the molecular docking analysis of SLC1A1 with l‐2‐HG, the InducedFit module in Schrodinger Glide software was used, and the other parameters were default settings.

### Determination of l‐2‐HG in Human Body Fluids Using LC‐MS/MS

The serum samples from healthy adults and patients with kidney cancer were prepared by collecting venous blood with coagulation‐promoting tubes, standing at room temperature for 2 h, and then centrifuging at 2000 × g for 10 min at 4 °C. The urine samples were collected directly into centrifuge tubes. Prepared serum and urine samples were stored at −80 °C until further analysis.

For the determination of l‐2‐HG in human body fluids using LC‐MS/MS, the serum samples were mixed with an equal volume of methanol, vortexed for 2 min, centrifuged at 13 000 × g for 10 min at 4 °C, and then filtered through a 0.22 µm filter to remove protein; the urine samples were boiled at 100 °C for 15 min, centrifuged at 13 000 × g for 10 min at 4 °C, and then filtered through a 0.22 µm filter to remove protein. LC‐MS/MS determination was performed consistent with the method used for the analysis of l‐2‐HG in cell extracts as described above.

### Determination of l‐2‐HG in Human Body Fluids Using sfLHGFR_L_

For the determination of l‐2‐HG in human body fluids using sfLHGFR_L_, the serum samples and urine samples were diluted to appropriate concentrations by 100 mm HEPES buffer containing 100 mm KCl (pH 7.4). Purified sfLHGFR_L_ (0.2 µm) were mixed directly with the diluted samples in a black 96‐well plate or 384‐well plate (PerkinElmer, USA) at a volume ratio of 3:1 (total 100 µL per well for 96‐well plate and 20 µL per well for 384‐well plate). The fluorescence ratio changes of sfLHGFR_L_ were determined immediately by an EnSight microplate reader using 405 or 488 nm excitation and 528 nm emission. Absolute quantification of l‐2‐HG was performed using a standard curve, which was determined for each experiment.

### Statistical Analysis

The software used for initial data processing was Microsoft Excel 2016, and subsequent analyses were performed using OriginPro 2019 (OriginLab, USA), Graphpad Prism 5 (Graphpad Software, USA), and Graphpad Prism 7 (Graphpad Software, USA). Fluorescence intensity measured by microplate reader was accomplished based on Kaleido 3.0 (PerkinElmer, USA). Imaging data were acquired and processed by Zen 3.1 (Zeiss, Germany) and ImageJ (National Institutes of Health, USA), respectively. All data shown are means ± standard deviations, and a two‐tailed *t*‐test was used for significant difference analysis, * *p* < 0.05; ** *p* < 0.01; *** *p* < 0.001; ns, no significant difference (*p* ≥ 0.05). Similar results were obtained from at least three independent experiments in all experiments. Detailed data analysis is described in the text.

### Ethical Statement

This study complied with all relevant ethical regulations. The study protocol was approved by the Shandong University Qilu Hospital Ethics Committee (approval number KYLL‐2021(KS)−192). All subjects were fully informed.

## Conflict of Interest

C.G., Z.K., C.M., and P.X. are inventors of patent applications (Chinese patent application no. 2023114979187). The patent was submitted by Shandong University. Other authors declare no relevant conflicts of interest.

## Author Contributions

C.G., C.M., C.L., and P.X. designed the research. Z.K., S.H., K.G., Y.L., W.Z., X.X., and R.X. performed the research. N.Z. and Z.F. prepared the clinical samples from patients with kidney cancer. Z.K. and C.G. analyzed the data. Z.K., C.G., C.M., and P.X. wrote the manuscript.

## Supporting information

Supporting Information

## Data Availability

The data that support the findings of this study are available from the corresponding author upon reasonable request.
