# Supplementary material for: An Ultrasensitive Biosensor for Probing Subcellular Distribution and Mitochondrial Transport of l‐2‐Hydroxyglutarate
Source: Adv Sci (Weinh). 2024 Jul 15;11(35):2404119. doi: 10.1002/advs.202404119 (PMC11425224; doi:10.1002/advs.202404119)
Supplement: Supplementary file 1 — Supporting Information [file ADVS-11-2404119-s001.pdf]

Supporting Information

An Ultrasensitive Biosensor for Probing Subcellular Distribution and Mitochondrial Transport of L-2-Hydroxyglutarate

*Zhaoqi Kang,[a] Shuang Hou,[a] Kaiyu Gao,[a] Yidong Liu,[a] Ning Zhang,[c] Zhiqing Fang,[d] Wen Zhang,[e] Xianzhi Xu,[a] Rong Xu,[a] Chuanjuan Lü,[a] Cuiqing Ma,[a] Ping Xu,[b] and Chao Gao[a],***

[a] Z. Kang, S. Hou, K. Gao, Y. Liu, X. Xu, R. Xu, C. Lü, C. Ma, C. Gao
State Key Laboratory of Microbial Technology, Shandong University, Qingdao, People's Republic of China
*Corresponding author: C. Gao, E-mail: jieerbu@sdu.edu.cn

[b] P. Xu
State Key Laboratory of Microbial Metabolism, Joint International Research Laboratory of Metabolic & Developmental Sciences, and School of Life Sciences & Biotechnology, Shanghai Jiao Tong University, Shanghai, People's Republic of China

[c] N. Zhang
Department of Breast Surgery, General Surgery, Qilu Hospital, Cheeloo College of Medicine, Shandong University, Jinan, People's Republic of China

[d] Z. Fang
Department of Urology, Qilu Hospital, Cheeloo College of Medicine, Shandong University, Jinan, People's Republic of China

[e] W. Zhang
Institute of Medical Sciences, The Second Hospital, Cheeloo College of Medicine, Shandong University, Jinan, People's Republic of China

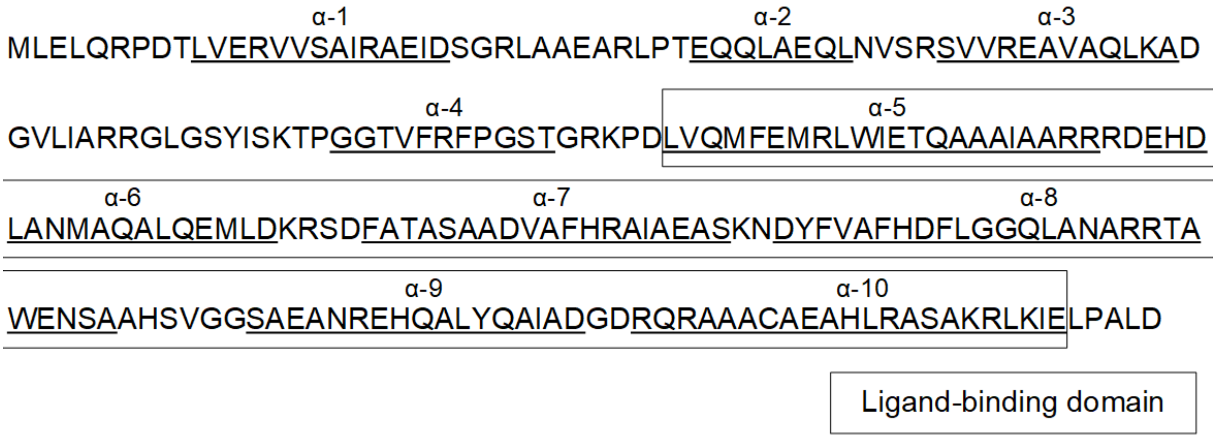

Figure S1 The predicted secondary structure of LhgR. The α-helices of LhgR were indicated with underlines and the corresponding designations were listed above the sequence. The ligand-binding domain of LhgR was boxed in black.

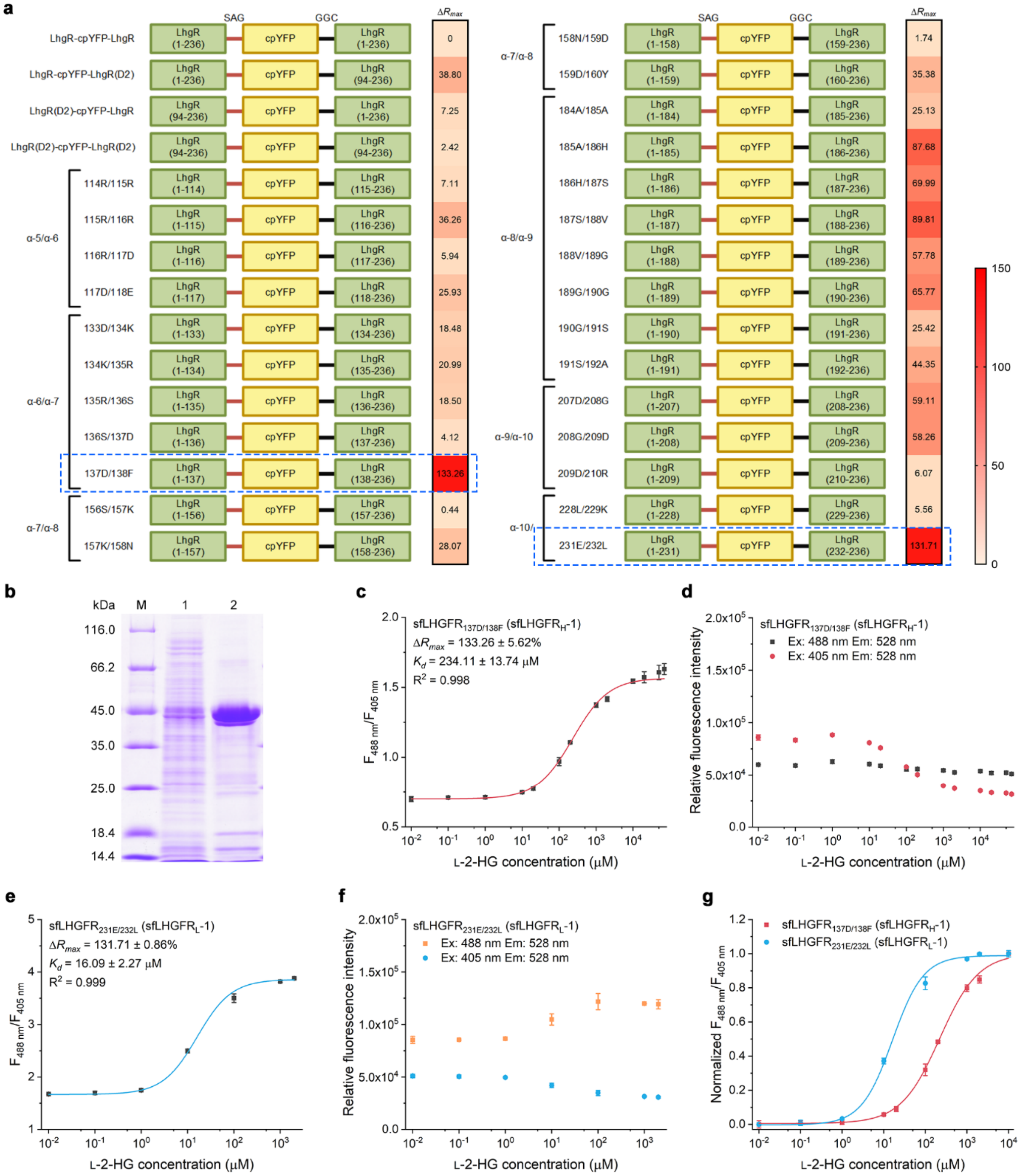

Figure S2 Construction of sfLHGFR$_H$-1 and sfLHGFR$_L$-1 by insertion sites screening (Step 1). (a) Schematic representation of the construction of the L-2-HG biosensor sfLHGFR based on

insertion sites screening. The ΔR_{max} of different biosensor variants to L-2-HG were presented in a heatmap, where the color depth represented the magnitude of ΔR_{max}. LhgR and LhgR(D2) indicated intact LhgR and LhgR with truncated DNA-binding domain, respectively. Taking sfLHGFR$_H$-1 (corresponding to 137D/138F) as an example, α-6/α-7 referred to the insertion site of cpYFP located between the predicted 6th and 7th α-helix of LhgR, and 137D/138F referred to the insertion site of cpYFP located between the amino acid residues 137 (Asp) and 138 (Phe) of LhgR. The structures of sfLHGFR$_H$-1 and sfLHGFR$_L$-1 (corresponding to 231E/232L) were shown in blue dotted boxes. **(b)** SDS-PAGE analysis of the purification of sfLHGFR$_H$-1. Lane M, molecular weight markers; lane 1, crude extract of *E. coli* BL21(DE3) harboring pETDuet-sfLHGFR$_H$-1; lane 2, purified His$_6$-tagged sfLHGFR$_H$-1 using a HisTrap column. **(c, d)** Dose-response curve **(c)** and fluorescence intensity changes **(d)** of sfLHGFR$_H$-1 for increasing concentrations (10 nM to 70 mM) of L-2-HG. **(e, f)** Dose-response curve **(e)** and fluorescence intensity changes **(f)** of sfLHGFR$_L$-1 for increasing concentrations (10 nM to 2 mM) of L-2-HG. **(g)** Comparison of the dose-response curves of sfLHGFR$_H$-1 and sfLHGFR$_L$-1 for L-2-HG. All data shown are means ± standard deviations (s.d.) (n = 3 independent experiments).

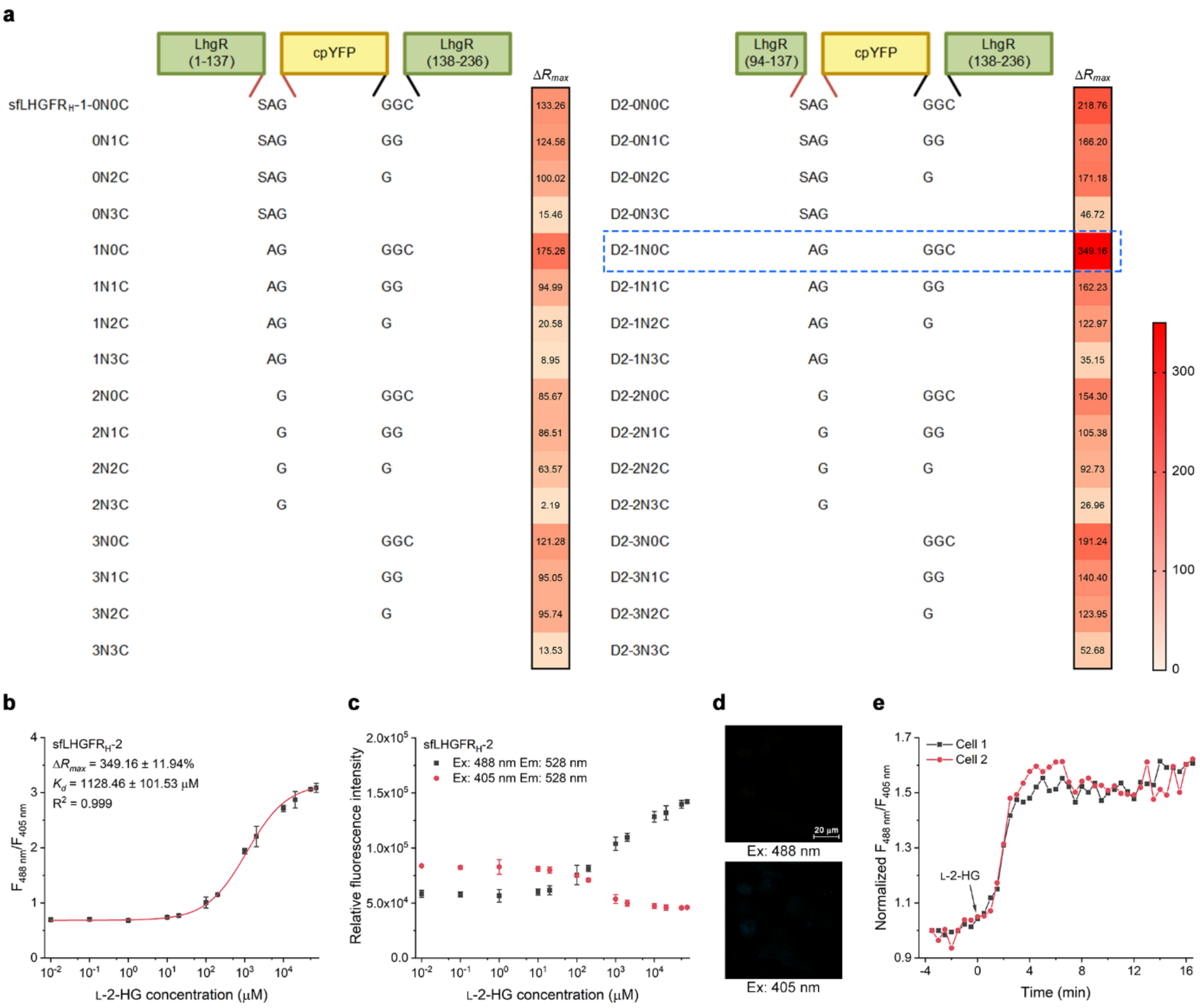

Figure S3 Construction of sfLHGFR$_H$-2 by linker and DNA-binding domain truncation (Step 2). (a) Schematic representation of the construction of sfLHGFR$_H$-2 and its variants based on linker and DNA-binding domain truncation. The ΔR_{max} of different biosensor variants to L-2-HG were presented in a heatmap, where the color depth represented the magnitude of ΔR_{max}. 0N0C and D2-1N0C referred to sfLHGFR$_H$-1 and sfLHGFR$_H$-2, respectively, wherein the numbers indicated the number of amino acids truncated from the N- and C-terminal linker of cpYFP, and D2 indicated the removal of the DNA-binding domain of LhgR. **(b, c)** Dose-response curve **(b)** and fluorescence intensity changes **(c)** of sfLHGFR$_H$-2 for increasing

concentrations (10 nM to 70 mM) of L-2-HG. **(d)** Analysis of the expression of sfLHGFR$_H$-2 in HEK293FT cells. Fluorescence images of HEK293FT cells expressing sfLHGFR$_H$-2 were acquired with excitation at 488 nm and 405 nm. The 488 nm laser and the 405 nm laser used for imaging were set to 0.1% power and 0.4% power, respectively. **(e)** Analysis of the function of sfLHGFR$_H$-2 in HEK293FT cells. Time course of the fluorescence ratio changes of single HEK293FT cell expressing sfLHGFR$_H$-2 in response to exogenous 10 mM L-2-HG addition was recorded. L-2-HG was added at time point zero (min). Data were normalized to the initial ratio. All data shown are means ± s.d. (n = 3 independent experiments).

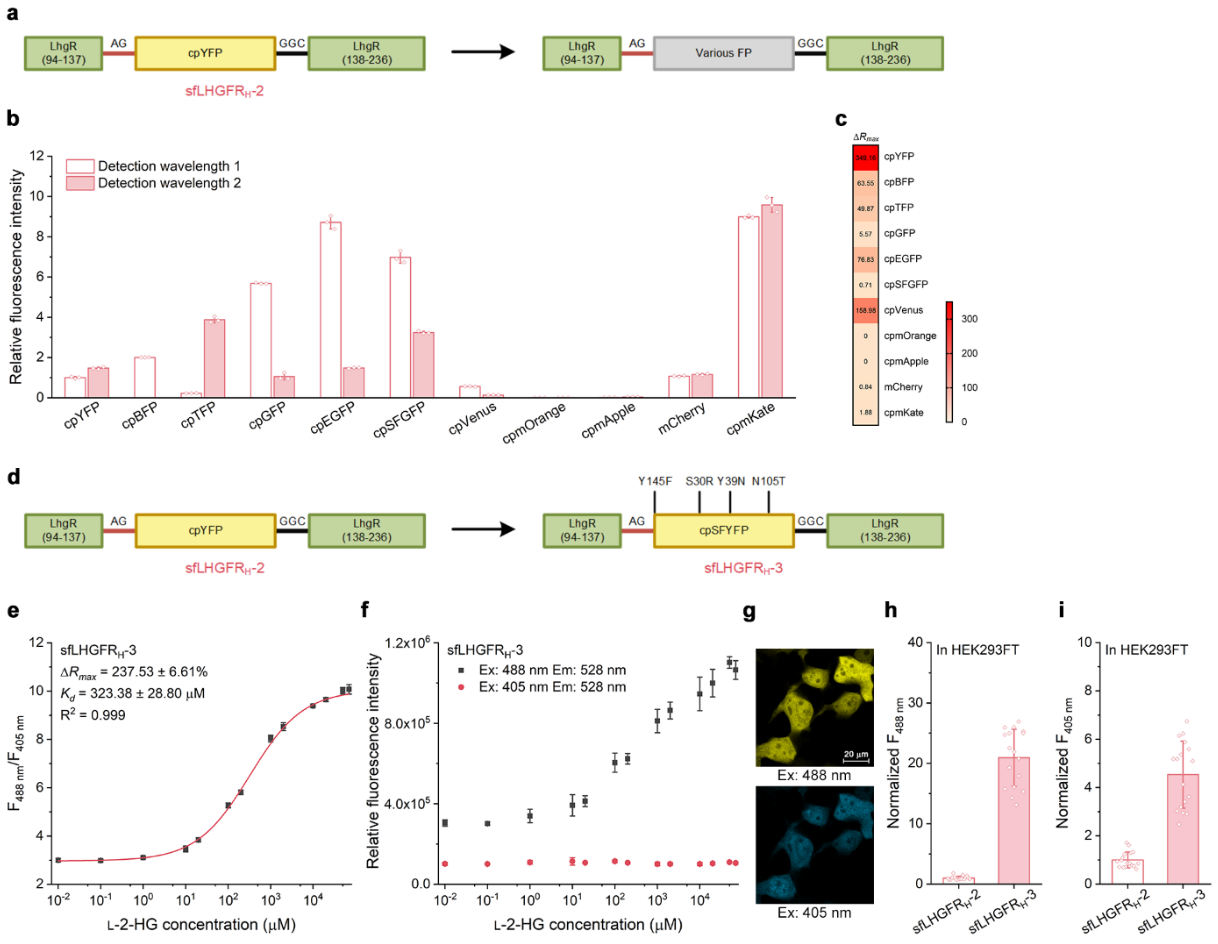

Figure S4 Construction of sfLHGFR$_H$-3 by superfolder sites substitution (Step 3). (a) Schematic representation of the optimization of fluorescence brightness of sfLHGFR$_H$-2 by fluorescent protein replacement. **(b)** Comparison of fluorescence intensities of different biosensor variants obtained by fluorescent protein replacement under respective detection conditions. The excitation and emission wavelengths for each fluorescent protein were as follows: cpYFP, excitation at 488 nm and 405 nm, emission at 528 nm; cpBFP, excitation at 462 nm, emission at 510 nm; cpTFP, excitation at 462 nm and 400 nm, emission at 510 nm; cpGFP, excitation at 488 nm and 405 nm, emission at 528 nm; cpEGFP, excitation at 488 nm and 405 nm, emission at 528 nm; cpSFGFP, excitation at 488 nm and 405 nm, emission at 528 nm;

cpVenus, excitation at 488 nm and 405 nm, emission at 528 nm; cpmOrange, excitation at 512 nm and 520 nm, emission at 562 nm and 550 nm; cpmApple, excitation at 445 nm and 577 nm, emission at 600 nm; mCherry, excitation at 550 nm and 587 nm, emission at 610 nm; cpmKate, excitation at 584 nm and 536 nm, emission at 620 nm. Data were normalized to the fluorescence intensity of sfLHGFR$_H$-2 (corresponding to cpYFP) at detection wavelength 1 (excitation at 488 nm and emission at 528 nm). **(c)** Comparison of the ΔR_{max} of different fluorescent protein replacement variants for L-2-HG. Data were presented in a heatmap, where the color depth represented the magnitude of ΔR_{max}. **(d)** Schematic representation of the construction of sfLHGFR$_H$-3 based on superfolder sites substitution. **(e, f)** Dose-response curve **(e)** and fluorescence intensity changes **(f)** of sfLHGFR$_H$-3 for increasing concentrations (10 nM to 70 mM) of L-2-HG. **(g)** Analysis of the expression of sfLHGFR$_H$-3 in HEK293FT cells. Fluorescence images of HEK293FT cells expressing sfLHGFR$_H$-3 were acquired with excitation at 488 nm and 405 nm. The 488 nm laser and the 405 nm laser used for imaging were set to 0.1% power and 0.4% power, respectively. **(h, i)** Comparison of the fluorescence brightness of sfLHGFR$_H$-2 and sfLHGFR$_H$-3 expressed in HEK293FT cells with excitation at 488 nm **(h)** and 405 nm **(i)**. HEK293FT cells expressing sfLHGFR$_H$-2 and sfLHGFR$_H$-3 were imaged with the same parameters. Data were normalized to the fluorescence intensity of sfLHGFR$_H$-2. All data shown are means ± s.d. (n = 3 independent experiments for **b**, **e**, and **f**; n = 17 cells for **h** and **i**).

a

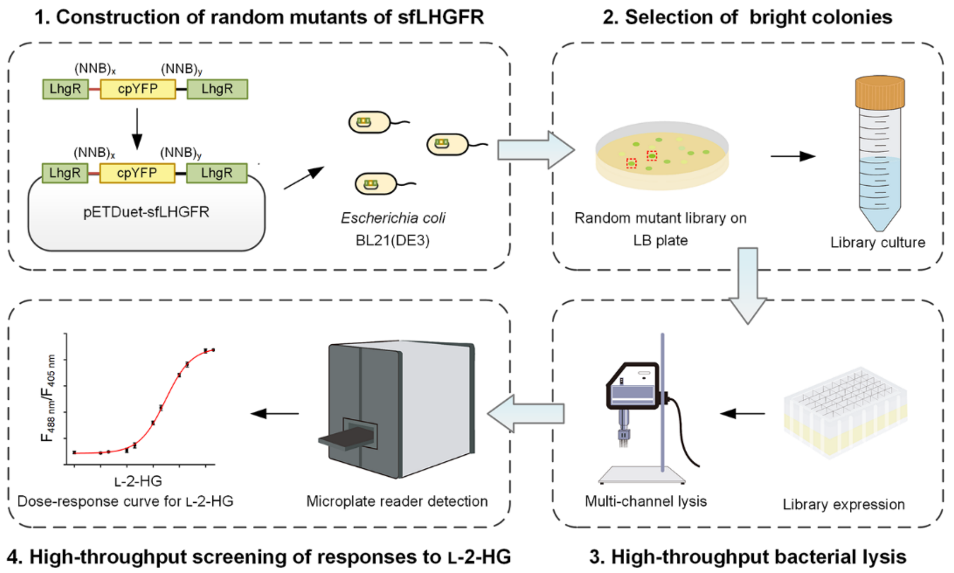

b

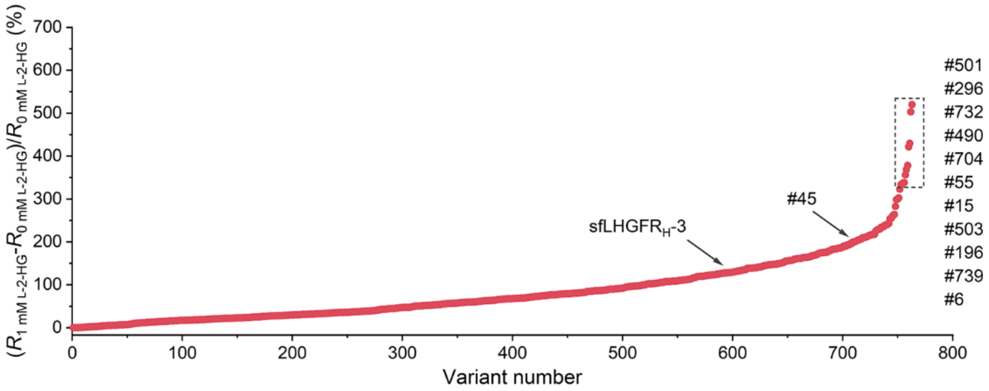

c

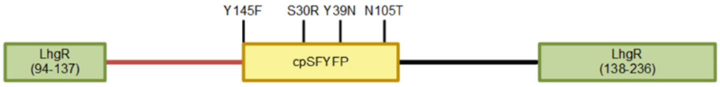

	N-terminal linker		C-terminal linker		ΔR_{max}
sfLHGFR$_H$-3	A G		G G C		237.53 ± 6.61%
#6	R I		G G C		490.38 ± 12.32%
#15	P D		L S H		909.23 ± 16.19%
#45	G G		G G C		361.04 ± 3.63%
#55	K V		G G C		538.29 ± 18.08%
#196	F L S		G G C		675.38 ± 6.60%
#296	C L N		T L F		1,486.10 ± 23.94%
#490	V P		S L		842.67 ± 51.94%
#501	I S		R Y		1,125.64 ± 37.72%
#503	I G		R T		769.22 ± 28.06%
#704	R		N I		773.98 ± 45.35%
#732			D P		797.43 ± 8.13%
#739			S V		625.67 ± 20.07%

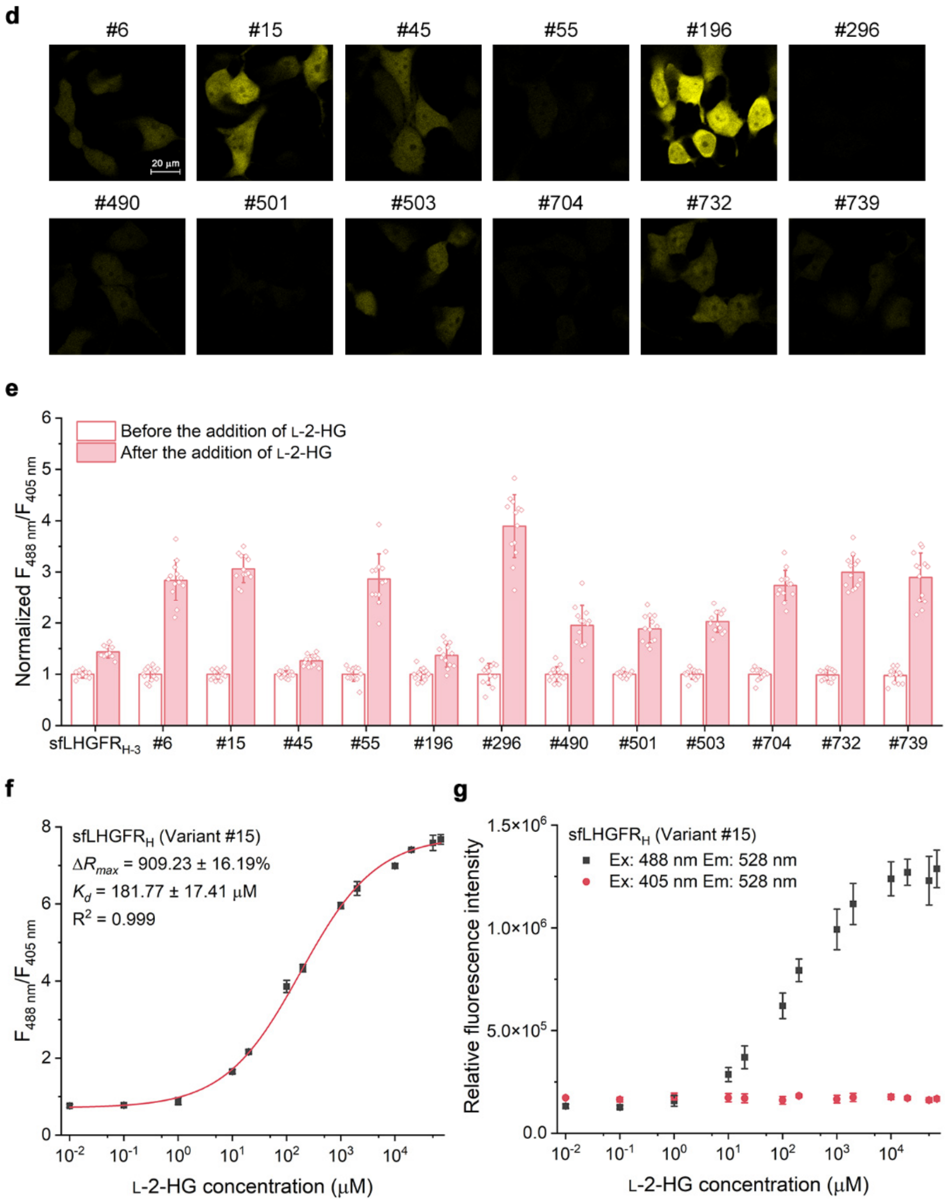

Figure S5 Construction of sfLHGFR$_H$ by random linker mutagenesis (Step 4). (a) Schematic representation of the workflow for the optimization of the L-2-HG biosensor through random mutation and high-throughput screening. This figure was generated using BioRender. **(b)** Comparison of the fluorescence responses of 763 random mutants screened to 1 mM L-2-HG. Twelve random mutants with enhanced fluorescence response were labeled in the figure. **(c)** The

linker sequences of the twelve random mutants obtained and their ΔR_{max} for L-2-HG. **(d)** Analysis of the expression of the twelve random mutants in HEK293FT cells. Fluorescence images of HEK293FT cells expressing each of the twelve random mutants were acquired with excitation at 488 nm. The 488 nm laser used for imaging was set to 0.1% power. **(e)** Analysis of the fluorescence responses of the twelve random mutants expressed in HEK293FT cells to L-2-HG. The fluorescence ratio changes of HEK293FT cells expressing different mutants in response to exogenous 6 mM L-2-HG addition were determined. Data were normalized to the ratio before the addition of L-2-HG. **(f, g)** Dose-response curve **(f)** and fluorescence intensity changes **(g)** of sfLHGFR$_{H}$ (corresponding to variant #15) for increasing concentrations (10 nM to 70 mM) of L-2-HG. All data shown are means ± s.d. (n = 3 independent experiments for **c**, **f**, and **g**; n ≥ 10 cells for **e**).

sfLHGFR$_H$:

MLVQMFEMRLWIETQAAAIAARRRDEHDLANMAQALQEMLDKRSDPDFNSDNVYIMADKQK

NGIKANFKIRHNVEDGSVQLADHYQQNTPIGDGPVLLPDNHYLSFQSVLSKDPNEKRDHMVL

LEFVTAAGITLGMDELYNVDGGSGGTGSKGEELFTGVVPILVELDGDVNGHKFSVRGEGEG

DATNGKLTLKLICTTGKLPVPWPTLVTTLGYGLKCFARYPDHMKQHDFFKSAMPEGYVQERT

IFFKDDGTYKTRAEVKFEGDTLVNRIELKGIGFKEDGNILGHKLEYNLSHFATASAADVAFHRA

IAEASKNDYFVAFHDFLGGQLANARRTAWENSAAHSVGGSAEANREHQALYQAIADGDRQR

AAACAEAHLRASAKRLKIELPALD*

Figure S6 Full protein sequence of sfLHGFR$_H$. The sequence of cpSFYFP was indicated with a wavy underline. The N-terminal and C-terminal linkers of cpSFYFP were indicated with straight underlines. The substituted four superfolder sites were indicated with "*".

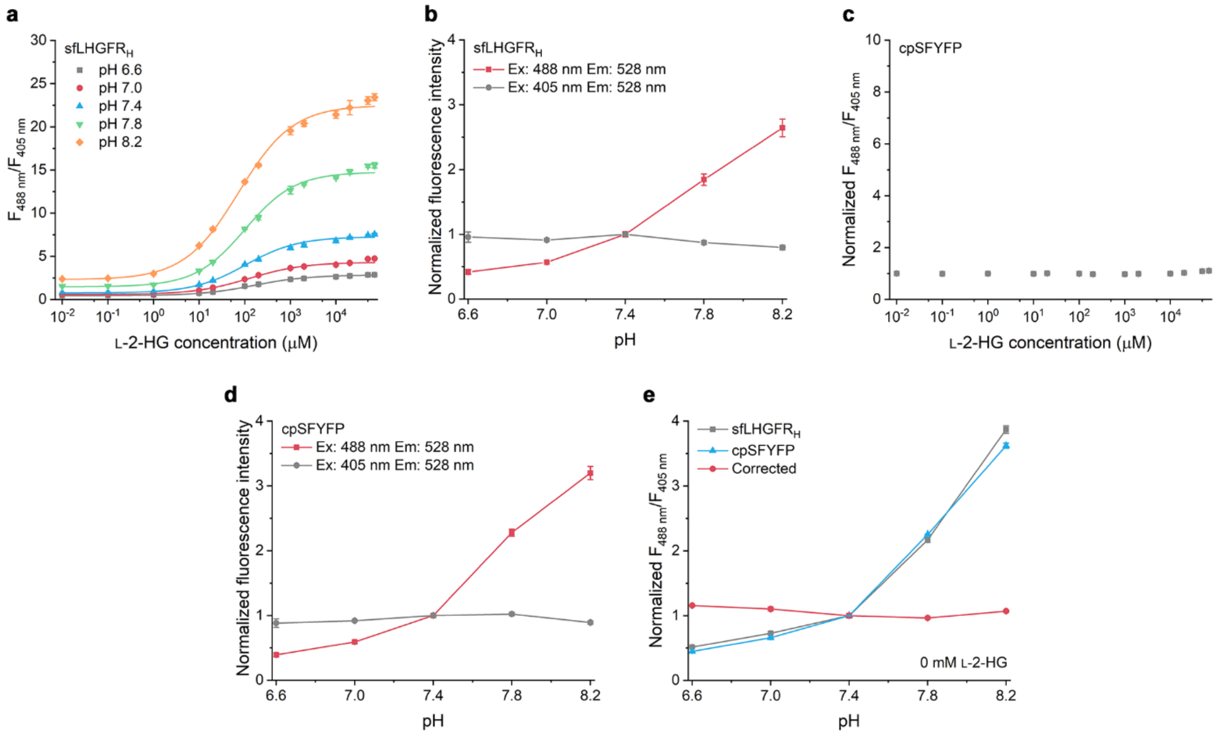

Figure S7 pH-stability analysis of sfLHGFR$_H$. (a) Dose-response curves of sfLHGFR$_H$ for increasing concentrations (10 nM to 70 mM) of L-2-HG at the indicated pH values. **(b)** Fluorescence intensities of sfLHGFR$_H$ at the indicated pH values. Data were normalized to the fluorescence at pH 7.4. **(c)** Response of cpSFYFP for increasing concentrations (10 nM to 70 mM) of L-2-HG. **(d)** Fluorescence intensities of cpSFYFP at the indicated pH values. Data were normalized to the fluorescence at pH 7.4. **(e)** pH-correction of the fluorescence ratio of sfLHGFR$_H$ by cpSFYFP in the absence of L-2-HG. Data were normalized to the ratio at pH 7.4. All data shown are means ± s.d. ($n \geq 3$ independent experiments).

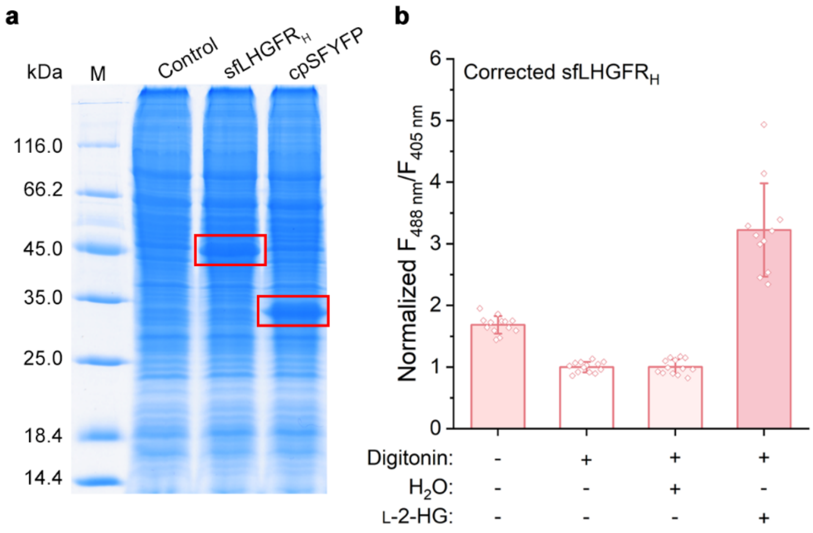

Figure S8 Identification of the function of sfLHGFR$_H$ in HEK293FT cells. (a) SDS-PAGE analysis of the expression of sfLHGFR$_H$ and cpSFYFP in HEK293FT cells. Lane M, molecular weight markers; lane Control, crude extract of HEK293FT cells; lane sfLHGFR$_H$, crude extract of HEK293FT cells harboring pcDNA3.1$^{(+)}$-sfLHGFR$_H$; lane cpSFYFP, crude extract of HEK293FT cells harboring pcDNA3.1$^{(+)}$-cpSFYFP. **(b)** Analysis of the function of sfLHGFR$_H$ in HEK293FT cells. 80 μM digitonin and 5 mM L-2-HG or an equal volume of distilled water were sequentially added into the imaging medium of sfLHGFR$_H$-expressing HEK293FT cells, and then the fluorescence ratios were determined. Data were corrected by cpSFYFP and normalized to the ratio in the presence of only digitonin. All data shown are means ± s.d. (n = 13, 13, 13, and 11 cells from left to right).

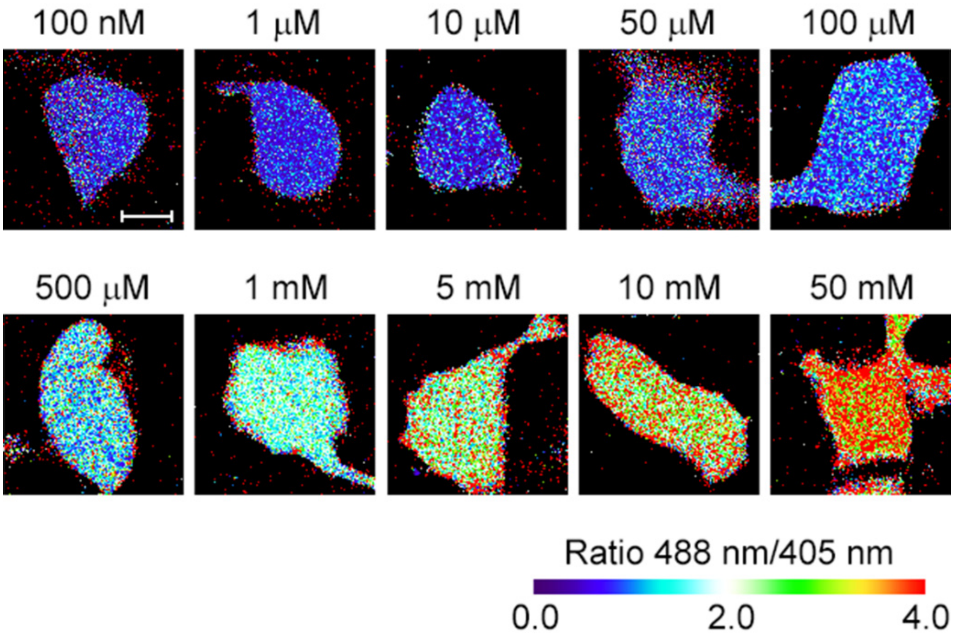

Figure S9 Confocal microscopy imaging of single HEK293FT cell expressing sfLHGFR$_H$ in response to exogenous increasing concentrations of L-2-HG addition. Scale bar, 10 μm.

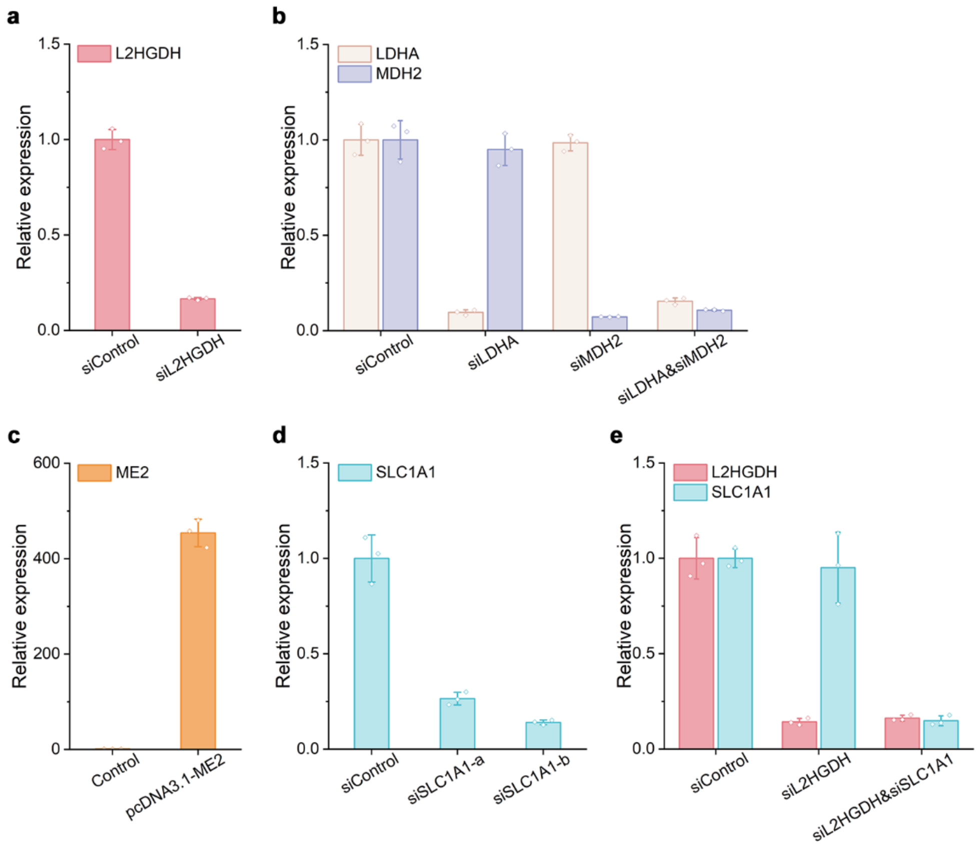

Figure S10 qPCR analysis of gene knockdown or overexpression in HEK293FT cells. (a) Analysis of L2HGDH expression in HEK293FT cells treated with siRNA targeting L2HGDH. **(b)** Analysis of LDHA and MDH2 expression in HEK293FT cells treated with siRNA targeting LDHA and/or MDH2. **(c)** Analysis of ME2 expression in HEK293FT cells transfected with ME2 overexpression plasmid. **(d)** Analysis of SLC1A1 expression in HEK293FT cells treated with siRNA targeting SLC1A1. **(e)** Analysis of L2HGDH and SLC1A1 expression in HEK293FT cells treated with siRNA targeting L2HGDH and/or SLC1A1. All data shown are means ± s.d. (n = 3 independent experiments).

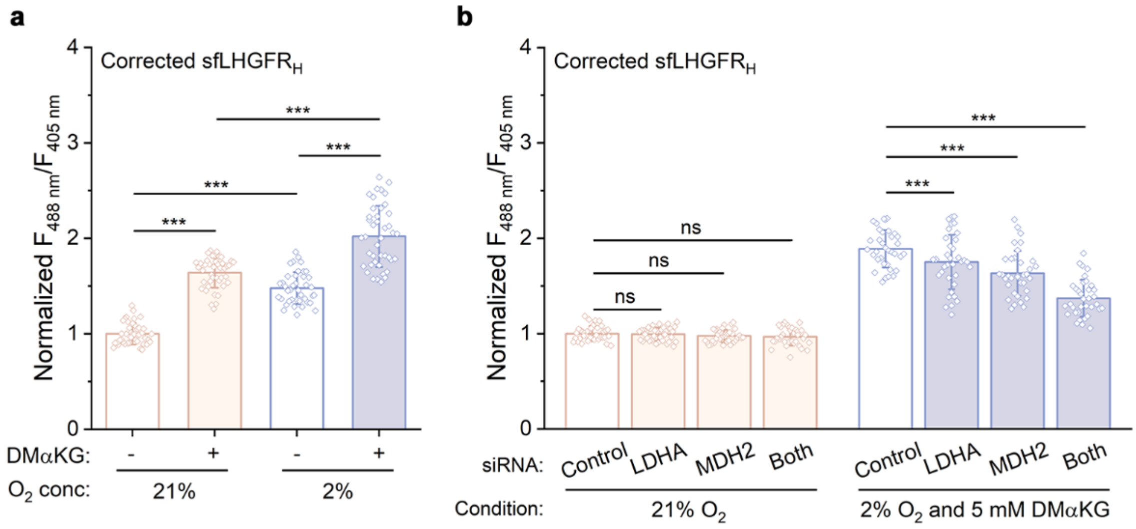

Figure S11 Analysis of hypoxia-mediated L-2-HG fluctuations in living cells using sfLHGFR$_H$. (a) Analysis of hypoxia-induced L-2-HG production using sfLHGFR$_H$. The fluorescence ratios of sfLHGFR$_H$ were determined 24 h after incubation of cells with 21% oxygen or 2% oxygen as well as in the absence or presence of 5 mM dimethyl-2-ketoglutarate (DMαKG). Data were corrected by cpSFYFP and normalized to the normoxic condition without DMαKG. **(b)** Analysis of the anabolic mechanism of L-2-HG using sfLHGFR$_H$. sfLHGFR$_H$-expressing HEK293FT cells were treated with different siRNAs for 24 h, and then cultured under the indicated conditions for another 24 h. Data were corrected by cpSFYFP and normalized to the normoxic condition treated with negative siRNA. All data shown are means ± s.d. (n = 42, 42, 40, and 42 cells from left to right in **a**; n = 35 cells in **b**). ***, $P < 0.001$; ns, no significant difference ($P \geq 0.05$) in two-tailed t test.

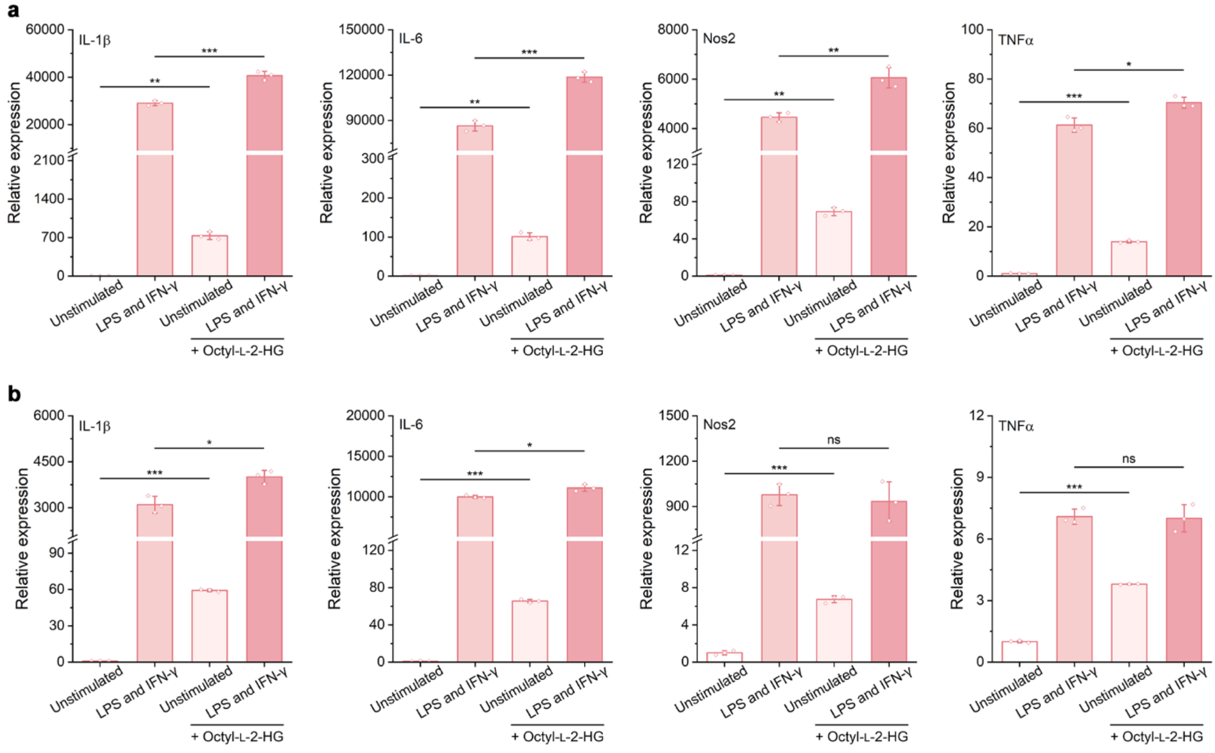

Figure S12 Biological function analysis of L-2-HG in M1 macrophages. (a, b) qPCR analysis of the expression of IL-1β, IL-6, Nos2, and TNFα in unstimulated M0 macrophages and LPS + IFN-γ-stimulated M1 macrophages. Data were analyzed 6 h **(a)** and 24 h **(b)** after LPS + IFN-γ stimulation. M0 macrophages were pretreated with 500 μM octyl-L-2-HG or equal volume of DMSO 3 h before the addition of LPS (1 μg mL^{-1}) and IFN-γ (20 ng mL^{-1}) or equal volume of distilled water. All data shown are means ± s.d. (n = 3 independent experiments). *, $P < 0.05$; **, $P < 0.01$; ***, $P < 0.001$; ns, no significant difference ($P \geq 0.05$) in two-tailed *t* test.

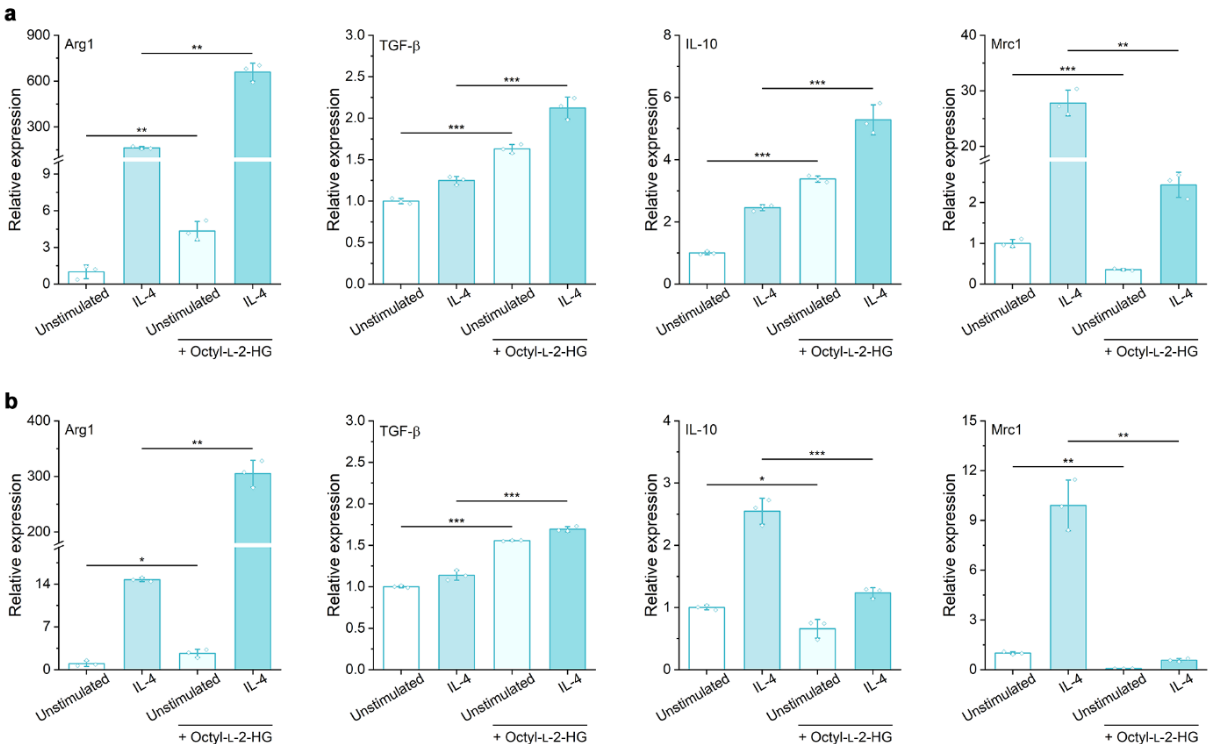

Figure S13 Biological function analysis of L-2-HG in M2 macrophages. (a, b) qPCR analysis of the expression of Arg1, TGF-β, IL-10, and Mrc1 in unstimulated M0 macrophages and IL-4-stimulated M2 macrophages. Data were analyzed 6 h **(a)** and 24 h **(b)** after IL-4 stimulation. M0 macrophages were pretreated with 500 μM octyl-L-2-HG or equal volume of DMSO 3 h before the addition of IL-4 (20 ng mL^{-1}) or equal volume of distilled water. All data shown are means ± s.d. (n = 3 independent experiments). *, $P < 0.05$; **, $P < 0.01$; ***, $P < 0.001$; ns, no significant difference ($P \geq 0.05$) in two-tailed t test.

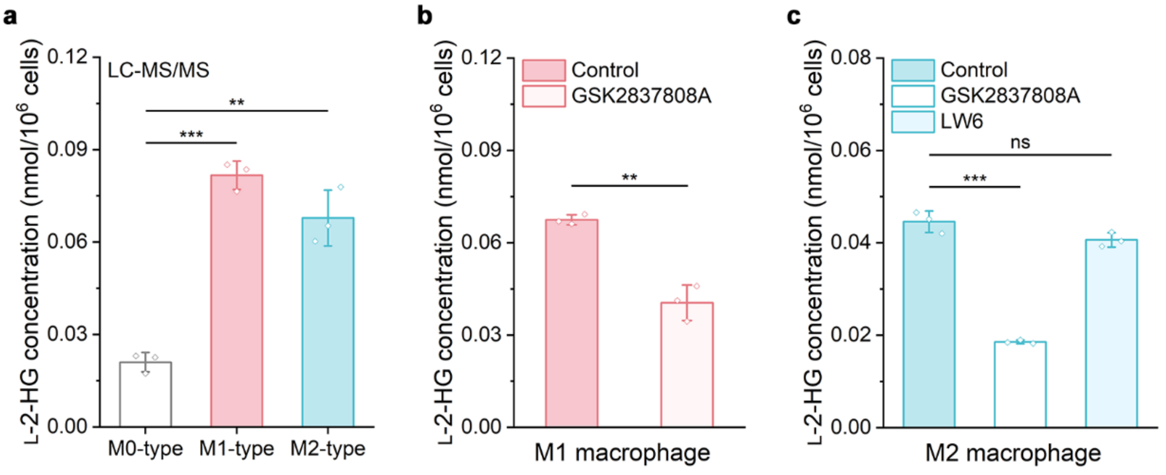

Figure S14 Detection of intracellular L-2-HG concentrations in macrophages using LC-MS/MS. (a) Detection of L-2-HG differences between differently polarized macrophages using LC-MS/MS. **(b)** Detection of the effects of LDHA inhibitor GSK2837808A on the concentration of L-2-HG in M1 macrophages using LC-MS/MS. M0 macrophages were pretreated with 85 μM GSK2837808A or equal volume of DMSO for 2 h, and then stimulated with LPS (1 μg mL^{-1}) and IFN-γ (20 ng mL^{-1}) for 24 h. **(c)** Detection of the effects of LDHA inhibitor GSK2837808A and MDH2 inhibitor LW6 on the concentration of L-2-HG in M2 macrophages using LC-MS/MS. M0 macrophages were pretreated with 85 μM GSK2837808A, 65 μM LW6, or equal volume of DMSO for 2 h, and then stimulated with IL-4 (20 ng mL^{-1}) for 24 h. All data shown are means ± s.d. (n = 3 independent experiments). **, $P < 0.01$; ***, $P < 0.001$; ns, no significant difference ($P \geq 0.05$) in two-tailed t test.

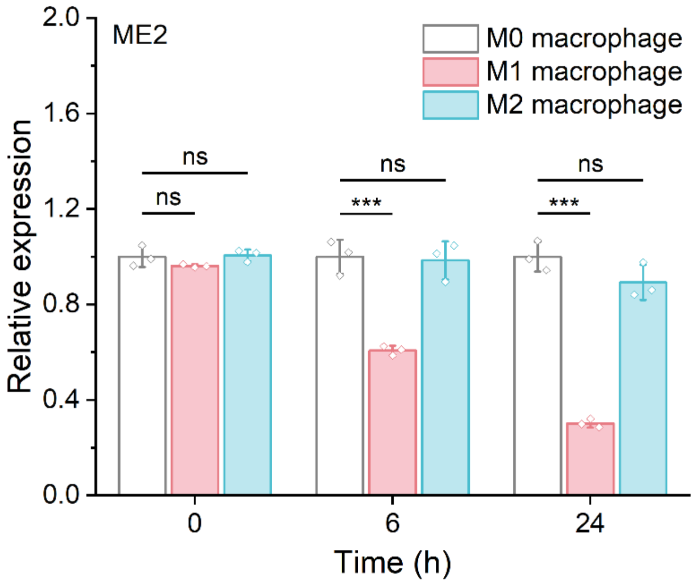

Figure S15 qPCR analysis of the expression of ME2 in differently polarized macrophages. Data were analyzed 0, 6, and 24 h after inducers stimulation. All data shown are means ± s.d. (n = 3 independent experiments). ***, $P < 0.001$; ns, no significant difference ($P \geq 0.05$) in two-tailed *t* test.

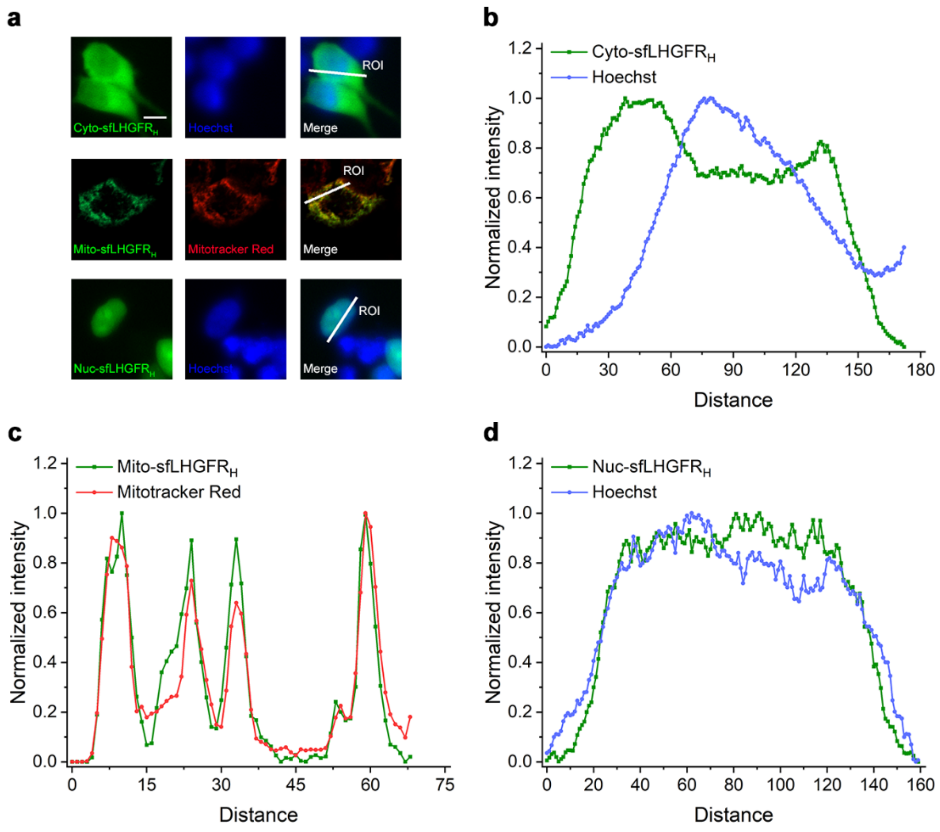

Figure S16 Subcellular localization analysis of sfLHGFR$_H$. (a) Fluorescence images of HEK293FT cells expressing Cyto-sfLHGFR$_H$, Mito-sfLHGFR$_H$, or Nuc-sfLHGFR$_H$ co-stained with Hoechst, Mitotracker red, or Hoechst. Scale bar, 10 μm. **(b-d)** Plot profile of HEK293FT cells expressing Cyto-sfLHGFR$_H$ **(b)**, Mito-sfLHGFR$_H$ **(c)**, or Nuc-sfLHGFR$_H$ **(d)** co-stained with Hoechst, Mitotracker red, or Hoechst. Data were collected from the region of interest (ROI) in panel **a**.

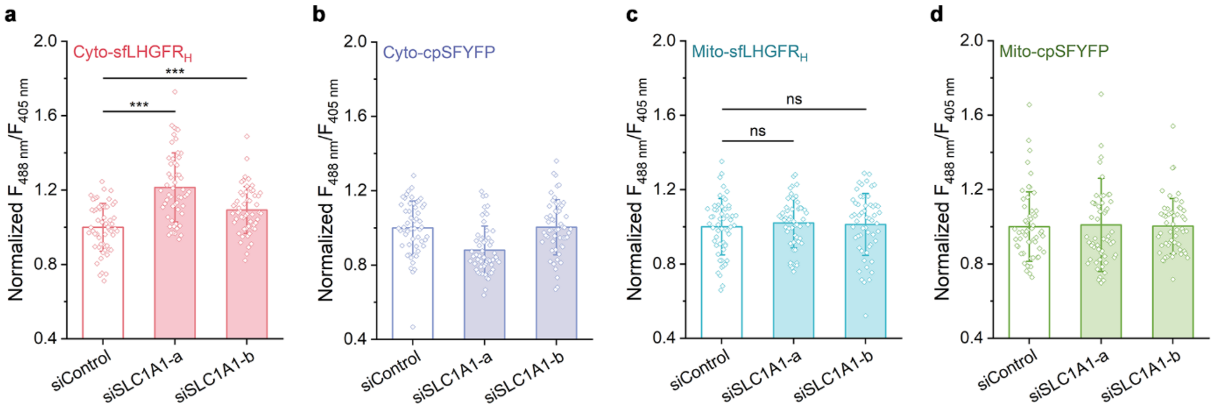

Figure S17 Analysis of the biological functions of SLC1A1 in the mitochondrial L-2-HG uptake using sfLHGFR$_H$. (a-d) The fluorescence ratio changes of Cyto-sfLHGFR$_H$ **(a)**, Cyto-cpSFYFP **(b)**, Mito-sfLHGFR$_H$ **(c)**, and Mito-cpSFYFP **(d)** were determined 48 h after SLC1A1 knockdown. Data were normalized to the control condition treated with negative siRNA. All data shown are means ± s.d. (n = 55 cells). ***, $P < 0.001$; ns, no significant difference ($P \geq 0.05$) in two-tailed t test.

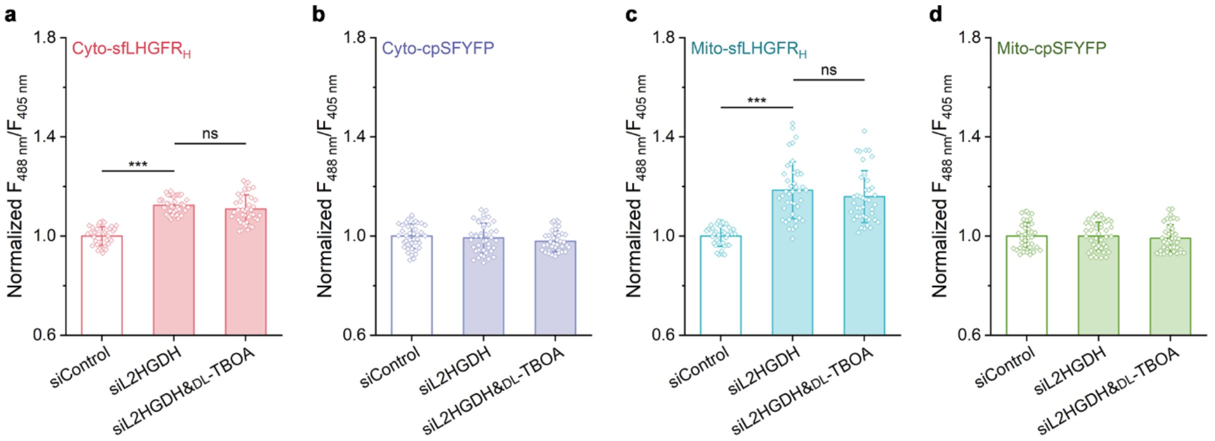

Figure S18 Analysis of the biological functions of SLC1A1 in the mitochondrial L-2-HG efflux using sfLHGFR$_H$. (a-d) The fluorescence ratio changes of Cyto-sfLHGFR$_H$ **(a)**, Cyto-cpSFYFP **(b)**, Mito-sfLHGFR$_H$ **(c)**, and Mito-cpSFYFP **(d)** were determined 48 h after L2HGDH knockdown. For the SLC1A1 pharmacological inhibition group, 100 μM DL-*threo*-β-benzyloxyaspartate (DL-TBOA) was added 24 h before imaging. Data were normalized to the control condition treated with negative siRNA. All data shown are means ± s.d. (n = 42 cells). ***, $P < 0.001$; ns, no significant difference ($P \geq 0.05$) in two-tailed t test.

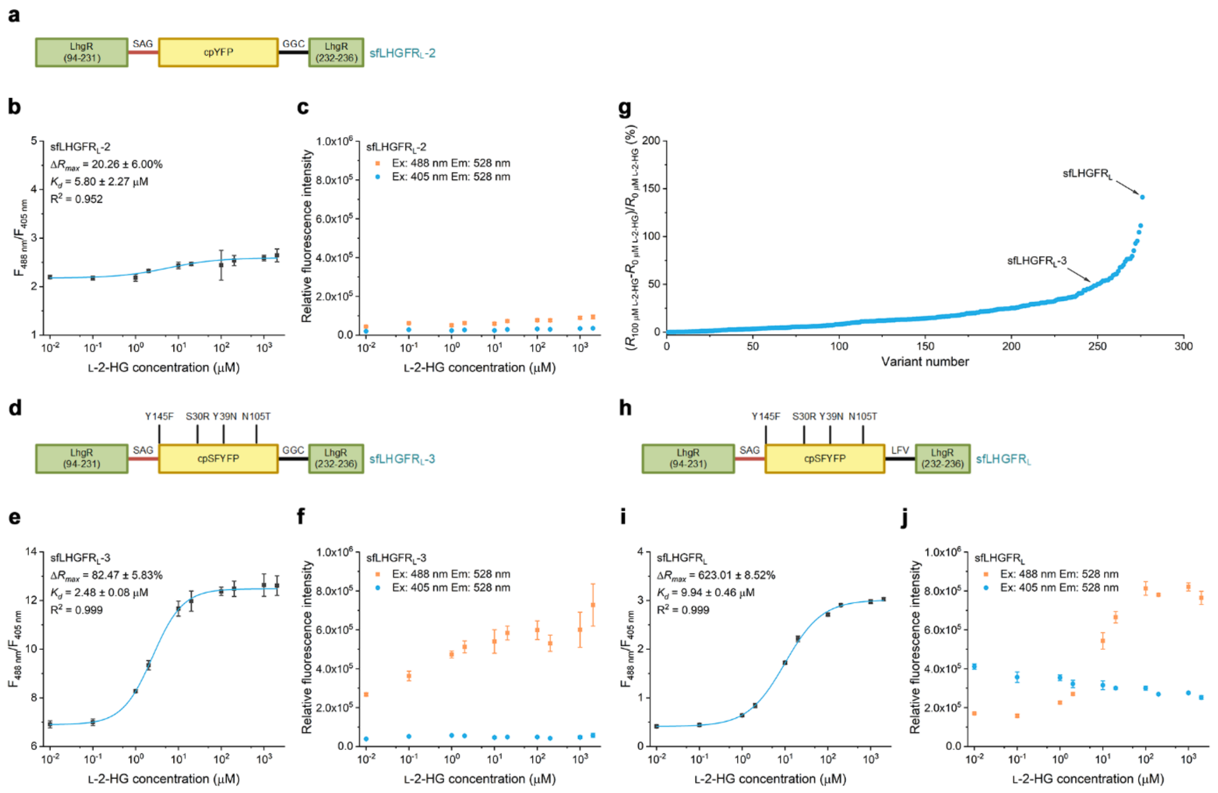

Figure S19 Construction of sfLHGFRL-2, sfLHGFRL-3, and sfLHGFRL (Step 2, Step 3, and Step 4). (a) Schematic representation of the structure of sfLHGFRL-2. **(b, c)** Dose-response curve **(b)** and fluorescence intensity changes **(c)** of sfLHGFRL-2 for increasing concentrations (10 nM to 2 mM) of L-2-HG. **(d)** Schematic representation of the structure of sfLHGFRL-3. **(e, f)** Dose-response curve **(e)** and fluorescence intensity changes **(f)** of sfLHGFRL-3 for increasing concentrations (10 nM to 2 mM) of L-2-HG. **(g)** Comparison of the fluorescence responses of 276 random mutants screened to 100 μM L-2-HG. **(h)** Schematic representation of the structure of sfLHGFRL. **(i, j)** Dose-response curve **(i)** and fluorescence intensity changes **(j)** of sfLHGFRL for increasing concentrations (10 nM to 2 mM) of L-2-HG. All data shown are means ± s.d. (n = 4 independent experiments).

sfLHGFR$_L$:

MLVQMFEMRLWIETQAAAIAARRRDEHDLANMAQALQEMLDKRSDFATASAADVAFHRAIA

EASKNDYFVAFHDFLGGQLANARRTAWENSAAHSVGGSAEANREHQALYQAIADGDRQRA

AACAEAHLRASAKRLKIESAGFNSDNVYIMADKQKNGIKANFKIRHNVEDGSVQLADHYQQN

TPIGDGPVLLPDNHYLSFQSVLSKDPNEKRDHMVLLEFVTAAGITLGMDELYNVDGGSGGTG

SKGEELFTGVVPILVELDGDVNGHKFSVRGEGEGDATNGKLTLKLICTTGKLPVPWPTLVTTL

GYGLKCFARYPDHMKQHDFFKSAMPEGYVQERTIFFKDDGTYKTRAEVKFEGDTLVNRIELK

GIGFKEDGNILGHKLEYNLFVLPALD*

Figure S20 Full protein sequence of sfLHGFR$_L$. The sequence of cpSFYFP was indicated with a wavy underline. The N-terminal and C-terminal linkers of cpSFYFP were indicated with straight underlines. The substituted four superfolder sites were indicated with “*”.

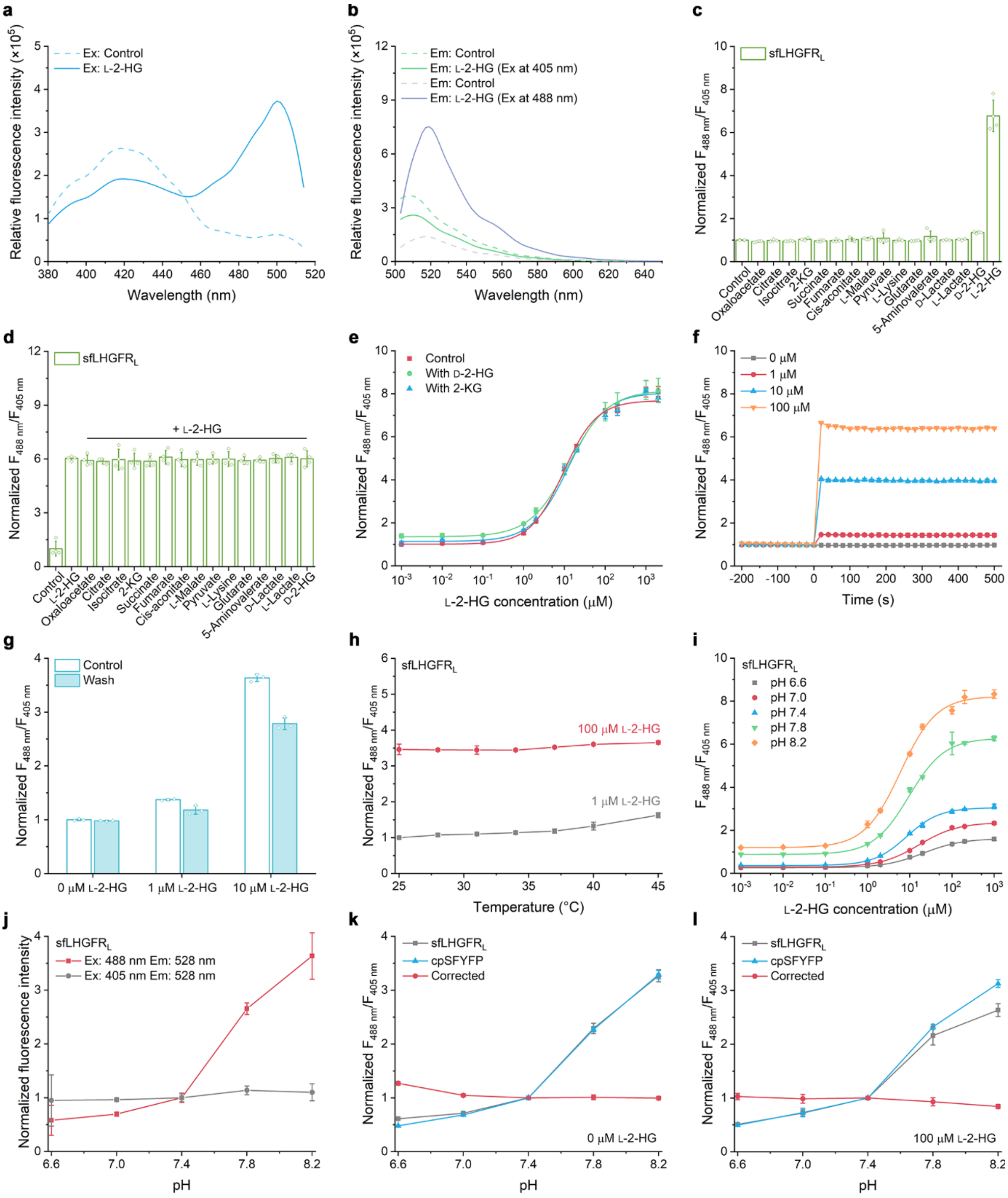

Figure S21 Characterization of sfLHGFRL. (a) Fluorescence excitation spectra changes of sfLHGFRL with (solid line) or without (dotted line) the addition of 1 mM L-2-HG. **(b)**

Fluorescence emission spectra changes of sfLHGFR$_L$ with (solid line) or without (dotted line) the addition of 1 mM L-2-HG at 405 nm (green line) or 488 nm excitation (purple line). **(c)** Specificity analysis of sfLHGFR$_L$. The fluorescence ratio changes of sfLHGFR$_L$ were determined in the presence of 80 μM indicated metabolites. Data were normalized to the control. **(d)** Influence of various metabolites on detection of L-2-HG by sfLHGFR$_L$. The fluorescence ratio changes of sfLHGFR$_L$ were determined in the absence of any metabolite (control), in the presence of only L-2-HG (L-2-HG), and in the presence of L-2-HG and 80 μM indicated metabolites. Data were normalized to the control. **(e)** Dose-response curves of sfLHGFR$_L$ for increasing concentrations (1 nM to 2 mM) of L-2-HG in the presence of 80 μM D-2-HG or 2-KG. Data were normalized to the initial ratio without D-2-HG and 2-KG. **(f)** Kinetics of the response of sfLHGFR$_L$ to L-2-HG. Different concentrations of L-2-HG were added at time point zero. Data were normalized to the initial ratio without L-2-HG. **(g)** Reversibility analysis of sfLHGFR$_L$. The fluorescence ratio changes of sfLHGFR$_L$ after L-2-HG addition and subsequent removal were recorded. Data were normalized to the control with 0 μM L-2-HG. **(h)** Temperature-stability analysis of sfLHGFR$_L$. Response of sfLHGFR$_L$ to 1 and 100 μM L-2-HG were determined at the indicated temperature values. Data were normalized to the initial ratio with 1 μM L-2-HG. **(i)** pH-stability analysis of sfLHGFR$_L$. Dose-response curves of sfLHGFR$_L$ for increasing concentrations (1 nM to 1 mM) of L-2-HG were determined at the indicated pH values. **(j)** Fluorescence intensities of sfLHGFR$_L$ at the indicated pH values. Data were normalized to the fluorescence at pH 7.4. **(k, l)** pH-correction of the fluorescence ratio of sfLHGFR$_L$ by cpSFYFP in the absence **(k)** or presence of 100 μM L-2-HG **(l)**. Data were normalized to the ratio at pH 7.4. All data shown are means ± s.d. (n ≥ 3 independent experiments).

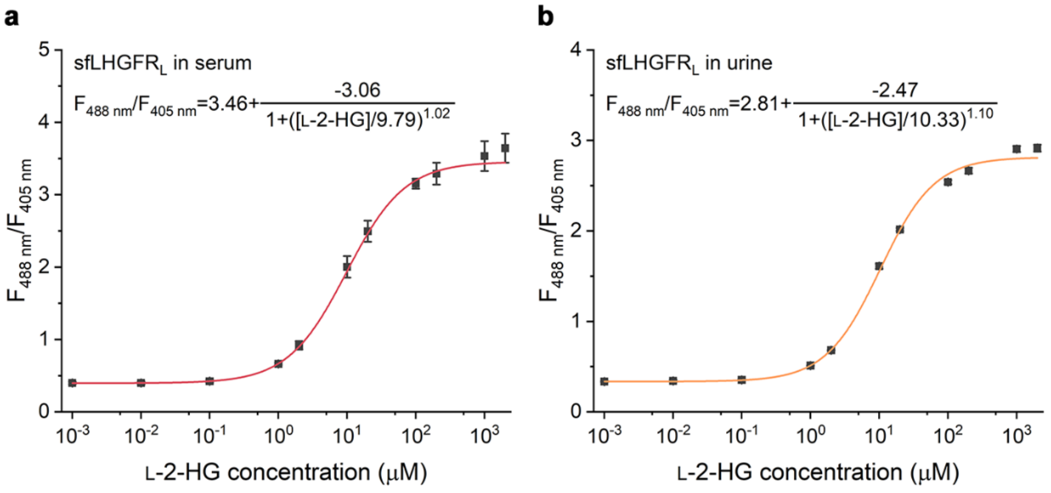

Figure S22 Standard curves for the quantitative detection of L-2-HG by sfLHGFR$_L$. (a, b) Standard curves for the quantitative detection of L-2-HG in human serum **(a)** and urine **(b)** samples by sfLHGFR$_L$. The concentrations of L-2-HG were calculated by substituting the detected fluorescence ratios into the fitted dose-response equations. The equations used for serum and urine L-2-HG quantification are separately presented in each figure.

Table S1 Comparison of the performance of different L-2-HG detection methods.

Properties	LC-MS/MS[a]	EaLHGFR-2[b]	$LHGFR_{0N3C}$[a]	$LHGFR_{0N7C}$[a]	$sfLHGFR_H$	$sfLHGFR_L$
Construction principle	/	Enzymatic assay	FRET	FRET	cpFP	cpFP
Application scenario	*In vitro*	*In vitro*	*In vitro* and *in vivo*	*In vitro* and *in vivo*	*In vitro* and *in vivo*	*In vitro* and *in vivo*[c]
Detection component	/	Enzyme and resazurin	Only biosensor	Only biosensor	Only biosensor	Only biosensor
Ratiometric readout	/	Incompatible	Compatible	Compatible	Compatible	Compatible
Response magnitude	/	2189.25 ± 26.89%	56.13 ± 0.29%	60.37 ± 1.30%	909.23 ± 16.19%	623.01 ± 8.52%
K_d[d]	/	ND[e]	29.33 ± 1.24 μM	7.22 ± 0.38 μM	181.77 ± 17.41 μM	9.94 ± 0.46 μM
LOD[f]	1 μM	0.042 μM	4.34 μM	0.70 μM	0.56 μM	0.14 μM
Detection Range	> 1 μM	0.042−5 μM	4.34−1,000 μM	0.70−100 μM	0.56−70,000 μM	0.14−100 μM
Response to L-2-HG in HEK293FT cells[g]	Incompatible	Incompatible	42.06 ± 2.06%	45.39 ± 1.89%	341.05 ± 54.12%	ND[e]

[a]The data were collected from *Nature Communications*, 2021, 12:3619 (DOI: 10.1038/s41467-021-23723-7).

[b]The data were collected from *Biosensors and Bioelectronics*, 2024, 243:115740 (DOI: 10.1016/j.bios.2023.115740).

[c]Although the *in vivo* availability of $sfLHGFR_L$ has not been demonstrated, the construction principle of this biosensor supports its *in vivo* application.

[d]K_d indicates apparent dissociation constant.

[e]ND indicates the data is not detected.

[f]The limit of detection (LOD) is calculated by interpolating the average background counts + 3 × standard deviation value.

[g]Fluorescence ratio changes of $LHGFR_{0N3C}$, $LHGFR_{0N7C}$, or $sfLHGFR_H$ expressed in HEK293FT cells in response to exogenous 10 mM L-2-HG addition.

Table S2 Pathology and L-2-HG concentration of seven patients with kidney cancer.

No.	Sex	Age	Diagnosis[a]	Serum L-2-HG concentration (μM)	Uinre L-2-HG concentration (μM)
Patient 1	Female	57	Left KIRC	0.89	51.11
Patient 2	Male	50	Left KIRC	0.70	28.00
Patient 3	Male	50	Right KIRC	1.13	90.64
Patient 4	Male	76	Right KIRC	1.13	48.30
Patient 5	Male	71	Right KIRC	1.13	24.34
Patient 6	Male	59	Left KIRC	0.48	18.99
Patient 7	Male	55	Right KIRC	0.96	27.71

[a]KIRC, kidney clear cell carcinoma.

Table S3 Strains and plasmids used in this study.

Strain or plasmid[a]	Relevant characteristics
**Strain**	
Escherichia coli DH5α	F⁻ φ80*lacZ*ΔM15 Δ(*lacZYA-argF*)U169 *deoR recA*1 *endA*1 *hsdR*17(r_K^-, m_K^+) *phoA supE*44 λ⁻ *thi*-1 *gyrA*96 *relA*1
E. coli DH5α-pME6032-F2-*lhgR*-F1-*lhgO*	*E. coli* DH5α carring plasmid pME6032-F2-*lhgR*-F1-*lhgO*
E. coli BL21(DE3)	F⁻ *ompT hsdSB*(*rB- mB-*) *gal*(λ *c I* 857 *ind1 Sam*7 *nin*5 *lac*UV5-T7*gene*1) *dcm* (DE3)
E. coli BL21(DE3)-$sfLHGFR_{LhgR\text{-}cpYFP\text{-}LhgR}$	*E. coli* BL21(DE3) carring plasmid pETDuet-$sfLHGFR_{LhgR\text{-}cpYFP\text{-}LhgR}$
E. coli BL21(DE3)-$sfLHGFR_{LhgR\text{-}cpYFP\text{-}LhgR(D2)}$	*E. coli* BL21(DE3) carring plasmid pETDuet-$sfLHGFR_{LhgR\text{-}cpYFP\text{-}LhgR(D2)}$
E. coli BL21(DE3)-$sfLHGFR_{LhgR(D2)\text{-}cpYFP\text{-}LhgR}$	*E. coli* BL21(DE3) carring plasmid pETDuet-$sfLHGFR_{LhgR(D2)\text{-}cpYFP\text{-}LhgR}$
E. coli BL21(DE3)-$sfLHGFR_{LhgR(D2)\text{-}cpYFP\text{-}LhgR(D2)}$	*E. coli* BL21(DE3) carring plasmid pETDuet-$sfLHGFR_{LhgR(D2)\text{-}cpYFP\text{-}LhgR(D2)}$
E. coli BL21(DE3)-$sfLHGFR_{114R/115R}$	*E. coli* BL21(DE3) carring plasmid pETDuet-$sfLHGFR_{114R/115R}$
E. coli BL21(DE3)-$sfLHGFR_{115R/116R}$	*E. coli* BL21(DE3) carring plasmid pETDuet-$sfLHGFR_{115R/116R}$
E. coli BL21(DE3)-$sfLHGFR_{116R/117D}$	*E. coli* BL21(DE3) carring plasmid pETDuet-$sfLHGFR_{116R/117D}$
E. coli BL21(DE3)-$sfLHGFR_{117D/118E}$	*E. coli* BL21(DE3) carring plasmid pETDuet-$sfLHGFR_{117D/118E}$
E. coli BL21(DE3)-$sfLHGFR_{133D/134K}$	*E. coli* BL21(DE3) carring plasmid pETDuet-$sfLHGFR_{133D/134K}$
E. coli BL21(DE3)-$sfLHGFR_{134K/135R}$	*E. coli* BL21(DE3) carring plasmid pETDuet-$sfLHGFR_{134K/135R}$
E. coli BL21(DE3)-$sfLHGFR_{135R/136S}$	*E. coli* BL21(DE3) carring plasmid pETDuet-$sfLHGFR_{135R/136S}$
E. coli BL21(DE3)-$sfLHGFR_{136S/137D}$	*E. coli* BL21(DE3) carring plasmid pETDuet-$sfLHGFR_{136S/137D}$
E. coli BL21(DE3)-$sfLHGFR_{137D/138F}$ ($sfLHGFR_H$-1)	*E. coli* BL21(DE3) carring plasmid pETDuet-$sfLHGFR_{137D/138F}$ ($sfLHGFR_H$-1)
E. coli BL21(DE3)-$sfLHGFR_{156S/157K}$	*E. coli* BL21(DE3) carring plasmid pETDuet-$sfLHGFR_{156S/157K}$

E. coli BL21(DE3)-sfLHGFR$_{157K/158N}$	*E. coli* BL21(DE3) carring plasmid pETDuet-sfLHGFR$_{157K/158N}$
E. coli BL21(DE3)-sfLHGFR$_{158N/159D}$	*E. coli* BL21(DE3) carring plasmid pETDuet-sfLHGFR$_{158N/159D}$
E. coli BL21(DE3)-sfLHGFR$_{159D/160Y}$	*E. coli* BL21(DE3) carring plasmid pETDuet-sfLHGFR$_{159D/160Y}$
E. coli BL21(DE3)-sfLHGFR$_{184A/185A}$	*E. coli* BL21(DE3) carring plasmid pETDuet-sfLHGFR$_{184A/185A}$
E. coli BL21(DE3)-sfLHGFR$_{185A/186H}$	*E. coli* BL21(DE3) carring plasmid pETDuet-sfLHGFR$_{185A/186H}$
E. coli BL21(DE3)-sfLHGFR$_{186H/187S}$	*E. coli* BL21(DE3) carring plasmid pETDuet-sfLHGFR$_{186H/187S}$
E. coli BL21(DE3)-sfLHGFR$_{187S/188V}$	*E. coli* BL21(DE3) carring plasmid pETDuet-sfLHGFR$_{187S/188V}$
E. coli BL21(DE3)-sfLHGFR$_{188V/189G}$	*E. coli* BL21(DE3) carring plasmid pETDuet-sfLHGFR$_{188V/189G}$
E. coli BL21(DE3)-sfLHGFR$_{189G/190G}$	*E. coli* BL21(DE3) carring plasmid pETDuet-sfLHGFR$_{189G/190G}$
E. coli BL21(DE3)-sfLHGFR$_{190G/191S}$	*E. coli* BL21(DE3) carring plasmid pETDuet-sfLHGFR$_{190G/191S}$
E. coli BL21(DE3)-sfLHGFR$_{191S/192A}$	*E. coli* BL21(DE3) carring plasmid pETDuet-sfLHGFR$_{191S/192A}$
E. coli BL21(DE3)-sfLHGFR$_{207D/208G}$	*E. coli* BL21(DE3) carring plasmid pETDuet-sfLHGFR$_{207D/208G}$
E. coli BL21(DE3)-sfLHGFR$_{208G/209D}$	*E. coli* BL21(DE3) carring plasmid pETDuet-sfLHGFR$_{208G/209D}$
E. coli BL21(DE3)-sfLHGFR$_{209D/210R}$	*E. coli* BL21(DE3) carring plasmid pETDuet-sfLHGFR$_{209D/210R}$
E. coli BL21(DE3)-sfLHGFR$_{228L/229K}$	*E. coli* BL21(DE3) carring plasmid pETDuet-sfLHGFR$_{228L/229K}$
E. coli BL21(DE3)-sfLHGFR$_{231E/232L}$ (sfLHGFR$_{L}$-1)	*E. coli* BL21(DE3) carring plasmid pETDuet-sfLHGFR$_{231E/232L}$ (sfLHGFR$_{L}$-1)
E. coli BL21(DE3)-sfLHGFR$_{H}$-1-0N1C	*E. coli* BL21(DE3) carring plasmid pETDuet-sfLHGFR$_{H}$-1-0N1C
E. coli BL21(DE3)-sfLHGFR$_{H}$-1-0N2C	*E. coli* BL21(DE3) carring plasmid pETDuet-sfLHGFR$_{H}$-1-0N2C
E. coli BL21(DE3)-sfLHGFR$_{H}$-1-0N3C	*E. coli* BL21(DE3) carring plasmid pETDuet-sfLHGFR$_{H}$-1-0N3C
E. coli BL21(DE3)-sfLHGFR$_{H-}$1-1N0C	*E. coli* BL21(DE3) carring plasmid pETDuet-sfLHGFR$_{H}$-1-1N0C
E. coli BL21(DE3)-sfLHGFR$_{H}$-1-1N1C	*E. coli* BL21(DE3) carring plasmid pETDuet-sfLHGFR$_{H}$-1-1N1C

E. coli BL21(DE3)-sfLHGFR$_H$-1-1N2C	*E. coli* BL21(DE3) carring plasmid pETDuet-sfLHGFR$_H$-1-1N2C
E. coli BL21(DE3)-sfLHGFR$_H$-1-1N3C	*E. coli* BL21(DE3) carring plasmid pETDuet-sfLHGFR$_H$-1-1N3C
E. coli BL21(DE3)-sfLHGFR$_H$-1-2N0C	*E. coli* BL21(DE3) carring plasmid pETDuet-sfLHGFR$_H$-1-2N0C
E. coli BL21(DE3)-sfLHGFR$_H$-1-2N1C	*E. coli* BL21(DE3) carring plasmid pETDuet-sfLHGFR$_H$-1-2N1C
E. coli BL21(DE3)-sfLHGFR$_H$-1-2N2C	*E. coli* BL21(DE3) carring plasmid pETDuet-sfLHGFR$_H$-1-2N2C
E. coli BL21(DE3)-sfLHGFR$_H$-1-2N3C	*E. coli* BL21(DE3) carring plasmid pETDuet-sfLHGFR$_H$-1-2N3C
E. coli BL21(DE3)-sfLHGFR$_H$-1-3N0C	*E. coli* BL21(DE3) carring plasmid pETDuet-sfLHGFR$_H$-1-3N0C
E. coli BL21(DE3)-sfLHGFR$_H$-1-3N1C	*E. coli* BL21(DE3) carring plasmid pETDuet-sfLHGFR$_H$-1-3N1C
E. coli BL21(DE3)-sfLHGFR$_H$-1-3N2C	*E. coli* BL21(DE3) carring plasmid pETDuet-sfLHGFR$_H$-1-3N2C
E. coli BL21(DE3)-sfLHGFR$_H$-1-3N3C	*E. coli* BL21(DE3) carring plasmid pETDuet-sfLHGFR$_H$-1-3N3C
E. coli BL21(DE3)-sfLHGFR$_H$-1-D2-0N0C	*E. coli* BL21(DE3) carring plasmid pETDuet-sfLHGFR$_H$-1-D2-0N0C
E. coli BL21(DE3)-sfLHGFR$_H$-1-D2-0N1C	*E. coli* BL21(DE3) carring plasmid pETDuet-sfLHGFR$_H$-1-D2-0N1C
E. coli BL21(DE3)-sfLHGFR$_H$-1-D2-0N2C	*E. coli* BL21(DE3) carring plasmid pETDuet-sfLHGFR$_H$-1-D2-0N2C
E. coli BL21(DE3)-sfLHGFR$_H$-1-D2-0N3C	*E. coli* BL21(DE3) carring plasmid pETDuet-sfLHGFR$_H$-1-D2-0N3C
E. coli BL21(DE3)-sfLHGFR$_H$-1-D2-1N0C (sfLHGFR$_H$-2)	*E. coli* BL21(DE3) carring plasmid pETDuet-sfLHGFR$_H$-1-D2-1N0C (sfLHGFR$_H$-2)
E. coli BL21(DE3)-sfLHGFR$_H$-1-D2-1N1C	*E. coli* BL21(DE3) carring plasmid pETDuet-sfLHGFR$_H$-1-D2-1N1C
E. coli BL21(DE3)-sfLHGFR$_H$-1-D2-1N2C	*E. coli* BL21(DE3) carring plasmid pETDuet-sfLHGFR$_H$-1-D2-1N2C
E. coli BL21(DE3)-sfLHGFR$_H$-1-D2-1N3C	*E. coli* BL21(DE3) carring plasmid pETDuet-sfLHGFR$_H$-1-D2-1N3C
E. coli BL21(DE3)-sfLHGFR$_H$-1-D2-2N0C	*E. coli* BL21(DE3) carring plasmid pETDuet-sfLHGFR$_H$-1-D2-2N0C
E. coli BL21(DE3)-sfLHGFR$_H$-1-D2-2N1C	*E. coli* BL21(DE3) carring plasmid pETDuet-sfLHGFR$_H$-1-D2-2N1C
E. coli BL21(DE3)-sfLHGFR$_H$-1-D2-2N2C	*E. coli* BL21(DE3) carring plasmid pETDuet-sfLHGFR$_H$-1-D2-2N2C

E. coli BL21(DE3)-sfLHGFR$_H$-1-D2-2N3C	*E. coli* BL21(DE3) carring plasmid pETDuet-sfLHGFR$_H$-1-D2-2N3C
E. coli BL21(DE3)-sfLHGFR$_H$-1-D2-3N0C	*E. coli* BL21(DE3) carring plasmid pETDuet-sfLHGFR$_H$-1-D2-3N0C
E. coli BL21(DE3)-sfLHGFR$_H$-1-D2-3N1C	*E. coli* BL21(DE3) carring plasmid pETDuet-sfLHGFR$_H$-1-D2-3N1C
E. coli BL21(DE3)-sfLHGFR$_H$-1-D2-3N2C	*E. coli* BL21(DE3) carring plasmid pETDuet-sfLHGFR$_H$-1-D2-3N2C
E. coli BL21(DE3)-sfLHGFR$_H$-1-D2-3N3C	*E. coli* BL21(DE3) carring plasmid pETDuet-sfLHGFR$_H$-1-D2-3N3C
E. coli BL21(DE3)-sfLHGFR$_H$-2-cpBFP	*E. coli* BL21(DE3) carring plasmid pETDuet-sfLHGFR$_H$-2-cpBFP
E. coli BL21(DE3)-sfLHGFR$_H$-2-cpTFP	*E. coli* BL21(DE3) carring plasmid pETDuet-sfLHGFR$_H$-2-cpTFP
E. coli BL21(DE3)-sfLHGFR$_H$-2-cpGFP	*E. coli* BL21(DE3) carring plasmid pETDuet-sfLHGFR$_H$-2-cpGFP
E. coli BL21(DE3)-sfLHGFR$_H$-2-cpEGFP	*E. coli* BL21(DE3) carring plasmid pETDuet-sfLHGFR$_H$-2-cpEGFP
E. coli BL21(DE3)-sfLHGFR$_H$-2-cpSFGFP	*E. coli* BL21(DE3) carring plasmid pETDuet-sfLHGFR$_H$-2-cpSFGFP
E. coli BL21(DE3)-sfLHGFR$_H$-2-cpVenus	*E. coli* BL21(DE3) carring plasmid pETDuet-sfLHGFR$_H$-2-cpVenus
E. coli BL21(DE3)-sfLHGFR$_H$-2-cpmOrange	*E. coli* BL21(DE3) carring plasmid pETDuet-sfLHGFR$_H$-2-cpmOrange
E. coli BL21(DE3)-sfLHGFR$_H$-2-cpmApple	*E. coli* BL21(DE3) carring plasmid pETDuet-sfLHGFR$_H$-2-cpmApple
E. coli BL21(DE3)-sfLHGFR$_H$-2-mCherry	*E. coli* BL21(DE3) carring plasmid pETDuet-sfLHGFR$_H$-2-mCherry
E. coli BL21(DE3)-sfLHGFR$_H$-2-cpmKate	*E. coli* BL21(DE3) carring plasmid pETDuet-sfLHGFR$_H$-2-cpmKate
E. coli BL21(DE3)-sfLHGFR$_H$-2-cpSFYFP (sfLHGFR$_H$-3)	*E. coli* BL21(DE3) carring plasmid pETDuet-sfLHGFR$_H$-2-cpSFYFP (sfLHGFR$_H$-3)
E. coli BL21(DE3)-sfLHGFR$_H$-3-#6	*E. coli* BL21(DE3) carring plasmid pETDuet-sfLHGFR$_H$-3-#6
E. coli BL21(DE3)-sfLHGFR$_H$-3-#15 (sfLHGFR$_H$)	*E. coli* BL21(DE3) carring plasmid pETDuet-sfLHGFR$_H$-3-#15 (sfLHGFR$_H$)
E. coli BL21(DE3)-sfLHGFR$_H$-3-#45	*E. coli* BL21(DE3) carring plasmid pETDuet-sfLHGFR$_H$-3-#45
E. coli BL21(DE3)-sfLHGFR$_H$-3-#55	*E. coli* BL21(DE3) carring plasmid pETDuet-sfLHGFR$_H$-3-#55
E. coli BL21(DE3)-sfLHGFR$_H$-3-#196	*E. coli* BL21(DE3) carring plasmid pETDuet-sfLHGFR$_H$-3-#196

E. coli BL21(DE3)-sfLHGFR$_H$-3-#296	*E. coli* BL21(DE3) carring plasmid pETDuet-sfLHGFR$_H$-3-#296
E. coli BL21(DE3)-sfLHGFR$_H$-3-#490	*E. coli* BL21(DE3) carring plasmid pETDuet-sfLHGFR$_H$-3-#490
E. coli BL21(DE3)-sfLHGFR$_H$-3-#501	*E. coli* BL21(DE3) carring plasmid pETDuet-sfLHGFR$_H$-3-#501
E. coli BL21(DE3)-sfLHGFR$_H$-3-#503	*E. coli* BL21(DE3) carring plasmid pETDuet-sfLHGFR$_H$-3-#503
E. coli BL21(DE3)-sfLHGFR$_H$-3-#704	*E. coli* BL21(DE3) carring plasmid pETDuet-sfLHGFR$_H$-3-#704
E. coli BL21(DE3)-sfLHGFR$_H$-3-#731	*E. coli* BL21(DE3) carring plasmid pETDuet-sfLHGFR$_H$-3-#731
E. coli BL21(DE3)-sfLHGFR$_H$-3-#739	*E. coli* BL21(DE3) carring plasmid pETDuet-sfLHGFR$_H$-3-#739
E. coli BL21(DE3)-cpSFYFP	*E. coli* BL21(DE3) carring plasmid pET28a-cpSFYFP
E. coli BL21(DE3)-sfLHGFR$_L$-1-D2 (sfLHGFR$_L$-2)	*E. coli* BL21(DE3) carring plasmid pETDuet-sfLHGFR$_L$-1-D2 (sfLHGFR$_L$-2)
E. coli BL21(DE3)-sfLHGFR$_L$-2-cpSFYFP (sfLHGFR$_L$-3)	*E. coli* BL21(DE3) carring plasmid pETDuet-sfLHGFR$_L$-2-cpSFYFP (sfLHGFR$_L$-3)
E. coli BL21(DE3)-sfLHGFR$_L$-3-#152 (sfLHGFR$_L$)	*E. coli* BL21(DE3) carring plasmid pETDuet-sfLHGFR$_L$-3-#152 (sfLHGFR$_L$)
E. coli DH5α-pcDNA3.1$^{(+)}$-sfLHGFR$_H$-2	*E. coli* DH5α carring mammalian expression plasmid pcDNA3.1$^{(+)}$-sfLHGFR$_H$-2
E. coli DH5α-pcDNA3.1$^{(+)}$-sfLHGFR$_H$-3	*E. coli* DH5α carring mammalian expression plasmid pcDNA3.1$^{(+)}$-sfLHGFR$_H$-3
E. coli DH5α-pcDNA3.1$^{(+)}$-sfLHGFR$_H$-3-#6	*E. coli* DH5α carring mammalian expression plasmid pcDNA3.1$^{(+)}$-sfLHGFR$_H$-3-#6
E. coli DH5α-pcDNA3.1$^{(+)}$-sfLHGFR$_H$-3-#15 (sfLHGFR$_H$)	*E. coli* DH5α carring mammalian expression plasmid pcDNA3.1$^{(+)}$-sfLHGFR$_H$-3-#15 (sfLHGFR$_H$)
E. coli DH5α-pcDNA3.1$^{(+)}$-sfLHGFR$_H$-3-#45	*E. coli* DH5α carring mammalian expression plasmid pcDNA3.1$^{(+)}$-sfLHGFR$_H$-3-#45
E. coli DH5α-pcDNA3.1$^{(+)}$-sfLHGFR$_H$-3-#55	*E. coli* DH5α carring mammalian expression plasmid pcDNA3.1$^{(+)}$-sfLHGFR$_H$-3-#55
E. coli DH5α-pcDNA3.1$^{(+)}$-sfLHGFR$_H$-3-#196	*E. coli* DH5α carring mammalian expression plasmid pcDNA3.1$^{(+)}$-sfLHGFR$_H$-3-#196
E. coli DH5α-pcDNA3.1$^{(+)}$-sfLHGFR$_H$-3-#296	*E. coli* DH5α carring mammalian expression plasmid pcDNA3.1$^{(+)}$-sfLHGFR$_H$-3-#296
E. coli DH5α-pcDNA3.1$^{(+)}$-sfLHGFR$_H$-3-#490	*E. coli* DH5α carring mammalian expression plasmid pcDNA3.1$^{(+)}$-sfLHGFR$_H$-3-#490
E. coli DH5α-pcDNA3.1$^{(+)}$-sfLHGFR$_H$-3-#501	*E. coli* DH5α carring mammalian expression plasmid pcDNA3.1$^{(+)}$-sfLHGFR$_H$-3-#501

E. coli DH5α-pcDNA3.1$^{(+)}$-sfLHGFR$_{H}$-3-#503	*E. coli* DH5α carring mammalian expression plasmid pcDNA3.1$^{(+)}$-sfLHGFR$_{H}$-3-#503
E. coli DH5α-pcDNA3.1$^{(+)}$-sfLHGFR$_{H}$-3-#704	*E. coli* DH5α carring mammalian expression plasmid pcDNA3.1$^{(+)}$-sfLHGFR$_{H}$-3-#704
E. coli DH5α-pcDNA3.1$^{(+)}$-sfLHGFR$_{H}$-3-#731	*E. coli* DH5α carring mammalian expression plasmid pcDNA3.1$^{(+)}$-sfLHGFR$_{H}$-3-#731
E. coli DH5α-pcDNA3.1$^{(+)}$-sfLHGFR$_{H}$-3-#739	*E. coli* DH5α carring mammalian expression plasmid pcDNA3.1$^{(+)}$-sfLHGFR$_{H}$-3-#739
E. coli DH5α-pcDNA3.1$^{(+)}$-cpSFYFP	*E. coli* DH5α carring mammalian expression plasmid pcDNA3.1$^{(+)}$-cpSFYFP
E. coli DH5α-pcDNA3.1$^{(+)}$-Mito-sfLHGFR$_{H}$	*E. coli* DH5α carring mammalian expression plasmid pcDNA3.1$^{(+)}$-Mito-sfLHGFR$_{H}$
E. coli DH5α-pcDNA3.1$^{(+)}$-Mito-cpSFYFP	*E. coli* DH5α carring mammalian expression plasmid pcDNA3.1$^{(+)}$-Mito-cpSFYFP
E. coli DH5α-pcDNA3.1$^{(+)}$-Cyto-sfLHGFR$_{H}$	*E. coli* DH5α carring mammalian expression plasmid pcDNA3.1$^{(+)}$-Cyto-sfLHGFR$_{H}$
E. coli DH5α-pcDNA3.1$^{(+)}$-Cyto-cpSFYFP	*E. coli* DH5α carring mammalian expression plasmid pcDNA3.1$^{(+)}$-Cyto-cpSFYFP
E. coli DH5α-pcDNA3.1$^{(+)}$-Nuc-sfLHGFR$_{H}$	*E. coli* DH5α carring mammalian expression plasmid pcDNA3.1$^{(+)}$-Nuc-sfLHGFR$_{H}$
E. coli DH5α-pcDNA3.1$^{(+)}$-Nuc-cpSFYFP	*E. coli* DH5α carring mammalian expression plasmid pcDNA3.1$^{(+)}$-Nuc-cpSFYFP
**Plasmid**	
pME6032-F2-*lhgR*-F1-*lhgO*	pME6032 contained F2-*lhgR*-F1-*lhgO* gene segment of *Pseudomonas putida* W619; Tet^{r}
pCDFDuet-cpYFP	pCDFDuet-1 contained the gene of cpYFP; Spe^{r}
pETDuet-1	Vector for protein expression in bacteria; Ap^{r}
pETDuet-sfLHGFR$_{LhgR\text{-}cpYFP\text{-}LhgR}$	pETDuet-1 contained the gene of sfLHGFR$_{LhgR\text{-}cpYFP\text{-}LhgR}$
pETDuet-sfLHGFR$_{LhgR\text{-}cpYFP\text{-}LhgR(D2)}$	pETDuet-1 contained the gene of sfLHGFR$_{LhgR\text{-}cpYFP\text{-}LhgR(D2)}$
pETDuet-sfLHGFR$_{LhgR(D2)\text{-}cpYFP\text{-}LhgR}$	pETDuet-1 contained the gene of sfLHGFR$_{LhgR(D2)\text{-}cpYFP\text{-}LhgR}$
pETDuet-sfLHGFR$_{LhgR(D2)\text{-}cpYFP\text{-}LhgR(D2)}$	pETDuet-1 contained the gene of sfLHGFR$_{LhgR(D2)\text{-}cpYFP\text{-}LhgR(D2)}$
pETDuet-sfLHGFR$_{114R/115R}$	pETDuet-1 contained the gene of sfLHGFR$_{114R/115R}$
pETDuet-sfLHGFR$_{115R/116R}$	pETDuet-1 contained the gene of sfLHGFR$_{115R/116R}$

pETDuet-sfLHGFR$_{116R/117D}$	pETDuet-1 contained the gene of sfLHGFR$_{116R/117D}$
pETDuet-sfLHGFR$_{117D/118E}$	pETDuet-1 contained the gene of sfLHGFR$_{117D/118E}$
pETDuet-sfLHGFR$_{133D/134K}$	pETDuet-1 contained the gene of sfLHGFR$_{133D/134K}$
pETDuet-sfLHGFR$_{134K/135R}$	pETDuet-1 contained the gene of sfLHGFR$_{134K/135R}$
pETDuet-sfLHGFR$_{135R/136S}$	pETDuet-1 contained the gene of sfLHGFR$_{135R/136S}$
pETDuet-sfLHGFR$_{136S/137D}$	pETDuet-1 contained the gene of sfLHGFR$_{136S/137D}$
pETDuet-sfLHGFR$_{137D/138F}$ (sfLHGFR$_{H}$-1)	pETDuet-1 contained the gene of sfLHGFR$_{137D/138F}$ (sfLHGFR$_{H}$-1)
pETDuet-sfLHGFR$_{156S/157K}$	pETDuet-1 contained the gene of sfLHGFR$_{156S/157K}$
pETDuet-sfLHGFR$_{157K/158N}$	pETDuet-1 contained the gene of sfLHGFR$_{157K/158N}$
pETDuet-sfLHGFR$_{158N/159D}$	pETDuet-1 contained the gene of sfLHGFR$_{158N/159D}$
pETDuet-sfLHGFR$_{159D/160Y}$	pETDuet-1 contained the gene of sfLHGFR$_{159D/160Y}$
pETDuet-sfLHGFR$_{184A/185A}$	pETDuet-1 contained the gene of sfLHGFR$_{184A/185A}$
pETDuet-sfLHGFR$_{185A/186H}$	pETDuet-1 contained the gene of sfLHGFR$_{185A/186H}$
pETDuet-sfLHGFR$_{186H/187S}$	pETDuet-1 contained the gene of sfLHGFR$_{186H/187S}$
pETDuet-sfLHGFR$_{187S/188V}$	pETDuet-1 contained the gene of sfLHGFR$_{187S/188V}$
pETDuet-sfLHGFR$_{188V/189G}$	pETDuet-1 contained the gene of sfLHGFR$_{188V/189G}$
pETDuet-sfLHGFR$_{189G/190G}$	pETDuet-1 contained the gene of sfLHGFR$_{189G/190G}$
pETDuet-sfLHGFR$_{190G/191S}$	pETDuet-1 contained the gene of sfLHGFR$_{190G/191S}$
pETDuet-sfLHGFR$_{191S/192A}$	pETDuet-1 contained the gene of sfLHGFR$_{191S/192A}$
pETDuet-sfLHGFR$_{207D/208G}$	pETDuet-1 contained the gene of sfLHGFR$_{207D/208G}$
pETDuet-sfLHGFR$_{208G/209D}$	pETDuet-1 contained the gene of sfLHGFR$_{208G/209D}$

pETDuet-sfLHGFR$_{209D/210R}$	pETDuet-1 contained the gene of sfLHGFR$_{209D/210R}$
pETDuet-sfLHGFR$_{228L/229K}$	pETDuet-1 contained the gene of sfLHGFR$_{228L/229K}$
pETDuet-sfLHGFR$_{231E/232L}$ (sfLHGFR$_{L}$-1)	pETDuet-1 contained the gene of sfLHGFR$_{231E/232L}$ (sfLHGFR$_{L}$-1)
pETDuet-sfLHGFR$_{H}$-1-0N1C	pETDuet-1 contained the gene of sfLHGFR$_{H}$-1-0N1C
pETDuet-sfLHGFR$_{H}$-1-0N2C	pETDuet-1 contained the gene of sfLHGFR$_{H}$-1-0N2C
pETDuet-sfLHGFR$_{H}$-1-0N3C	pETDuet-1 contained the gene of sfLHGFR$_{H}$-1-0N3C
pETDuet-sfLHGFR$_{H}$-1-1N0C	pETDuet-1 contained the gene of sfLHGFR$_{H}$-1-1N0C
pETDuet-sfLHGFR$_{H}$-1-1N1C	pETDuet-1 contained the gene of sfLHGFR$_{H}$-1-1N1C
pETDuet-sfLHGFR$_{H}$-1-1N2C	pETDuet-1 contained the gene of sfLHGFR$_{H}$-1-1N2C
pETDuet-sfLHGFR$_{H}$-1-1N3C	pETDuet-1 contained the gene of sfLHGFR$_{H}$-1-1N3C
pETDuet-sfLHGFR$_{H}$-1-2N0C	pETDuet-1 contained the gene of sfLHGFR$_{H}$-1-2N0C
pETDuet-sfLHGFR$_{H}$-1-2N1C	pETDuet-1 contained the gene of sfLHGFR$_{H}$-1-2N1C
pETDuet-sfLHGFR$_{H}$-1-2N2C	pETDuet-1 contained the gene of sfLHGFR$_{H}$-1-2N2C
pETDuet-sfLHGFR$_{H}$-1-2N3C	pETDuet-1 contained the gene of sfLHGFR$_{H}$-1-2N3C
pETDuet-sfLHGFR$_{H}$-1-3N0C	pETDuet-1 contained the gene of sfLHGFR$_{H}$-1-3N0C
pETDuet-sfLHGFR$_{H}$-1-3N1C	pETDuet-1 contained the gene of sfLHGFR$_{H}$-1-3N1C
pETDuet-sfLHGFR$_{H}$-1-3N2C	pETDuet-1 contained the gene of sfLHGFR$_{H}$-1-3N2C
pETDuet-sfLHGFR$_{H}$-1-3N3C	pETDuet-1 contained the gene of sfLHGFR$_{H}$-1-3N3C
pETDuet-sfLHGFR$_{H}$-1-D2-0N0C	pETDuet-1 contained the gene of sfLHGFR$_{H}$-1-D2-0N0C
pETDuet-sfLHGFR$_{H}$-1-D2-0N1C	pETDuet-1 contained the gene of sfLHGFR$_{H}$-1-D2-0N1C
pETDuet-sfLHGFR$_{H}$-1-D2-0N2C	pETDuet-1 contained the gene of sfLHGFR$_{H}$-1-D2-0N2C

pETDuet-sfLHGFR$_{H}$-1-D2-0N3C	pETDuet-1 contained the gene of sfLHGFR$_{H}$-1-D2-0N3C
pETDuet-sfLHGFR$_{H}$-1-D2-1N0C (sfLHGFR$_{H}$-2)	pETDuet-1 contained the gene of sfLHGFR$_{H}$-1-D2-1N0C (sfLHGFR$_{H}$-2)
pETDuet-sfLHGFR$_{H}$-1-D2-1N1C	pETDuet-1 contained the gene of sfLHGFR$_{H}$-1-D2-1N1C
pETDuet-sfLHGFR$_{H}$-1-D2-1N2C	pETDuet-1 contained the gene of sfLHGFR$_{H}$-1-D2-1N2C
pETDuet-sfLHGFR$_{H}$-1-D2-1N3C	pETDuet-1 contained the gene of sfLHGFR$_{H}$-1-D2-1N3C
pETDuet-sfLHGFR$_{H}$-1-D2-2N0C	pETDuet-1 contained the gene of sfLHGFR$_{H}$-1-D2-2N0C
pETDuet-sfLHGFR$_{H}$-1-D2-2N1C	pETDuet-1 contained the gene of sfLHGFR$_{H}$-1-D2-2N1C
pETDuet-sfLHGFR$_{H}$-1-D2-2N2C	pETDuet-1 contained the gene of sfLHGFR$_{H}$-1-D2-2N2C
pETDuet-sfLHGFR$_{H}$-1-D2-2N3C	pETDuet-1 contained the gene of sfLHGFR$_{H}$-1-D2-2N3C
pETDuet-sfLHGFR$_{H}$-1-D2-3N0C	pETDuet-1 contained the gene of sfLHGFR$_{H}$-1-D2-3N0C
pETDuet-sfLHGFR$_{H}$-1-D2-3N1C	pETDuet-1 contained the gene of sfLHGFR$_{H}$-1-D2-3N1C
pETDuet-sfLHGFR$_{H}$-1-D2-3N2C	pETDuet-1 contained the gene of sfLHGFR$_{H}$-1-D2-3N2C
pETDuet-sfLHGFR$_{H}$-1-D2-3N3C	pETDuet-1 contained the gene of sfLHGFR$_{H}$-1-D2-3N3C
pETDuet-sfLHGFR$_{H}$-2-cpBFP	pETDuet-1 contained the gene of sfLHGFR$_{H}$-2-cpBFP
pETDuet-sfLHGFR$_{H}$-2-cpTFP	pETDuet-1 contained the gene of sfLHGFR$_{H}$-2-cpTFP
pETDuet-sfLHGFR$_{H}$-2-cpGFP	pETDuet-1 contained the gene of sfLHGFR$_{H}$-2-cpGFP
pETDuet-sfLHGFR$_{H}$-2-cpEGFP	pETDuet-1 contained the gene of sfLHGFR$_{H}$-2-cpEGFP
pETDuet-sfLHGFR$_{H}$-2-cpSFGFP	pETDuet-1 contained the gene of sfLHGFR$_{H}$-2-cpSFGFP
pETDuet-sfLHGFR$_{H}$-2-cpVenus	pETDuet-1 contained the gene of sfLHGFR$_{H}$-2-cpVenus
pETDuet-sfLHGFR$_{H}$-2-cpmOrange	pETDuet-1 contained the gene of sfLHGFR$_{H}$-2-cpmOrange
pETDuet-sfLHGFR$_{H}$-2-cpmApple	pETDuet-1 contained the gene of sfLHGFR$_{H}$-2-cpmApple

pETDuet-sfLHGFR$_H$-2-mCherry	pETDuet-1 contained the gene of sfLHGFR$_H$-2-mCherry
pETDuet-sfLHGFR$_H$-2-cpmKate	pETDuet-1 contained the gene of sfLHGFR$_H$-2-cpmKate
pETDuet-sfLHGFR$_H$-2-cpSFYFP (sfLHGFR$_H$-3)	pETDuet-1 contained the gene of sfLHGFR$_H$-2-cpSFYFP (sfLHGFR$_H$-3)
pETDuet-sfLHGFR$_H$-3-#6	pETDuet-1 contained the gene of sfLHGFR$_H$-3-#6
pETDuet-sfLHGFR$_H$-3-#15 (sfLHGFR$_H$)	pETDuet-1 contained the gene of sfLHGFR$_H$-3-#15 (sfLHGFR$_H$)
pETDuet-sfLHGFR$_H$-3-#45	pETDuet-1 contained the gene of sfLHGFR$_H$-3-#45
pETDuet-sfLHGFR$_H$-3-#55	pETDuet-1 contained the gene of sfLHGFR$_H$-3-#55
pETDuet-sfLHGFR$_H$-3-#196	pETDuet-1 contained the gene of sfLHGFR$_H$-3-#196
pETDuet-sfLHGFR$_H$-3-#296	pETDuet-1 contained the gene of sfLHGFR$_H$-3-#296
pETDuet-sfLHGFR$_H$-3-#490	pETDuet-1 contained the gene of sfLHGFR$_H$-3-#490
pETDuet-sfLHGFR$_H$-3-#501	pETDuet-1 contained the gene of sfLHGFR$_H$-3-#501
pETDuet-sfLHGFR$_H$-3-#503	pETDuet-1 contained the gene of sfLHGFR$_H$-3-#503
pETDuet-sfLHGFR$_H$-3-#704	pETDuet-1 contained the gene of sfLHGFR$_H$-3-#704
pETDuet-sfLHGFR$_H$-3-#731	pETDuet-1 contained the gene of sfLHGFR$_H$-3-#731
pETDuet-sfLHGFR$_H$-3-#739	pETDuet-1 contained the gene of sfLHGFR$_H$-3-#739
pET28a-cpSFYFP	pET28a$^{(+)}$ contained the gene of cpSFYFP; Km^r
pETDuet-sfLHGFR$_L$-1-D2 (sfLHGFR$_L$-2)	pETDuet-1 contained the gene of sfLHGFR$_L$-1-D2 (sfLHGFR$_L$-2)
pETDuet-sfLHGFR$_L$-2-cpSFYFP (sfLHGFR$_L$-3)	pETDuet-1 contained the gene of sfLHGFR$_L$-2-cpSFYFP (sfLHGFR$_L$-3)
pETDuet-sfLHGFR$_L$-3-#152 (sfLHGFR$_L$)	pETDuet-1 contained the gene of sfLHGFR$_L$-3-#152 (sfLHGFR$_L$)
pcDNA3.1$^{(+)}$	Vector for protein expression in mammalian cells; Ap^r
pcDNA3.1$^{(+)}$-sfLHGFR$_H$-2	pcDNA3.1$^{(+)}$ contained Kozak sequence and the gene of sfLHGFR$_H$-2

pcDNA3.1$^{(+)}$-sfLHGFR$_H$-3	pcDNA3.1$^{(+)}$ contained Kozak sequence and the gene of sfLHGFR$_H$-3
pcDNA3.1$^{(+)}$-sfLHGFR$_H$-3-#6	pcDNA3.1$^{(+)}$ contained Kozak sequence and the gene of sfLHGFR$_H$-3-#6
pcDNA3.1$^{(+)}$-sfLHGFR$_H$-3-#15 (sfLHGFR$_H$)	pcDNA3.1$^{(+)}$ contained Kozak sequence and the gene of sfLHGFR$_H$-3-#15 (sfLHGFR$_H$)
pcDNA3.1$^{(+)}$-sfLHGFR$_H$-3-#45	pcDNA3.1$^{(+)}$ contained Kozak sequence and the gene of sfLHGFR$_H$-3-#45
pcDNA3.1$^{(+)}$-sfLHGFR$_H$-3-#55	pcDNA3.1$^{(+)}$ contained Kozak sequence and the gene of sfLHGFR$_H$-3-#55
pcDNA3.1$^{(+)}$-sfLHGFR$_H$-3-#196	pcDNA3.1$^{(+)}$ contained Kozak sequence and the gene of sfLHGFR$_H$-3-#196
pcDNA3.1$^{(+)}$-sfLHGFR$_H$-3-#296	pcDNA3.1$^{(+)}$ contained Kozak sequence and the gene of sfLHGFR$_H$-3-#296
pcDNA3.1$^{(+)}$-sfLHGFR$_H$-3-#490	pcDNA3.1$^{(+)}$ contained Kozak sequence and the gene of sfLHGFR$_H$-3-#490
pcDNA3.1$^{(+)}$-sfLHGFR$_H$-3-#501	pcDNA3.1$^{(+)}$ contained Kozak sequence and the gene of sfLHGFR$_H$-3-#501
pcDNA3.1$^{(+)}$-sfLHGFR$_H$-3-#503	pcDNA3.1$^{(+)}$ contained Kozak sequence and the gene of sfLHGFR$_H$-3-#503
pcDNA3.1$^{(+)}$-sfLHGFR$_H$-3-#704	pcDNA3.1$^{(+)}$ contained Kozak sequence and the gene of sfLHGFR$_H$-3-#704
pcDNA3.1$^{(+)}$-sfLHGFR$_H$-3-#731	pcDNA3.1$^{(+)}$ contained Kozak sequence and the gene of sfLHGFR$_H$-3-#731
pcDNA3.1$^{(+)}$-sfLHGFR$_H$-3-#739	pcDNA3.1$^{(+)}$ contained Kozak sequence and the gene of sfLHGFR$_H$-3-#739
pcDNA3.1$^{(+)}$-cpSFYFP	pcDNA3.1$^{(+)}$ contained Kozak sequence and the gene of cpSFYFP
pcDNA3.1$^{(+)}$-Mito-sfLHGFR$_H$	pcDNA3.1$^{(+)}$ contained Kozak sequence, mitochondrial targeting sequence, and the gene of sfLHGFR$_H$
pcDNA3.1$^{(+)}$-Mito-cpSFYFP	pcDNA3.1$^{(+)}$ contained Kozak sequence, mitochondrial targeting sequence, and the gene of cpSFYFP
pcDNA3.1$^{(+)}$-Cyto-sfLHGFR$_H$	pcDNA3.1$^{(+)}$ contained Kozak sequence, nuclear-excluded targeting sequence, and the gene of sfLHGFR$_H$
pcDNA3.1$^{(+)}$-Cyto-cpSFYFP	pcDNA3.1$^{(+)}$ contained Kozak sequence, nuclear-excluded targeting sequence, and the gene of cpSFYFP
pcDNA3.1$^{(+)}$-Nuc-sfLHGFR$_H$	pcDNA3.1$^{(+)}$ contained Kozak sequence, the gene of sfLHGFR$_H$, and nuclear targeting sequence
pcDNA3.1$^{(+)}$-Nuc-cpSFYFP	pcDNA3.1$^{(+)}$ contained Kozak sequence, the gene of cpSFYFP, and nuclear targeting sequence

[a] Tet^r, tetracycline resistant; Spe^r, spectinomycin resistant; Ap^r, ampicillin resistant; Km^r, kanamycin resistant.

Table S4 Oligonucleotides used in this study.

Primer	Sequence (5’-3’)	Use
**Construction of sfLHGFR$_H$-1 and sfLHGFR$_L$-1 (Screening of insertion sites for cpYFP)**		
LhgR-F1	ATCACCACAGCCAGGATCCGATGCTAGAACTCCAGCGC	Amplification of amino acids of the upstream fragment of LhgR (forward)
LhgR-R3	CATTATGCGGCCGCAAGCTTTTAGTCGAGTGCAGGTAGT	Amplification of amino acids of the downstream fragment of LhgR (reverse)
LhgR-R1	TCGCTGTTGTAGCCTGCAGAGTCGAGTGCAGGTAGTTCT	Amplification of LhgR (reverse)
LhgR-F3	TGGAGTACAACGGTGGCTGTATGCTAGAACTCCAGCGC	Amplification of LhgR (forward)
D2-LhgR-F1	ATCACCACAGCCAGGATCCGATGCTGGTGCAGATGTTCG	Amplification of ligand-binding domain of LhgR (forward)
D2-LhgR-F3	TGGAGTACAACGGTGGCTGTCTGGTGCAGATGTTCGAAA	Amplification of ligand-binding domain of LhgR (reverse)
cpYFP-F2	TCTGCAGGCTACAACAGCGACAAC	Amplification of cpYFP (forward)
cpYFP-R2	ACAGCCACCGTTGTACTCCAGCTTG	Amplification of cpYFP (reverse)
114R/115R-R1	TCGCTGTTGTAGCCTGCAGAGCGCGCGGCGATGGCCGCT	Amplification of amino acids 1 to 114 of LhgR (reverse)
114R/115R-F3	TGGAGTACAACGGTGGCTGTCGACGTGACGAACACGATC	Amplification of amino acids 115 to 236 of LhgR (forward)
115R/116R-R1	TCGCTGTTGTAGCCTGCAGATCGGCGCGCGGCGATGGC	Amplification of amino acids 1 to 115 of LhgR (reverse)
115R/116R-F3	TGGAGTACAACGGTGGCTGTCGTGACGAACACGATCTGG	Amplification of amino acids 116 to 236 of LhgR (forward)
116R/117D-R1	TCGCTGTTGTAGCCTGCAGAACGTCGGCGCGCGGCGAT	Amplification of amino acids 1 to 116 of LhgR (reverse)
116R/117D-F3	TGGAGTACAACGGTGGCTGTGACGAACACGATCTGGCGA	Amplification of amino acids 117 to 236 of LhgR (forward)
117D/118E-R1	TCGCTGTTGTAGCCTGCAGAGTCACGTCGGCGCGCGGC	Amplification of amino acids 1 to 117 of LhgR (reverse)
117D/118E-F3	TGGAGTACAACGGTGGCTGTGAACACGATCTGGCGAAC	Amplification of amino acids 118 to 236 of LhgR (forward)
133D/134K-R1	CGCTGTTGTAGCCTGCAGAGTCGAGCATCTCCTGCAGGG	Amplification of amino acids 1 to 133 of LhgR (reverse)

133D/134K-F3	TGGAGTACAACGGTGGCTGTAAACGCAGCGACTTCGCCA	Amplification of amino acids 134 to 236 of LhgR (forward)
134K/135R-R1	TCGCTGTTGTAGCCTGCAGATTTGTCGAGCATCTCCT	Amplification of amino acids 1 to 134 of LhgR (reverse)
134K/135R-F3	TGGAGTACAACGGTGGCTGTCGCAGCGACTTCGCCACT	Amplification of amino acids 135 to 236 of LhgR (forward)
135R/136S-R1	TCGCTGTTGTAGCCTGCAGAGCGTTTGTCGAGCATCTCC	Amplification of amino acids 1 to 135 of LhgR (reverse)
135R/136S-F3	TGGAGTACAACGGTGGCTGTAGCGACTTCGCCACTGCCT	Amplification of amino acids 136 to 236 of LhgR (forward)
136S/137D-R1	TCGCTGTTGTAGCCTGCAGAGCTGCGTTTGTCGAGCAT	Amplification of amino acids 1 to 136 of LhgR (reverse)
136S/137D-F3	TGGAGTACAACGGTGGCTGTGACTTCGCCACTGCCTCGG	Amplification of amino acids 137 to 236 of LhgR (forward)
137D/138F-R1	TCGCTGTTGTAGCCTGCAGAGTCGCTGCGTTTGTCGAGC	Amplification of amino acids 1 to 137 of LhgR (reverse)
137D/138F-F3	TGGAGTACAACGGTGGCTGTTTCGCCACTGCCTCGGCAG	Amplification of amino acids 138 to 236 of LhgR (forward)
156S/157K-R1	TCGCTGTTGTAGCCTGCAGAGCTGGCTTCGGCGATGGCC	Amplification of amino acids 1 to 156 of LhgR (reverse)
156S/157K-F3	TGGAGTACAACGGTGGCTGTAAGAACGATTACTTCGTGG	Amplification of amino acids 157 to 236 of LhgR (forward)
157K/158N-R1	TCGCTGTTGTAGCCTGCAGACTTGCTGGCTTCGGCGAT	Amplification of amino acids 1 to 157 of LhgR (reverse)
157K/158N-F3	TGGAGTACAACGGTGGCTGTAACGATTACTTCGTGGCCT	Amplification of amino acids 158 to 236 of LhgR (forward)
158N/159D-R1	TCGCTGTTGTAGCCTGCAGAGTTCTTGCTGGCTTCGGCG	Amplification of amino acids 1 to 158 of LhgR (reverse)
158N/159D-F3	TGGAGTACAACGGTGGCTGTGATTACTTCGTGGCCTT	Amplification of amino acids 159 to 236 of LhgR (forward)
159D/160Y-R1	TCGCTGTTGTAGCCTGCAGAATCGTTCTTGCTGGCTTCG	Amplification of amino acids 1 to 159 of LhgR (reverse)
159D/160Y-F3	TGGAGTACAACGGTGGCTGTTACTTCGTGGCCTTCCAT	Amplification of amino acids 160 to 236 of LhgR (forward)
184A/185A-R1	TCGCTGTTGTAGCCTGCAGATGCGGAGTTTTCCCAGGCG	Amplification of amino acids 1 to 184 of LhgR (reverse)
184A/185A-F3	TGGAGTACAACGGTGGCTGTGCCCATTCGGTGGGCGGCT	Amplification of amino acids 185 to 236 of LhgR (forward)
185A/186H-R1	TCGCTGTTGTAGCCTGCAGAGGCTGCGGAGTTTTCCCAG	Amplification of amino acids 1 to 185 of LhgR (reverse)
185A/186H-F3	TGGAGTACAACGGTGGCTGTCATTCGGTGGGCGGCTCGG	Amplification of amino acids 186 to 236 of LhgR (forward)

186H/187S-R1	TCGCTGTTGTAGCCTGCAGAATGGGCTGCGGAGTTTTCC	Amplification of amino acids 1 to 186 of LhgR (reverse)
186H/187S-F3	TGGAGTACAACGGTGGCTGTTCGGTGGGCGGCTCGGC	Amplification of amino acids 187 to 236 of LhgR (forward)
187S/188V-R1	TCGCTGTTGTAGCCTGCAGACGAATGGGCTGCGGAGTTT	Amplification of amino acids 1 to 187 of LhgR (reverse)
187S/188V-F3	TGGAGTACAACGGTGGCTGTGTGGGCGGCTCGGCCGAAG	Amplification of amino acids 188 to 236 of LhgR (forward)
188V/189G-R1	TCGCTGTTGTAGCCTGCAGACACCGAATGGGCTGCGGA	Amplification of amino acids 1 to 188 of LhgR (reverse)
188V/189G-F3	TGGAGTACAACGGTGGCTGTGGCGGCTCGGCCGAAGCCA	Amplification of amino acids 189 to 236 of LhgR (forward)
189G/190G-R1	TCGCTGTTGTAGCCTGCAGAGCCCACCGAATGGGCTGC	Amplification of amino acids 1 to 189 of LhgR (reverse)
189G/190G-F3	TGGAGTACAACGGTGGCTGTGGCTCGGCCGAAGCCAATC	Amplification of amino acids 190 to 236 of LhgR (forward)
190G/191S-R1	TCGCTGTTGTAGCCTGCAGAGCCGCCCACCGAATGGGCT	Amplification of amino acids 1 to 190 of LhgR (reverse)
190G/191S-F3	TGGAGTACAACGGTGGCTGTTCGGCCGAAGCCAATCGC	Amplification of amino acids 191 to 236 of LhgR (forward)
191S/192A-R1	TCGCTGTTGTAGCCTGCAGACGAGCCGCCCACCGAATG	Amplification of amino acids 1 to 191 of LhgR (reverse)
191S/192A-F3	TGGAGTACAACGGTGGCTGTGCCGAAGCCAATCGCGA	Amplification of amino acids 192 to 236 of LhgR (forward)
207D/208G-R1	TCGCTGTTGTAGCCTGCAGAGTCGGCGATGGCCTGATAA	Amplification of amino acids 1 to 207 of LhgR (reverse)
207D/208G-F3	TGGAGTACAACGGTGGCTGTGGTGACCGCCAAAGGGCTG	Amplification of amino acids 208 to 236 of LhgR (forward)
208G/209D-R1	TCGCTGTTGTAGCCTGCAGAACCGTCGGCGATGGCCTGA	Amplification of amino acids 1 to 208 of LhgR (reverse)
208G/209D-F3	TGGAGTACAACGGTGGCTGTGACCGCCAAAGGGCTGCG	Amplification of amino acids 209 to 236 of LhgR (forward)
209D/210R-R1	TCGCTGTTGTAGCCTGCAGAGTCACCGTCGGCGATGGC	Amplification of amino acids 1 to 209 of LhgR (reverse)
209D/210R-F3	TGGAGTACAACGGTGGCTGTCGCCAAAGGGCTGCGGCGT	Amplification of amino acids 210 to 236 of LhgR (forward)
228L/229K-R1	TCGCTGTTGTAGCCTGCAGACAGGCGTTTGGCAGAGGCG	Amplification of amino acids 1 to 228 of LhgR (reverse)
228L/229K-F2	CATTATGCGGCCGCAAGCTTTTAGTCGAGTGCAGGTAGTT CTATTTTACAGCCACCGTTGTACTCCAGCTTGTGC	Amplification of cpYFP and amino acids 229 to 236 of LhgR (reverse)
231E/232L-R1	TCGCTGTTGTAGCCTGCAGATTCTATTTTCAGGCGTTTG	Amplification of amino acids 1 to 231 of LhgR (reverse)

231E/232L-F2	CATTATGCGGCCGCAAGCTTTTAGTCGAGTGCAGGTAGAC AGCCACCGTTGTACTCCAGCTTGTGC	Amplification of cpYFP and amino acids 232 to 236 of LhgR (reverse)
**Construction of sfLHGFR$_H$-2 (Removal of the DNA-binding domain of LhgR and truncation of the linker between LhgR and cpYFP)**		
D2-LhgR-F	ATCACCACAGCCAGGATCCGATGCTGGTGCAGATGTTC	Amplification of sfLHGFR$_H$-1 with the DNA-binding domain of LhgR removed (forward)
D2-LhgR-R	CATTATGCGGCCGCAAGCTTTTAGTCGAGTGCAGGTAG	Amplification of sfLHGFR$_H$-1 with the DNA-binding domain of LhgR removed (reverse)
137D/138F-cpYFP-0N	TGCTCGACAAACGCAGCGACTCTGCAGGCTACAACAGCG	Amplification of cpYFP-0N of sfLHGFR$_H$-1 (forward)
137D/138F-cpYFP-1N	TGCTCGACAAACGCAGCGACGCAGGCTACAACAGCGACA	Amplification of cpYFP-1N of sfLHGFR$_H$-1 (forward)
137D/138F-cpYFP-2N	TGCTCGACAAACGCAGCGACGGCTACAACAGCGACAACG	Amplification of cpYFP-2N of sfLHGFR$_H$-1 (forward)
137D/138F-cpYFP-3N	TGCTCGACAAACGCAGCGACTACAACAGCGACAACGTCT	Amplification of cpYFP-3N of sfLHGFR$_H$-1 (forward)
137D/138F-cpYFP-0C	GCTGCCGAGGCAGTGGCGAAACAGCCACCGTTGTACTCC	Amplification of cpYFP-0C of sfLHGFR$_H$-1 (reverse)
137D/138F-cpYFP-1C	GCTGCCGAGGCAGTGGCGAAGCCACCGTTGTACTCCAGC	Amplification of cpYFP-1C of sfLHGFR$_H$-1 (reverse)
137D/138F-cpYFP-2C	GCTGCCGAGGCAGTGGCGAAACCGTTGTACTCCAGCTTG	Amplification of cpYFP-2C of sfLHGFR$_H$-1 (reverse)
137D/138F-cpYFP-3C	GCTGCCGAGGCAGTGGCGAAGTTGTACTCCAGCTTGTGC	Amplification of cpYFP-3C of sfLHGFR$_H$-1 (reverse)
137D/138F-inverse PCR-F	GTCGCTGCGTTTGTCG	Inverse amplification of the plasmid backbone of sfLHGFR$_H$-1 (forward)
137D/138F-inverse PCR-R	TTCGCCACTGCCTCGG	Inverse amplification of the plasmid backbone of sfLHGFR$_H$-1 (reverse)
**Construction of sfLHGFR$_H$-3 (Replacement of cpYFP with different fluorescent proteins and introducing superfolder mutations in cpYFP)**		
cpBFP-F	TGCTCGACAAACGCAGCGACGCAGGCAACGTTTACATCAA G	Amplification of cpBFP (forward)
cpBFP-R	GCTGCCGAGGCAGTGGCGAAACAGCCACCATTATATTCCA GTTT	Amplification of cpBFP (reverse)
cpTFP-F	TGCTCGACAAACGCAGCGACGCAGGCAACGTGTATATCAT G	Amplification of cpTFP (forward)
cpTFP-R	GCTGCCGAGGCAGTGGCGAAACAGCCACCGTTATATTCCA GTTT	Amplification of cpTFP (reverse)

cpGFP-F	TGCTCGACAAACGCAGCGACGCAGGCAACGTGTATATCAAG	Amplification of cpGFP (forward)
cpGFP-R	GCTGCCGAGGCAGTGGCGAAACAGCCACCATTATATTCCAGTTT	Amplification of cpGFP (reverse)
cpEGFP-F	TGCTCGACAAACGCAGCGACGCAGGCAACGTTTACATCAAG	Amplification of cpEGFP (forward)
cpEGFP-R	GCTGCCGAGGCAGTGGCGAAACAGCCACCGTTATATTCCAGTTT	Amplification of cpEGFP (reverse)
cpSFGFP-F	TGCTCGACAAACGCAGCGACGCAGGCAGTCATAACGTGTA	Amplification of cpSFGFP (forward)
cpSFGFP-R	GCTGCCGAGGCAGTGGCGAAACAGCCACCAAAGTTATATTCCAG	Amplification of cpSFGFP (reverse)
cpVenus-F	TGCTCGACAAACGCAGCGACGCAGGCTACAACAGTGATAAC	Amplification of cpVenus (forward)
cpVenus-R	GCTGCCGAGGCAGTGGCGAAACAGCCACCATTATATTCCAGTTT	Amplification of cpVenus (reverse)
cpmOrange-F	TGCTCGACAAACGCAGCGACGCAGGCGTGAGTGAACGTATG	Amplification of cpmOrange (forward)
cpmOrange-R	GCTGCCGAGGCAGTGGCGAAACAGCCACCTGCTTCCCAACCCAT	Amplification of cpmOrange (reverse)
cpmApple-F	TGCTCGACAAACGCAGCGACGCAGGCGTGAGCGAACGTATG	Amplification of cpmApple (forward)
cpmApple-R	GCTGCCGAGGCAGTGGCGAAACAGCCACCGGCTTCCCAACCCAT	Amplification of cpmApple (reverse)
mCherry-F	TGCTCGACAAACGCAGCGACGCAGGCATGGTTAGTAAGGGT	Amplification of mCherry (forward)
mCherry-R	GCTGCCGAGGCAGTGGCGAAACAGCCACCTTTATACAGTTCATC	Amplification of mCherry (reverse)
cpmKate-F	TGCTCGACAAACGCAGCGACGCAGGCATGGGTGGCCGTAGTA	Amplification of cpmKate (forward)
cpmKate-R	GCTGCCGAGGCAGTGGCGAAACAGCCACCTTTTTTACTGCGATA	Amplification of cpmKate (reverse)
cpSFYFP-inverse PCR-F	CACGCTGAACTTGTGGC	Inverse amplification of the plasmid backbone of sfLHGFR$_H$-2 (forward)
cpSFYFP-inverse PCR-R	TACAAGACCCGCGCCGA	Inverse amplification of the plasmid backbone of sfLHGFR$_H$-2 (reverse)

cpSFYFP-F1	ACGGCCACAAGTTCAGCGTGCGCGGCGAGGGCGAGGGCG ATGCCACCAACGGCAAGCTGACCCTG	Amplification of cpYFP with the introduction of four superfolder mutations (cpSFYFP) (Primer 1)
cpSFYFP-R1	ACCTCGGCGCGGGTCTTGTAGGTGCCGTCGTCCTTGAAGA AGA	Amplification of cpYFP with the introduction of four superfolder mutations (cpSFYFP) (Primer 2)
cpSFYFP-F2	TGCTCGACAAACGCAGCGACGCAGGCTTCAACAGCGACA ACGTCTATAT	Amplification of cpYFP with the introduction of four superfolder mutations (cpSFYFP) (Primer 3)
cpSFYFP-R2	GCTGCCGAGGCAGTGGCGAAACAGCCACCGTTGTACTCCA	Amplification of cpYFP with the introduction of four superfolder mutations (cpSFYFP) (Primer 4)
pET28a-cpSFYFP-F	GCAAATGGGTCGCGGATCCTTCAACAGCGACAACGTCTA	Amplification of cpSFYFP (forward)
pET28a-cpSFYFP-R	TGGTGGTGGTGCTCGAGTTAGTTGTACTCCAGCTTGTG	Amplification of cpSFYFP (reverse)
**Construction of sfLHGFR$_H$ (Random mutation of the linker)**		
0N-mutation	TGCTCGACAAACGCAGCGACNNBNNBNNBTTCAACAGCG ACAACGTCTA	Amplification of cpSFYFP with random linker (forward primer 1)
1N-mutation	TGCTCGACAAACGCAGCGACNNBNNBTTCAACAGCGACA ACGTCTA	Amplification of cpSFYFP with random linker (forward primer 2)
2N-mutation	TGCTCGACAAACGCAGCGACNNBTTCAACAGCGACAACGT CTA	Amplification of cpSFYFP with random linker (forward primer 3)
3N-mutation	TGCTCGACAAACGCAGCGACTTCAACAGCGACAACGTCTA	Amplification of cpSFYFP with random linker (forward primer 4)
0C-mutation	GCTGCCGAGGCAGTGGCGAAVNNVNNVNNGTTGTACTCC AGCTTGTGC	Amplification of cpSFYFP with random linker (reverse primer 1)
1C-mutation	GCTGCCGAGGCAGTGGCGAAVNNVNNGTTGTACTCCAGCT TGTGC	Amplification of cpSFYFP with random linker (reverse primer 2)
2C-mutation	GCTGCCGAGGCAGTGGCGAAVNNGTTGTACTCCAGCTTGT GC	Amplification of cpSFYFP with random linker (reverse primer 3)
3C-mutation	GCTGCCGAGGCAGTGGCGAAGTTGTACTCCAGCTTGTGC	Amplification of cpSFYFP with random linker (reverse primer 4)
**Construction of sfLHGFR$_L$-2, sfLHGFR$_L$-3, and sfLHGFR$_L$**		
D2-LhgR-F'	ACCACAGCCAGGATCCGATGCTGGTGCAGATGTTCGAAA	Amplification of sfLHGFR$_L$-1 with the DNA-binding domain of LhgR removed (forward)
D2-LhgR-R'	ATTCTATTTTCAGGCGTTTGGCAGAGGCGCGCAGATG	Amplification of sfLHGFR$_L$-1 with the DNA-binding domain of LhgR removed (reverse)

D2-inverse PCR-F’	CATCGGATCCTGGCTGTG	Inverse amplification of the plasmid backbone of sfLHGFR$_L$-1 (forward)
D2-inverse PCR-R’	CAAACGCCTGAAAATAGA	Inverse amplification of the plasmid backbone of sfLHGFR$_L$-1 (reverse)
cpSFYFP-F’	TGAAAATAGAATCTGCAGGCTTCAACAGCGACAACGTCT	Amplification of cpSFYFP for sfLHGFR$_L$-3 construction (forward)
cpSFYFP-R’	AGTGCAGGTAGACAGCCACCGTTGTACTCCAGCTTGT	Amplification of cpSFYFP for sfLHGFR$_L$-3 construction (reverse)
cpSFYFP-inverse PCR-F’	GCCTGCAGATTCTATTTT	Inverse amplification of the plasmid backbone of sfLHGFR$_L$-2 (forward)
cpSFYFP-inverse PCR-R’	GGTGGCTGTCTACCTGCA	Inverse amplification of the plasmid backbone of sfLHGFR$_L$-2 (reverse)
0N-mutation’	CCAAACGCCTGAAAATAGAANNBNNBNNBTTCAACAGCGACAACGTCTA	Amplification of cpSFYFP with random linker (forward primer 1)
1N-mutation’	CCAAACGCCTGAAAATAGAANNBNNBTTCAACAGCGACAACGTCTA	Amplification of cpSFYFP with random linker (forward primer 2)
2N-mutation’	CCAAACGCCTGAAAATAGAANNBTTCAACAGCGACAACGTCTA	Amplification of cpSFYFP with random linker (forward primer 3)
3N-mutation’	CCAAACGCCTGAAAATAGAATTCAACAGCGACAACGTCTA	Amplification of cpSFYFP with random linker (forward primer 4)
0C-mutation’	TTTTAGTCGAGTGCAGGTAGVNNVNNVNNGTTGTACTCCAGCTTGTG	Amplification of cpSFYFP with random linker (reverse primer 1)
1C-mutation’	TTTTAGTCGAGTGCAGGTAGVNNVNNGTTGTACTCCAGCTTGTG	Amplification of cpSFYFP with random linker (reverse primer 2)
2C-mutation’	TTTTAGTCGAGTGCAGGTAGVNNGTTGTACTCCAGCTTGTG	Amplification of cpSFYFP with random linker (reverse primer 3)
3C-mutation’	TTTTAGTCGAGTGCAGGTAGGTTGTACTCCAGCTTGTG	Amplification of cpSFYFP with random linker (reverse primer 4)
**Expression in mammalian cells**		
pcDNA3.1$^{(+)}$-cpSFYFP-inverse PCR-F	GCCGGCATCGGACCTCT	Inverse amplification of the plasmid backbone of pcDNA3.1$^{(+)}$-sfLHGFR$_H$-2 (forward)
pcDNA3.1$^{(+)}$-cpSFYFP-inverse PCR-R	TACAAGACCAGAGCCGA	Inverse amplification of the plasmid backbone of pcDNA3.1$^{(+)}$-sfLHGFR$_H$-2 (reverse)
pcDNA3.1$^{(+)}$-cpSFYFP-F1	ACAAGAGGTCCGATGCCGGCTTCAATAGCGATAACGTGTACA	Amplification of cpYFP with the introduction of four superfolder mutations (cpSFYFP) (Primer 1)

pcDNA3.1$^{(+)}$-cpSFYFP-R1	TTCAGTGTCAGCTTGCCATTGGTAGCATCGCCCTCGCCCTCGCCGCGCACGCTGAACTTGTGGCCGT	Amplification of cpYFP with the introduction of four superfolder mutations (cpSFYFP) (Primer 2)
pcDNA3.1$^{(+)}$-cpSFYFP-F2	ACGGCCACAAGTTCAGCGTGCGCGGCGAGGGCGAGGGCGATGCTACCAATGGCAAGCTGACACTGAA	Amplification of cpYFP with the introduction of four superfolder mutations (cpSFYFP) (Primer 3)
pcDNA3.1$^{(+)}$-cpSFYFP-R2	ACCTCGGCTCTGGTCTTGTAGGTGCCATCGTCCTTGAAAAAGATTGTTCTC	Amplification of cpYFP with the introduction of four superfolder mutations (cpSFYFP) (Primer 4)
pcDNA3.1$^{(+)}$-inverse PCR-F	ATCGGACCTCTTGTCCAG	Inverse amplification of the plasmid backbone of pcDNA3.1$^{(+)}$-sfLHGFR$_H$-3 (forward)
pcDNA3.1$^{(+)}$-inverse PCR-R	TTTGCCACCGCCTCCGCC	Inverse amplification of the plasmid backbone of pcDNA3.1$^{(+)}$-sfLHGFR$_H$-3 (reverse)
pcDNA3.1$^{(+)}$-#6-F	TGCTGGACAAGAGGTCCGATAGGATCTTCAATAGCGATAACGTGTA	Amplification of sfLHGFR$_H$-3-#6 (forward)
pcDNA3.1$^{(+)}$-#6-R	GCGGCGGAGGCGGTGGCAAAGCAGCCGCCATTGTACTCCAGCTTGTGGC	Amplification of sfLHGFR$_H$-3-#6 (reverse)
pcDNA3.1$^{(+)}$-#15-F	TGCTGGACAAGAGGTCCGATCCTGATTTCAATAGCGATAACGTGTA	Amplification of sfLHGFR$_H$-3-#15 (forward)
pcDNA3.1$^{(+)}$-#15-R	GCGGCGGAGGCGGTGGCAAAATGGGACAGATTGTACTCCAGCTTGTGGC	Amplification of sfLHGFR$_H$-3-#15 (reverse)
pcDNA3.1$^{(+)}$-#45-F	TGCTGGACAAGAGGTCCGATGGCGGCTTCAATAGCGATAACGTGTA	Amplification of sfLHGFR$_H$-3-#45 (forward)
pcDNA3.1$^{(+)}$-#45-R	GCGGCGGAGGCGGTGGCAAAGCAGCCGCCATTGTACTCCAGCTTGTGGC	Amplification of sfLHGFR$_H$-3-#45 (reverse)
pcDNA3.1$^{(+)}$-#55-F	TGCTGGACAAGAGGTCCGATAAGGTGTTCAATAGCGATAACGTGTA	Amplification of sfLHGFR$_H$-3-#55 (forward)
pcDNA3.1$^{(+)}$-#55-R	GCGGCGGAGGCGGTGGCAAAGCAGCCGCCATTGTACTCCAGCTTGTGGC	Amplification of sfLHGFR$_H$-3-#55 (reverse)
pcDNA3.1$^{(+)}$-#196-F	TGCTGGACAAGAGGTCCGATTTCCTGTCCTTCAATAGCGATAACGTGTA	Amplification of sfLHGFR$_H$-3-#196 (forward)
pcDNA3.1$^{(+)}$-#196-R	GCGGCGGAGGCGGTGGCAAAGCAGCCGCCATTGTACTCCAGCTTGTGGC	Amplification of sfLHGFR$_H$-3-#196 (reverse)
pcDNA3.1$^{(+)}$-#296-F	TGCTGGACAAGAGGTCCGATTGCCTGAACTTCAATAGCGATAACGTGTA	Amplification of sfLHGFR$_H$-3-#296 (forward)
pcDNA3.1$^{(+)}$-#296-R	GCGGCGGAGGCGGTGGCAAAGAACAGTGTATTGTACTCCAGCTTGTGGC	Amplification of sfLHGFR$_H$-3-#296 (reverse)

pcDNA3.1$^{(+)}$-#490-F	TGCTGGACAAGAGGTCCGATGTGCCTTTCAATAGCGATAACGTGTA	Amplification of sfLHGFR$_H$-3-#490 (forward)
pcDNA3.1$^{(+)}$-#490-R	GCGGCGGAGGCGGTGGCAAACAGGGAATTGTACTCCAGCTTGTGGC	Amplification of sfLHGFR$_H$-3-#490 (reverse)
pcDNA3.1$^{(+)}$-#501-F	TGCTGGACAAGAGGTCCGATATCTCCTTCAATAGCGATAACGTGTA	Amplification of sfLHGFR$_H$-3-#501 (forward)
pcDNA3.1$^{(+)}$-#501-R	GCGGCGGAGGCGGTGGCAAAGTACCTATTGTACTCCAGCTTGTGGC	Amplification of sfLHGFR$_H$-3-#501 (reverse)
pcDNA3.1$^{(+)}$-#503-F	TGCTGGACAAGAGGTCCGATATCGGCTTCAATAGCGATAACGTGTA	Amplification of sfLHGFR$_H$-3-#503 (forward)
pcDNA3.1$^{(+)}$-#503-R	GCGGCGGAGGCGGTGGCAAAGGTCCTATTGTACTCCAGCTTGTGGC	Amplification of sfLHGFR$_H$-3-#503 (reverse)
pcDNA3.1$^{(+)}$-#704-F	GCTGGACAAGAGGTCCGATAGGTTCAATAGCGATAACGTGTAC	Amplification of sfLHGFR$_H$-3-#704 (forward)
pcDNA3.1$^{(+)}$-#704-R	GCGGCGGAGGCGGTGGCAAAGATATTATTGTACTCCAGCTTGTGGC	Amplification of sfLHGFR$_H$-3-#704 (reverse)
pcDNA3.1$^{(+)}$-#731-F	TGCTGGACAAGAGGTCCGATTTCAATAGCGATAACGTGTA	Amplification of sfLHGFR$_H$-3-#731 (forward)
pcDNA3.1$^{(+)}$-#731-R	GCGGCGGAGGCGGTGGCAAAGGGGTCATTGTACTCCAGCTTGTGGC	Amplification of sfLHGFR$_H$-3-#731 (reverse)
pcDNA3.1$^{(+)}$-#739-F	TGCTGGACAAGAGGTCCGATTTCAATAGCGATAACGTGTA	Amplification of sfLHGFR$_H$-3-#739 (forward)
pcDNA3.1$^{(+)}$-#739-R	GCGGAGGCGGTGGCAAAGACGCTATTGTACTCCAGCTTGTGGC	Amplification of sfLHGFR$_H$-3-#739 (reverse)
pcDNA3.1$^{(+)}$-cpSFYFP-inverse PCR-f	GGTGGCGGATCCGAGCTC	Inverse amplification of the plasmid backbone of pcDNA3.1$^{(+)}$ for pcDNA3.1$^{(+)}$-cpSFYFP construction (forward)
pcDNA3.1$^{(+)}$-cpSFYFP-inverse PCR-r	TAATCTAGAGGGCCCGTT	Inverse amplification of the plasmid backbone of pcDNA3.1$^{(+)}$ for pcDNA3.1$^{(+)}$-cpSFYFP construction (reverse)
pcDNA3.1$^{(+)}$-cpSFYFP-f	CCGAGCTCGGATCCGCCACCATGTTCAATAGCGATAACG	Amplification of cpSFYFP (forward)
pcDNA3.1$^{(+)}$-cpSFYFP-r	AAACGGGCCCTCTAGATTAATTGTACTCCAGCTTGTGGC	Amplification of cpSFYFP (reverse)
**Expression inside mitochondria**		
Mito-sfLHGFR$_H$-inverse PCR-F	GGTGGCGGATCCGAGCTC	Inverse amplification of the plasmid backbone of pcDNA3.1$^{(+)}$-sfLHGFR$_H$ for Mito-sfLHGFR$_H$ construction (forward)

Mito-sfLHGFR$_H$-inverse PCR-R	ATGCTGGTGCAGATGTTC	Inverse amplification of the plasmid backbone of pcDNA3.1$^{(+)}$-sfLHGFR$_H$ for Mito-sfLHGFR$_H$ construction (reverse)
Mito-sfLHGFR$_H$-F	CGAGCTCGGATCCGCCACCATGCTATCACTGCGACAGTC	Amplification of sfLHGFR$_H$ for Mito-sfLHGFR$_H$ construction (forward)
Mito-sfLHGFR$_H$-R	CGAACATCTGCACCAGCATGAGTAGGTATCGCGATGAGC	Amplification of sfLHGFR$_H$ for Mito-sfLHGFR$_H$ construction (reverse)
Mito-cpSFYFP-inverse PCR-F	GGTGGCGGATCCGAGCTC	Inverse amplification of the plasmid backbone of pcDNA3.1$^{(+)}$-cpSFYFP for Mito-cpSFYFP construction (forward)
Mito-cpSFYFP-inverse PCR-R	TTCAATAGCGATAACGTG	Inverse amplification of the plasmid backbone of pcDNA3.1$^{(+)}$-cpSFYFP for Mito-cpSFYFP construction (reverse)
Mito-cpSFYFP-F	CGAGCTCGGATCCGCCACCATGCTATCACTGCGACAGTC	Amplification of cpSFYFP for Mito-cpSFYFP construction (forward)
Mito-cpSFYFP-R	ACACGTTATCGCTATTGAACATGAGTAGGTATCGCGATG	Amplification of cpSFYFP for Mito-cpSFYFP construction (reverse)
**Expression inside cytosol**		
Cyto-sfLHGFR$_H$-inverse PCR-F	GGTGGCGGATCCGAGCTC	Inverse amplification of the plasmid backbone of pcDNA3.1$^{(+)}$-sfLHGFR$_H$ for Cyto-sfLHGFR$_H$ construction (forward)
Cyto-sfLHGFR$_H$-inverse PCR-R	ATGCTGGTGCAGATGTTC	Inverse amplification of the plasmid backbone of pcDNA3.1$^{(+)}$-sfLHGFR$_H$ for Cyto-sfLHGFR$_H$ construction (reverse)
Cyto-sfLHGFR$_H$-F	CGAGCTCGGATCCGCCACCATGGCCTTGCAGAAGAAGCT	Amplification of sfLHGFR$_H$ for Cyto-sfLHGFR$_H$ construction (forward)
Cyto-sfLHGFR$_H$-R	CGAACATCTGCACCAGCATCAAATCCTCAAGGCGCTTTC	Amplification of sfLHGFR$_H$ for Cyto-sfLHGFR$_H$ construction (reverse)
Cyto-cpSFYFP-inverse PCR-F	GGTGGCGGATCCGAGCTC	Inverse amplification of the plasmid backbone of pcDNA3.1$^{(+)}$-cpSFYFP for Cyto-cpSFYFP construction (forward)
Cyto-cpSFYFP-inverse PCR-R	TTCAATAGCGATAACGTG	Inverse amplification of the plasmid backbone of pcDNA3.1$^{(+)}$-cpSFYFP for Cyto-cpSFYFP construction (reverse)
Cyto-cpSFYFP-F	GCTTGGTACCGAGCTCGGAT	Amplification of cpSFYFP for Cyto-cpSFYFP construction (forward)
Cyto-cpSFYFP-R	ACACGTTATCGCTATTGAACATCAAATCCTCAAGGCGCT	Amplification of cpSFYFP for Cyto-cpSFYFP construction (reverse)
**Expression inside nucleus**		
Nuc-sfLHGFR$_H$-inverse PCR-F	ATCCAGGGCGGGCAGCTC	Inverse amplification of the plasmid backbone of pcDNA3.1$^{(+)}$-sfLHGFR$_H$ for Nuc-sfLHGFR$_H$ construction (forward)

Nuc-sfLHGFR$_H$-inverse PCR-R	TAATCTAGAGGGCCCGTT	Inverse amplification of the plasmid backbone of pcDNA3.1$^{(+)}$-sfLHGFR$_H$ for Nuc-sfLHGFR$_H$ construction (reverse)
Nuc-sfLHGFR$_H$-F	CGAGCTGCCCGCCCTGGATGATCCAAAAAAGAAGAGAAA	Amplification of sfLHGFR$_H$ for Nuc-sfLHGFR$_H$ construction (forward)
Nuc-sfLHGFR$_H$-R	AAACGGGCCCTCTAGATTATACCTTTCTCTTCTTCTTTG	Amplification of sfLHGFR$_H$ for Nuc-sfLHGFR$_H$ construction (reverse)
Nuc-cpSFYFP-inverse PCR-F	ATTGTACTCCAGCTTGTG	Inverse amplification of the plasmid backbone of pcDNA3.1$^{(+)}$-cpSFYFP for Nuc-cpSFYFP construction (forward)
Nuc-cpSFYFP-inverse PCR-R	TAATCTAGAGGGCCCGTT	Inverse amplification of the plasmid backbone of pcDNA3.1$^{(+)}$-cpSFYFP for Nuc-cpSFYFP construction (reverse)
Nuc-cpSFYFP-F	GCCACAAGCTGGAGTACAATGATCCAAAAAAGAAGAGAA	Amplification of cpSFYFP for Nuc-cpSFYFP construction (forward)
Nuc-cpSFYFP-R	TCGAGGCTGATCAGCGGGTTTAAACGGGCCCTCTAGATT	Amplification of cpSFYFP for Nuc-cpSFYFP construction (reverse)
**qPCR (Mouse)**		
GAPDH-F	GGTTGTCTCCTGCGACTTCA	Analysis of GAPDH expression by qPCR (forward)
GAPDH-R	TGGTCCAGGGTTTCTTACTCC	Analysis of GAPDH expression by qPCR (reverse)
IL-1β-F	TGGCAACTGTTCCTGAACTC	Analysis of IL-1β expression by qPCR (forward)
IL-1β-R	GGAAGCAGCCCTTCATCTTT	Analysis of IL-1β expression by qPCR (reverse)
IL-6-F	ACTTCCATCCAGTTGCCTTCTTGG	Analysis of IL-6 expression by qPCR (forward)
IL-6-R	TTAAGCCTCCGACTTGTGAAGTGG	Analysis of IL-6 expression by qPCR (reverse)
Nos2-F	CCAAGCCCTCACCTACTTCC	Analysis of Nos2 expression by qPCR (forward)
Nos2-R	CTCTGAGGGCTGACACAAGG	Analysis of Nos2 expression by qPCR (reverse)
TNFα-F	ACGGCATGGATCTCAAAGAC	Analysis of TNFα expression by qPCR (forward)
TNFα-R	AGATAGCAAATCGGCTGACG	Analysis of TNFα expression by qPCR (reverse)
Arg1-F	CTCCAAGCCAAAGTCCTTAGAG	Analysis of Arg1 expression by qPCR (forward)
Arg1-R	AGGAGCTGTCATTAGGGACATC	Analysis of Arg1 expression by qPCR (reverse)

TGF-β-F	GCAACAATTCCTGGCGTTACCTTG	Analysis of TGF-β expression by qPCR (forward)
TGF-β-R	CAGCCACTGCCGTACAACTCC	Analysis of TGF-β expression by qPCR (reverse)
IL-10-F	AGGCGCTGTCATCG	Analysis of IL-10 expression by qPCR (forward)
IL-10-R	CACCTTGGTCTTGGAGCTTAT	Analysis of IL-10 expression by qPCR (reverse)
Mrc1-F	CTCTGTTCAGCTATTGGACGC	Analysis of Mrc1 expression by qPCR (forward)
Mrc1-R	CGGAATTTCTGGGATTCAGCTTC	Analysis of Mrc1 expression by qPCR (reverse)
LDHA-F	TGTCTCCAGCAAAGACTACTGT	Analysis of LDHA expression by qPCR (forward)
LDHA-R	GACTGTACTTGACAATGTTGGGA	Analysis of LDHA expression by qPCR (reverse)
MDH1-F	TTCTGGACGGTGTCCTGATG	Analysis of MDH1 expression by qPCR (forward)
MDH1-R	TTTCACATTGGCTTTCAGTAGGT	Analysis of MDH1 expression by qPCR (reverse)
MDH2-F	TTGGGCAACCCCTTTCACTC	Analysis of MDH2 expression by qPCR (forward)
MDH2-R	GCCTTTCACATTTGCTCTGGTC	Analysis of MDH2 expression by qPCR (reverse)
L2HGDH-F	TAGTCATCGTTGGAGGTGGAA	Analysis of L2HGDH expression by qPCR (forward)
L2HGDH-R	TCCAGTCTGGTGAAGAGCTAAAT	Analysis of L2HGDH expression by qPCR (reverse)
ME2-F	GGCTAAGAGCTGTTACCACTCC	Analysis of ME2 expression by qPCR (reverse)
ME2-R	CGTAAACGCCATTCCCTTGTT	Analysis of ME2 expression by qPCR (reverse)
**qPCR (Homo sapiens)**		
GAPDH-F	GGAGCGAGATCCCTCCAAAAT	Analysis of GAPDH expression by qPCR (forward)
GAPDH-R	GGCTGTTGTCATACTTCTCATGG	Analysis of GAPDH expression by qPCR (reverse)
L2HGDH-F	ATCGTTGGTGGCGGAATTGT	Analysis of L2HGDH expression by qPCR (forward)
L2HGDH-R	CCAGTCTGGTGAACAGCTAAAT	Analysis of L2HGDH expression by qPCR (reverse)

LDHA-F	TTGACCTACGTGGCTTGGAAG	Analysis of LDHA expression by qPCR (forward)
LDHA-R	GGTAACGGAATCGGGCTGAAT	Analysis of LDHA expression by qPCR (reverse)
MDH2-F	TCGGCCCAGAACAATGCTAAA	Analysis of MDH2 expression by qPCR (forward)
MDH2-R	GCGGCTTTGGTCTCGATGT	Analysis of MDH2 expression by qPCR (reverse)
ME2-F	ATATACACCGACGGTTGGTCT	Analysis of ME2 expression by qPCR (reverse)
ME2-R	CATCAGTCACTACAACAGCCTT	Analysis of ME2 expression by qPCR (reverse)
SLC1A1-F	GCGAGGAAAGGATGCGAGT	Analysis of SLC1A1 expression by qPCR (forward)
SLC1A1-R	GCTGTGTTCTCGAACCAAGACT	Analysis of SLC1A1 expression by qPCR (reverse)
